# A reliable computational workflow for the selection of optimal screening libraries

**DOI:** 10.1186/s13321-015-0108-0

**Published:** 2015-12-11

**Authors:** Yocheved Gilad, Katalin Nadassy, Hanoch Senderowitz

**Affiliations:** Department of Chemistry, Bar-Ilan University, Ramat-Gan, 52900 Israel; Dassault Systèmes BIOVIA, 334 Cambridge Science Park, Cambridge, CB4 0WN UK

**Keywords:** Diversity, Fingerprints, QSAR, Screening libraries, Similarity, Library selection

## Abstract

**Background:**

The experimental screening of compound collections is a common starting point in many drug discovery projects. Successes of such screening campaigns critically depend on the quality of the screened library. Many libraries are currently available from different vendors yet the selection of the optimal screening library for a specific project is challenging. We have devised a novel workflow for the rational selection of project-specific screening libraries.

**Results:**

The workflow accepts as input a set of virtual candidate libraries and applies the following steps to each library: (1) data curation; (2) assessment of ADME/T profile; (3) assessment of the number of promiscuous binders/frequent HTS hitters; (4) assessment of internal diversity; (5) assessment of similarity to known active compound(s) (optional); (6) assessment of similarity to in-house or otherwise accessible compound collections (optional). For ADME/T profiling, Lipinski’s and Veber’s rule-based filters were implemented and a new blood brain barrier permeation model was developed and validated (85 and 74 % success rate for training set and test set, respectively). Diversity and similarity descriptors which demonstrated best performances in terms of their ability to select either diverse or focused sets of compounds from three databases (Drug Bank, CMC and CHEMBL) were identified and used for diversity and similarity assessments. The workflow was used to analyze nine common screening libraries available from six vendors. The results of this analysis are reported for each library providing an assessment of its quality. Furthermore, a consensus approach was developed to combine the results of these analyses into a single score for selecting the optimal library under different scenarios.

**Conclusions:**

We have devised and tested a new workflow for the rational selection of screening libraries under different scenarios. The current workflow was implemented using the Pipeline Pilot software yet due to the usage of generic components, it can be easily adapted and reproduced by computational groups interested in rational selection of screening libraries. Furthermore, the workflow could be readily modified to include additional components. This workflow has been routinely used in our laboratory for the selection of libraries in multiple projects and consistently selects libraries which are well balanced across multiple parameters.

**Graphical abstract:**
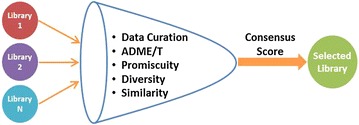
.

**Electronic supplementary material:**

The online version of this article (doi:10.1186/s13321-015-0108-0) contains supplementary material, which is available to authorized users.

## Background

The experimental screening of compound collections is a widely used starting point in the search for new biologically active compounds. Many screening libraries are currently available either in the public domain or from commercial vendors making an exhaustive screening impossible [1, 2]. Thus, it is important to develop rational strategies for the selection of the optimal screening library and chemoinformatic approaches can be used for this purpose.

Many factors should be considered while selecting an optimal screening library. Some (e.g., price, compounds availability, time for shipment, and vendor reliability) are vendor-dependent and will not be further discussed. Other factors could be computationally analyzed based on compounds structures. Such an analysis requires data curation since wrong structures are likely to lead to a faulty analysis. Indeed, available databases have been shown to include multiple flawed structures (up to 10 %) [3–7].

First, libraries should be selected based on the planned screening campaign. Screening campaigns could be largely divided into two categories, namely, focused (or biased) and unbiased. If the structure of the biological target is known a focused screening library could be designed, for example, through docking simulations. Similarly, if active compounds are known, ligand based methods could be used to select a screening library which includes additional compounds similar to them. Similarity could be assessed in several ways including: (1) pharmacophore-based which is particularly useful for identifying compounds with the same chemical features as the active compounds yet with new chemotypes or chemical scaffolds [8, 9], (2) fingerprints-based and (3) substructure-based. However, focusing the screening library entirely on active compounds might be problematic in particular when only few, structurally similar active compounds are known or when the identity of the biological target is unknown (see below). In such cases, maintaining diversity within the screening library may identify compounds with new scaffolds or compounds acting through different mechanisms.

Diversity is especially important when neither the structure of the biological target nor the structures of its ligands are known and more so if the precise identity of the target is unknown. Such cases require screening at the functional or phenotype levels and based on the similar property principle [10], are likely to benefit from biologically testing a diverse set of compounds. Chemical diversity is typically assessed using pairwise distances between library members in a pre-defined descriptors space. Multiple descriptors and distance metrics were evaluated for their ability to select diverse subsets from parent databases. In particular, two-dimensional (2D) fingerprints coupled with the Tanimoto coefficient as the distance metric were shown to give good results in multiple cases [11].

Aside from diversity/similarity considerations, other factors should be considered. In particular, absorption, distribution, metabolism, excretion, and toxicity (ADME/T) profiles are important for both hit identification and lead optimization [12–15]. Hence evaluating ADME/T properties (e.g., adherence to Lipinski’s “rule of five” [16] or Veber’s rules [17], oral bio-availability, lack of toxic group [18] or other properties calculated by means of QSAR models [19–21]) across a screening library is a useful criterion for library selection. In addition, promiscuous binders or frequent HTS hitters should be avoided [22] since these are likely to turn up as false positive upon hit validation. Such consideration formed the basis for several compounds removal filters [23–26].

Finally, an additional consideration for library selection could be invoked, namely, similarity to in-house compound collections. Assessing the overlap between a library candidate for purchasing and in-house available compound collections is critical to avoid duplicates and to assess whether the candidate and available libraries cover similar parts of the chemistry space. Depending on the specific project, a library may be selected to fill “holes” in chemistry space or to improve coverage of regions already occupied by the in-house library.

*This work focuses on the selection of whole libraries for phenotypic screening.* Our interest in this challenge emerged from our involvement in multiple screening projects targeting rare diseases such as Leukoencephalopathy with vanishing white matter (VWM disease) [27], the neurodegenerative amyotrophic lateral sclerosis (ALS) disease [28], and cystic fibrosis (CF) [29]. In all of these projects the selection of a screening library was hampered by lack of information on the identity or the structure of the biological target or on active compounds.

Some chemoinformatic tools required to address the issues described above have been described in the literature. Similarly, multiple descriptors have been evaluated for their ability to select either diverse or focused sets of compounds [30–33]. However, these tools were not combined into a unified workflow for the ranking and subsequent selection of screening libraries based on multiple criteria. With this in mind we have developed such a workflow consisting of the following steps: (1) data curation; (2) ADME/T profiling; (3) assessment of promiscuous binders/frequent HTS hitters; (4) assessment of internal diversity; (5) assessment of similarity to known reference compounds; (6) assessment of similarity to in-house available compound collections. For step (2) we have included as library characteristic adherence to Lipinski’s and Veber’s rules and as an important component of the ADME/T profiling, we have developed and validated a new blood brain barrier permeation model. This model was developed due to our involvement in multiple projects requiring blood brain barrier permeating compounds. Other models could be similarly developed based on the specific requirements of other projects. For step (3) we have implemented a filter based on substructures of known promiscuous binders/frequent HTS hitters. For step (4), 25 two-dimensional descriptor sets (fingerprints) were evaluated for their ability to select diverse subsets of compounds from within the Drug Bank, CMC or CHEMBL databases. Diversity was estimated as coverage of target (Drug Bank, CHEMBL) or indication (CMC) spaces. The best “diversity descriptors” were incorporated into the workflow. For step (5) the same descriptors were evaluated for their ability to identify known active compounds based on their similarity to a reference active compound from the three databases. The best “similarity descriptors” were incorporated into the workflow. These similarity descriptors were also utilized in step (6). Library ranking was based on a simple consensus approach considering all the above parameters. As a proof of concept, this workflow was used to evaluate nine common libraries available from six vendors and to select a library with the most balanced profile in terms of all these parameters.

## Results

**Fig. 1:**
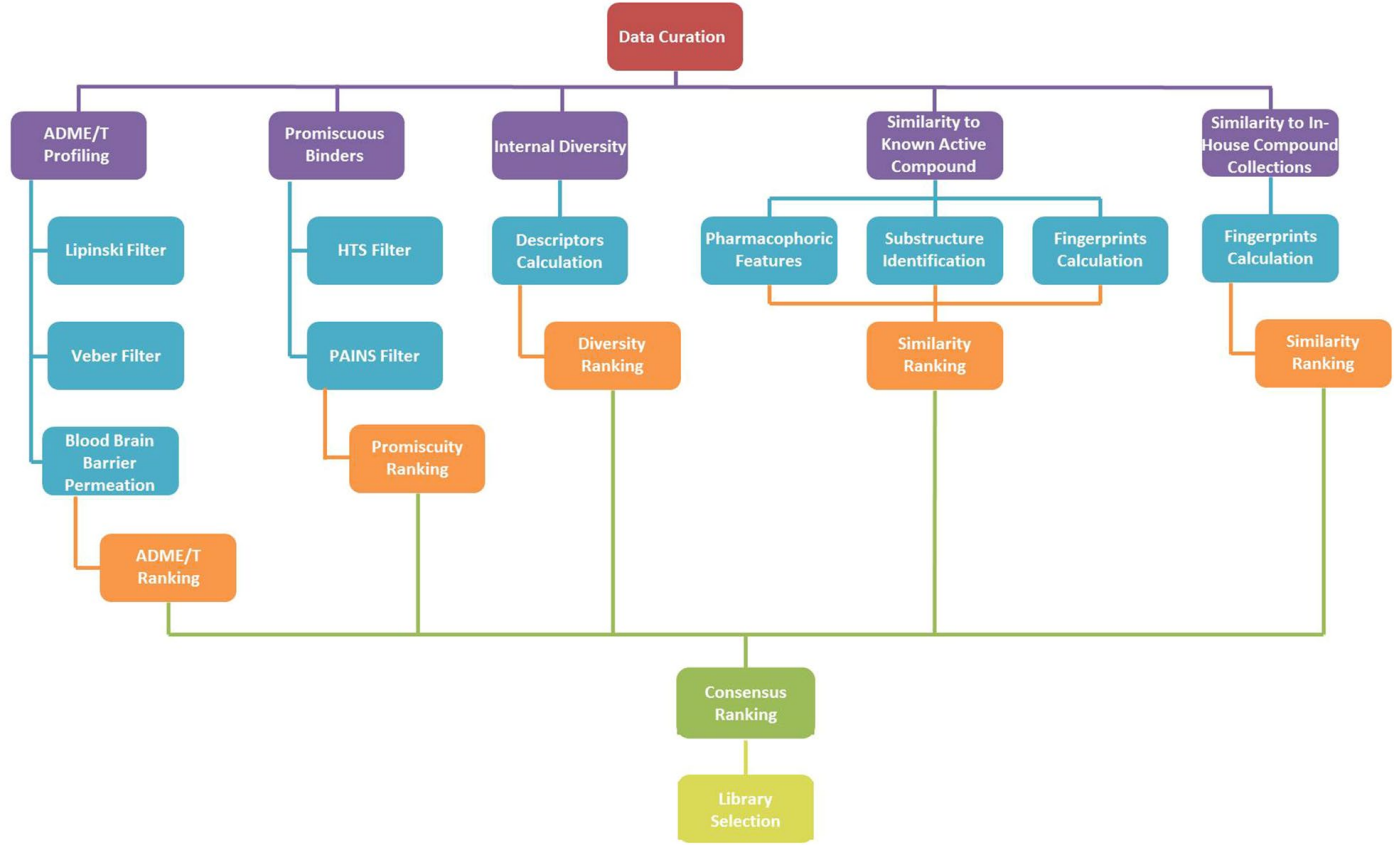
Outline of the workflow

An overview of the workflow is presented in Fig. 1.

### LogBB model

The performances of the best logBB QSAR model on training and test compounds are presented in Additional file 1: Figure S1a, b, respectively and are overall satisfactory (R_train_^2^ = 0.66; R_test_^2^ = 0.67). The QSAR equation takes the form:

$$\begin{aligned} \log BB & = 1.2827 + 0.17977 \times {\text{AlogP}}98 - 0.0033777 \times {\text{DSPA}}1 - 0.18676 \\ & \quad \times {\text{Num}}\_{\text{H}}\_{\text{Acceptors}} + 0.1557 \times {\text{S}}_{\text{sssN}} - 0.022135 \times \left\langle4.6743 - {\text{S}}_{{{\text{ssCH}}2}} \right\rangle \\ \end{aligned}$$

where AlogP98 is an atom-type based log partition coefficient, DPSA1 is the difference between the positive solvent-accessible area and the negative one, Num_H_Acceptors is the number of hydrogen bond acceptors and SsssN and SssCH2 are specific electrotopological state indices.

Transferring the quantitative predictions into qualitative ones (i.e., logBB ≥ 0, BBB permeable; logBB < 0, BBB impermeable) leads to success rates of 85 and 74 % for training set and test set, respectively. Positively charged, negatively charged and neutral compounds are predicted by the model with similar accuracies making it applicable to multiple charge states. Finally, the results of Y-scrambling (R_train_^2^: 0.07 ± 0.07; R_test_^2^: 0.18 ± 0.28) demonstrate the lack of chance correlation.

### Selection of diversity descriptors

**Fig. 2:**
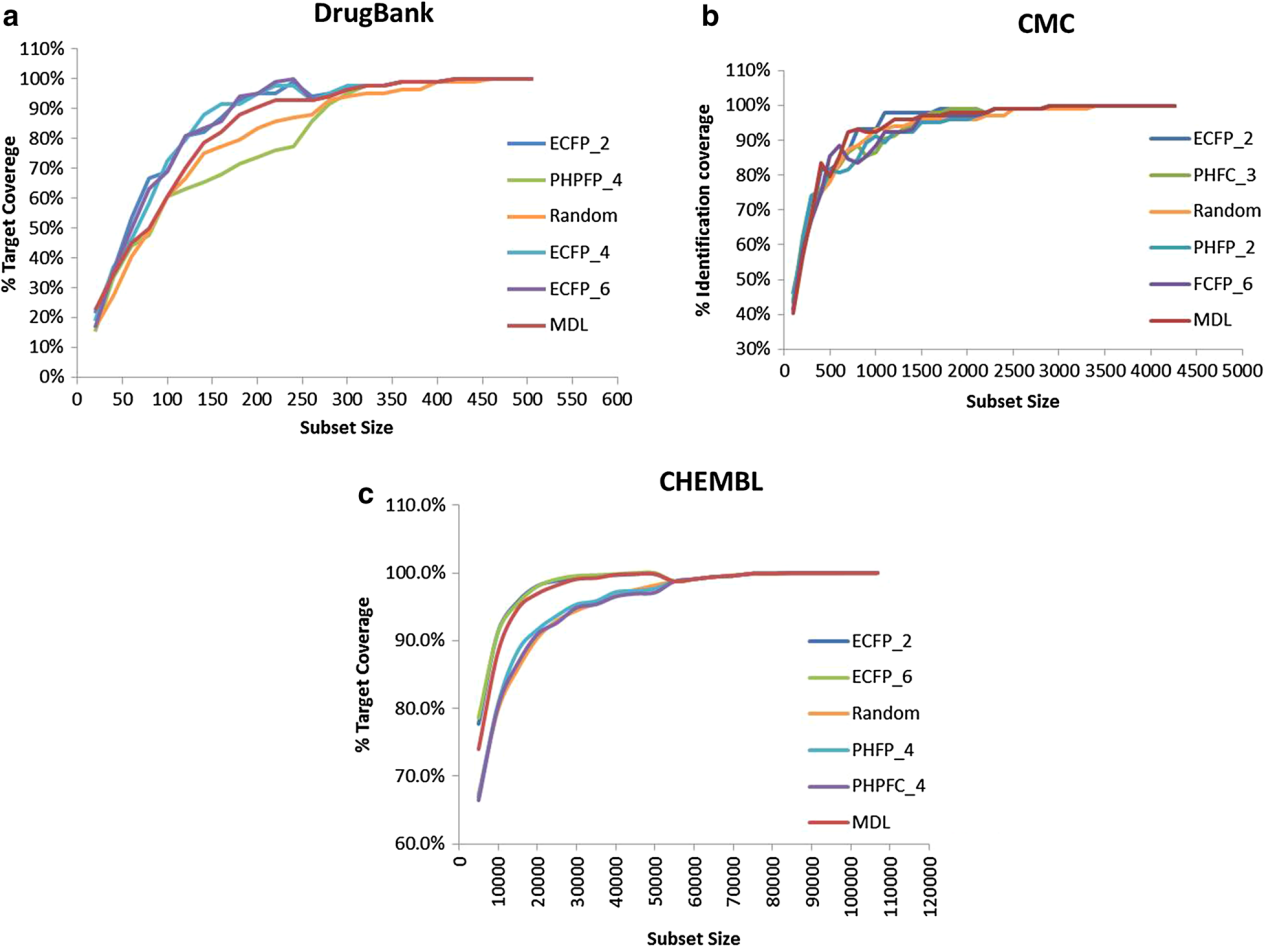
Assessment of “diversity descriptors”. Each graph presents the % of target (Drug Bank, CHEMBL) or indications (CMC) coverage as a function of the subset size for five of the 25 fingerprints evaluated in this work (see Additional file 1: Tables S1–S3 for details for all fingerprints). The five plots in each graph correspond to fingerprints covering the entire ranking range. **a** Selections made from Drug Bank. **b** Selections made from CMC. **c** Selections made from CHEMBL

Results obtained from the diversity analysis are presented in Fig. 2a–c and in Additional file 1: Tables S1–S3 for subsets selected from Drug Bank, CMC, and CHEMBL, respectively. The results demonstrate that: (1) in all three cases, performance differences between the different fingerprints are mostly apparent for intermediate subset sizes and (2) for the three databases, the ECFP_2 fingerprint performed best in terms of its ability to select small subsets which cover large parts of the targets/indications space. The performances of ECFP_2 were closely followed by those of ECFP_4 and ECFP_6. Additional fingerprints (e.g., MDL keys, and the 2D pharmacophoric fingerprints PHRFC_2) also performed well while the poorest results were obtained with some of the other 2D pharmacophoric fingerprints (e.g. PHPFP_4 and PHPFC_4) and as expected with random selection. Due to the high similarity between ECFP_2, ECFP_4, and ECFP_6 we chose to incorporate into the workflow three well-performing yet more diverse fingerprints (ECFP_2, MDL keys, and PHRFC_2) and evaluate library diversity using a consensus approach.

### Selection of similarity descriptors

**Fig. 3:**
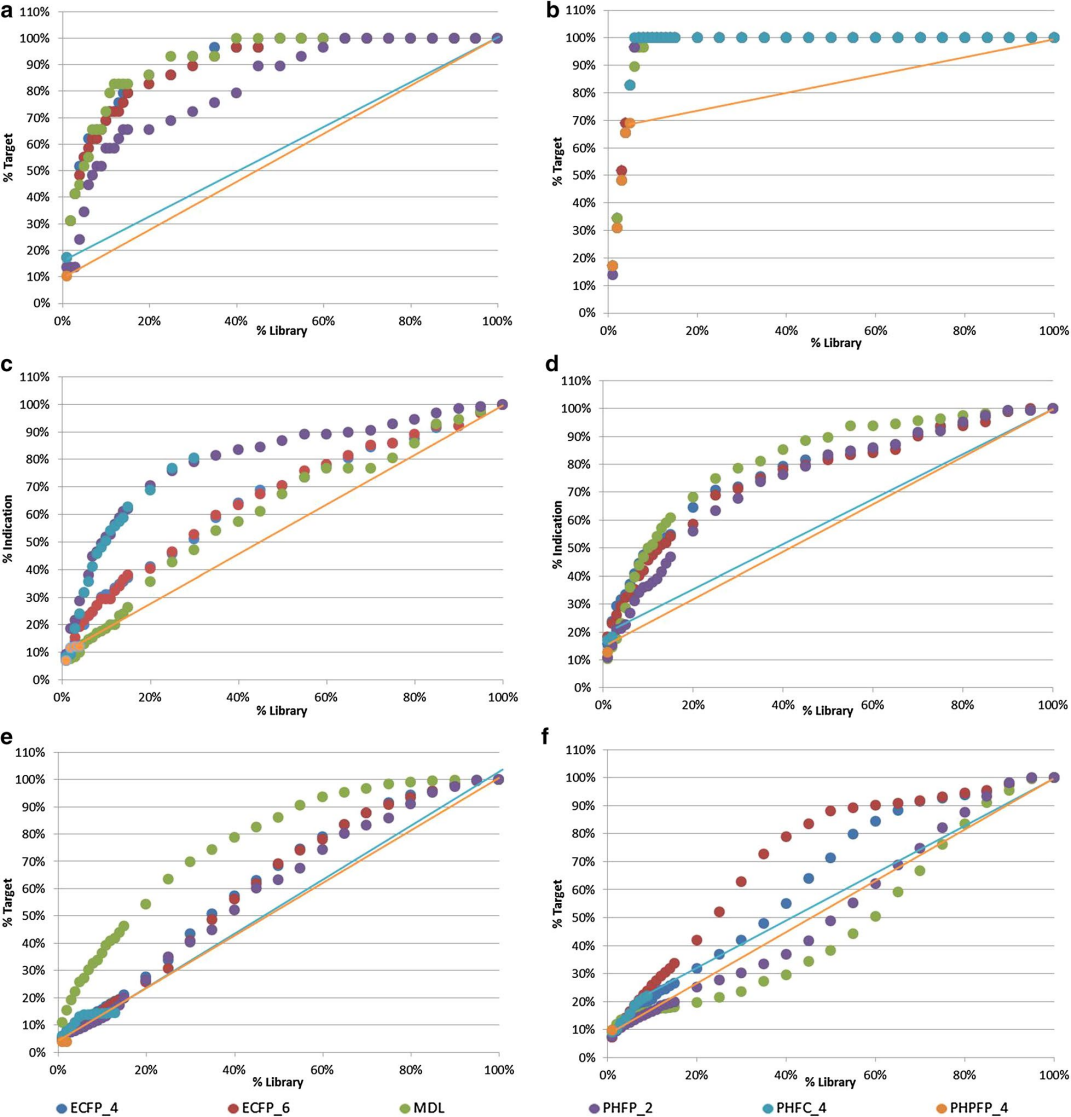
Enrichment curves obtained from the similarity analysis for six of the 25 fingerprints considered in this work (see Additional file 1: Tables S4–S9 for details on all fingerprints). **a**–**f** refer, respectively to Carbinoxamine, a ligand of the Histamine H1 receptor (CMC), fluocinolone acetonide, a ligand of the glucocorticiod receptor (CMC), lymecycline, an antibiotic drug (Drug Bank), haloperidol, an antipsychotic drug (Drug Bank), CHEMBL488890, a ligand of the Melanin-concentrating hormone receptor 1 and CHEMBL14759 a ligand of the human immunodeficiency virus type 1 protease as the reference (known active) compounds. For fingerprints in which the Tanimoto coefficient levels off before covering 100 % of the target there is no way to differentiate the compounds from one another hence these fingerprints are displayed as *straight lines* from the point where the coefficients levels off

Enrichment curves obtained for the similarity analysis are presented in Fig. 3a–f for active compounds selected from the Drug Bank, CMC and CHEMBL, respectively. Based on this analysis four fingerprints, ECFP_4, ECFP_6, MDL, and PHFP_3, were identified as best for similarity selection and incorporated into the workflow. Interestingly two of these fingerprints (ECFP_4, ECFP_6) were identified as best for the diversity analysis as well. Other non-pharmacophoric fingerprints (ECFP, FCFP and MDL) also performed well while four out of six 4-point pharmacophoric fingerprints (PHPFP_4, PHRFP_4, PHPFC_4 and PHRFC_4) were found to be the least successful.

### Application to external libraries

The resulting workflow was used to rank nine screening libraries obtained from six known vendors with the aim of selecting the best library under different scenarios as listed below. These input libraries are listed in Table 1 together with average values of several key descriptors.

**Table 1:** List of evaluated libraries

Vendor	Library	Size	Avg. MW	Avg. AlogP	Avg. H-bond acceptors	Avg. H-bond donors	Avg. rotatable bonds
ASINEX	Elite libraries	70,114	385.01	2.19	4.22	1.09	4.54
ASINEX	Platinum collection	113,962	425.81	3.50	4.72	1.32	6.74
Chembridge	DIVERSet™-CL	50,000	347.71	1.81	3.60	1.21	4.96
Chembridge	DIVERSet™-EXP	50,000	321.82	2.64	3.35	1.02	4.10
Enamine	Drug-like set	20,160	345.07	2.62	3.94	0.93	4.68
Enamine	Pharmacological diversity set	10,240	360.80	3.02	3.92	1.02	5.22
Maybridge	Screening collection	54,318	332.64	3.30	3.73	0.96	4.38
Prestwick	Prestwick Chemical Library^®^	1280	344.16	1.43	4.36	1.83	5.07
Sigma	MSII full library	10,000	312.98	2.51	3.54	1.16	3.90

All nine libraries were downloaded as SDF files and were subjected to the complete workflow. The results are presented in Tables 2, 3, 4, 5, 6, 7, 8, 9 and 10.

#### Data curation

The results of data curation are presented in Table 2 and demonstrate that all libraries evaluated in this work are of high quality and only negligible compounds fractions (0–3 %) were filtered by the data curation step

**Table 2:** Library quality analysis

Library	In-organic (%)	Duplicates (%)	Mixtures (%)	Bad valance (%)	Total removed (%)^a^
Elite libraries	0.0	0.0	0.0	0.0	0.0
Platinum collection	0.0	0.0	0.0	0.0	0.0
DIVERSet™-CL	0.0	0.0	0.0	0.0	0.0
DIVERSet™-EXP	0.0	0.0	0.2	0.0	0.2
Drug-like set	0.0	0.0	1.1	0.0	1.1
Pharmacological diversity set	0.0	0.0	0.9	0.0	0.9
Maybridge screening collection	0.0	0.1	0.1	0.0	0.0
Prestwick Chemical Library^®^	0.6	0.1	2.4	0.0	3.1
MSII full library	0.0	0.0	0.2	0.0	0.2

^a^The percent of molecules removed as a result of data curation

#### ADME/T profiling

ADME/T profiling results are presented in Table 3. Overall, most compounds obey Lipinski’s and Veber’s rules yet for several libraries the percentage of compounds violating these rules is not negligible (Platinum Collection 6.4, 7.4 %, respectively, and Prestwick Chemical Library^®^ 7.9, 13.5 %, respectively). Between 39 and 66 % of compounds are predicted not to cross the blood brain barrier with the Maybridge screening collection presenting the largest percentage of compounds predicted to be BBB permeating (60.7 %). This is closely followed by compounds from the pharmacological diversity set and the DIVERSet™-EXP. Not unexpectedly, there is no clear correlation between the percent of molecules which fail the Lipinski/Veber filters and those which are predicted to be BBB impermeable.

**Table 3:** ADME/T profiling

Library	Fail Lipinski (%)	Fail Veber (%)	LogBB < 0 (%)
Elite libraries	0.0	0.0	65.7
Platinum collection	6.4	7.4	48.0
DIVERSet™-CL	0.0	0.1	61.7
DIVERSet™-EXP	0.0	0.0	41.7
Drug-like set	0.0	0.2	50.4
Pharmacological diversity set	0.0	0.4	42.4
Maybridge screening collection	2.3	1.5	39.3
Prestwick Chemical Library^®^	7.9	13.5	64.7
MSII full library	0.2	0.8	49.1

#### Promiscuous binders

Some of the libraries have non-negligible fraction of their compounds classified as promiscuous binders based on HTS and PAINS filtration (12 % for Prestwick Chemical Library^®^ and 5 % for the Sigma and Maybridge Screening Collections). This number is negligible for all other libraries (see Table 4).

**Table 4:** Promiscuous binders

Library	Promiscuous binders (%)
Elite libraries	1.7
Platinum collection	0.9
DIVERSet™-CL	2.7
DIVERSet™-EXP	2.5
Drug-like set	2.9
Pharmacological diversity set	1.8
Maybridge screening collection	4.6
Prestwick Chemical Library^®^	11.8
MSII full library	5.0

#### Internal diversity

Table 5 presents the averaged pairwise Tanimoto coefficients calculated for the three selected diversity descriptors for all libraries considered in this work. Lower numbers (i.e., lower similarities) correspond to higher internal diversities. Based on this analysis the Prestwick Chemical Library^®^ is the most internally diverse while the Elite Library is the least diverse.

**Table 5:** Internal diversity

Library	Mean similarity		
	ECFP_2	MDL	PHRFC_2
Elite libraries	0.229	0.561	0.068
Platinum collection	0.216	0.519	0.098
DIVERSet™-CL	0.199	0.515	0.053
DIVERSet™-EXP	0.199	0.405	0.074
Drug-like set	0.163	0.415	0.067
Pharmacological diversity set	0.208	0.447	0.084
Maybridge screening collection	0.168	0.355	0.073
Prestwick Chemical Library^®^	0.138	0.346	0.036
MSII Full library	0.157	0.107	0.069

Table 6 ranks the different libraries based on the ADME/T, promiscuity and internal diversity criteria as well as based on their consensus. Clearly different libraries rank differently when evaluated according to different criteria. The data in Table 6 indicate that: (1) based on ADME/T profiling the best screening library is DIVERSet™-EXP, (2) based on the promiscuity criterion the best screening library is the Platinum collection, (3) based on internal diversity the best screening library is Prestwick Chemical Library^®^ and (4) when considering all three criteria with equal weights the best screening library is DIVERSet™-EXP dataset. Several libraries (e.g., DIVERSet™-EXP and Platinum collection) having equal ranks. Selecting between these equally ranked libraries will therefore require additional considerations, e.g., price or time to delivery.

**Table 6:** Library ranking based on ADME/T, promiscuous binders, diversity, and the consensus of the three

Library	ADME/T^b^	Promiscuous binders^b^	Diversity^b^	Total rank^a^
Elite libraries	4	2	7	13 (4)
Platinum collection	6	1	8	15 (5)
DIVERSet™-CL	3	5	7	15 (5)
DIVERSet™-EXP	1	4	5	10 (1)
Drug-like set	3	6	3	12 (3)
Pharmacological diversity set	2	3	6	11 (2)
Maybridge screening collection	3	7	3	13 (4)
Prestwick Chemical Library^®^	7	9	1	17 (6)
MSII full library	5	8	2	15 (5)

^a^The “Total Rank” column provides the sum of the “ADME/T”, promiscuity and “Diversity” columns and in parenthesis, the final rank of each library

^b^The ADME/T, promiscuous binders and diversity ranking were obtained from Tables 3, 4 and 5, respectively, using the same consensus approach as described in the text. In the case of ADME/T, consensus was taken over the Lipinski, Veber and logBB criteria. In the case of diversity, consensus was taken over the three selected diversity descriptors (ECFP_2, MDL, PHRFC_2)

#### Similarity to known active compounds

Thus far, the analysis was only based on the characteristics of library compounds and is therefore suitable for selecting screening libraries for unbiased screening. However, when additional information is available, e.g., knowledge of active compounds, it can be used to favorably bias the selection.

**Fig. 4:**
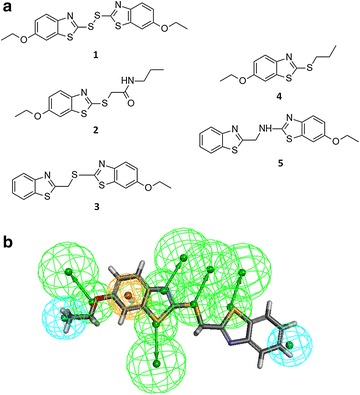
**a** Active (**1–3**) and inactive (**4–5**) benzothiazole derivatives used to build and validate a pharmacophore model. **b** Active compound **3** fitted to the pharmacophore model. *Green*, *cyan*, and *orange* colors represent H-bond acceptor, hydrophobic and ring-aromatic features, respectively

**Fig. 5:**
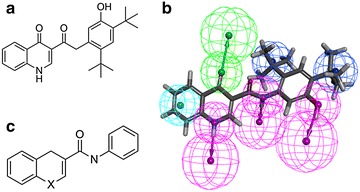
**a** Kalydeco structure. **b** Kalydeco fitted to its pharmacophore model. *Purple*, *green*, *cyan*, and *blue* colors represent H-bond donor, H-bond acceptor, hydrophobic-aromatic and hydrophobic-aliphatic features, respectively. **c** Kalydeco’s substructure used for substructure-based similarity analysis, where *x* represents one of the following atoms: nitrogen, oxygen, sulfur, aliphatic carbon or aromatic carbon

With this in mind, each library was evaluated with respect to its similarity to known (arbitrary selected) active compound(s). For this purpose we selected two sets of compounds: (1) a set of benzothiazole derivatives with known anti-hyperglycemic activity previously identified by us (Fig. 4a) [34]. These compounds were selected to exemplify the usage of a rigorously built and validated pharmacophore model in the selection procedure. These compounds were therefore only used for pharmacophore-based similarity (Fig. 4b) and were not used for library ranking; (2) Vertex’s Kalydeco which was recently approved by the FDA for the treatment of Cystic Fibrosis [35] (Fig. 5a). This compound was used as a proof of concept to exemplify the usage of a single compound-based pharmacophore. In order to analyze the similarity of the libraries to this compound all three approaches were used (pharmacophore-based (Fig. 5b), fingerprints-based using the best similarity descriptors, ECFP_4, ECFP_6, MDL and PHFP_3 fingerprints and substructure-based (Fig. 5c).

**Table 7:** Number of compounds found to be similar to a known active compound(s)

Library^a^	Benzothiazole derivatives		Kalydeco						
	Pharmacophore-based^b^		Pharmacophore-based^b^		Substructure-based	Fingerprint-based			
	All features	Omitting three	All features	Omitting two		ECFP_4	ECFP_6	MDL	PHFP_3
Elite libraries (70,111)	3	5419	2	2402	0	0	0	200	0
Platinum collection (113,961)	414	4942	0	1685	7	0	0	2076	0
DIVERSet™-CL (50,000)	22	32,547	19	8719	0	0	0	125	0
DIVERSet™-EXP (49,888)	61	30,203	4	8014	30	0	0	530	0
Drug-Like Set (19,932)	116	15,067	4	3687	6	0	0	82	0
Pharmacological diversity set (10,144)	151	8250	4	2687	5	0	0	81	0
Maybridge screening collection (54,174)	112	10,110	2	2530	3	1	0	514	0
Prestwick Chemical Library^®^ (1240)	14	663	1	249	0	0	0	11	0
MSII full library (9980)	30	5822	0	1533	1	0	0	291	0

^a^In brackets is the size of the library following data curation

^b^For pharmacophore-based similarity, two fitting procedures were performed either requiring a fit of all pharmacophoric features or allowing the omission of three/two features from the benzothiazole derivatives and Kalydeco pharmacophore models, respectively

**Fig. 6:**
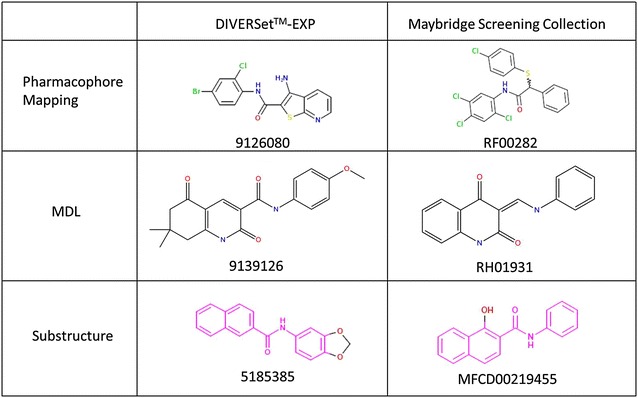
Compounds from the DIVERSet™-EXP and the Maybridge screening collection libraries which were identified as similar to the active compound Kalydeco using the three similarity methods, pharmacophore-based, fingerprint-based and substructure-based

The results of these analyses are presented in Table 7. The library with the largest number of compounds completely (i.e., with no omission of pharmacophoric features) matching the benzothiazole derived pharmacophore is the Platinum collection. All libraries (except Elite Libraries) feature more than ten pharmacophore-matching compounds. This is especially interesting in light of the large number of features included in this model (eight features). Not surprisingly, the number of matches increased when allowing for the omission of three features. In the case of Kalydeco, different similarity metrics led to different results with DIVERSet™-CL, Platinum collection, and DIVERSet™-EXP providing the largest number of matches using pharmacophore (either with or without feature omission), fingerprint or substructure-based similarity, respectively. For all libraries, pharmacophore-based similarity with the omission of two features yielded the largest number of similar compounds yet, these numbers drastically decreased when requiring complete matching, probably due to the large number of feature in this pharmacophore model (seven features). Figure 6 presents some of the best matches to Kalydeco obtained from the different libraries.

**Table 8:** Library ranking based on similarity to Kalydeco

Library	Substructure-based	Pharmacophore-based	Fingerprint-based	Total rank^a^
Elite libraries	7	6	4	17 (6)
Platinum collection	2	7	1	10 (2)
DIVERSet™-CL	7	1	5	13 (4)
DIVERSet™-EXP	1	2	2	5 (1)
Drug-like set	3	3	6	12 (3)
Pharmacological diversity set	4	4	7	15 (5)
Maybridge screening collection	5	5	2	12 (3)
Prestwick Chemical Library^®^	7	8	8	23 (7)
MSII full library	6	8	3	17 (6)

^a^The “Total rank” column provides the sum of all rankings and in parenthesis, the final rank of each library

**Table 9:** Ranking based on ADME/T, promiscuous binders, diversity and similarity to Kalydeco

Library	ADME/T ranking	Promiscuous binders	Diversity ranking	Similarity ranking	Total rank^a^
Elite libraries	4	2	7	6	19 (5)
Platinum collection	6	1	8	2	17 (4)
DIVERSet™-CL	3	5	7	4	19 (5)
DIVERSet™-EXP	1	4	5	1	11 (1)
Drug-like set	3	6	3	3	15 (2)
Pharmacological diversity set	2	3	6	5	16 (3)
Maybridge screening collection	3	7	3	3	16 (3)
Prestwick Chemical Library^®^	7	9	1	7	24 (7)
MSII full library	5	8	2	6	21 (6)

^a^The “Total rank” column provides the sum of all rankings and in parenthesis, the final rank of each library

Results obtained with each similarity method could be individually used as selection criteria. Alternatively, a consensus approach could be used (see Table 8). Based on this consensus, the library with the largest number of compounds similar to Kalydeco is DIVERSet™-EXP.

The ranking of the libraries based on molecular similarity can be combined with the results presented in Table 6 into a single consensus score. The new rank is provided in Table 9 and demonstrates that upon introducing the similarity to a known active drug criteria (Kalydeco), the highest ranking library is now DIVERSet™-EXP.

Finally all libraries were compared to the DIVERSet™-CL library (arbitrarily selected to represent an in house compound collection) using ECFP_4 which was previously identified as the best similarity fingerprint and a 0.7 Tanimoto coefficient cutoff. The results of this analysis are presented in Table 10 and indicate that none of the other libraries contain a large number of compounds similar to those in DIVERSet™-CL. Nevertheless, these numbers were used to rank the different libraries in terms to their “overlap” with DIVERSet™-CL (lower overlapping libraries allocated higher ranks) and this ranking was combined with the previous rankings into a single consensus score for library selection. The new ranking of the eight libraries is presented in the last column of Table 10 and identifies the DIVERSet™-EXP as the best library.

**Table 10:** Similarity to ‘In-House’ library and final rankings

Library	Similar molecules (%)	Library comparison ranking	Total rank^a^
Elite libraries	0.1	2	7 (4)
Platinum collection	0.0	1	5 (3)
DIVERSet™-EXP	0.1	2	3 (1)
Drug-like set	0.1	2	4 (2)
Pharmacological diversity set	0.0	1	4 (2)
Maybridge screening collection	0.1	2	5 (3)
Prestwick Chemical Library^®^	0.0	1	8 (5)
MSII full library	0.1	2	8 (5)

^a^The “Total rank” column provides the sum of all rankings, namely, ADME/T profiling, promiscuous binders, internal diversity, similarity to an active compound (Kalydeco) and similarity to an in-house reference library (DIVERSet™-CL)

## Discussion

This work presents a new workflow for the rational selection of libraries for biological screening. The development of this workflow was motivated by three main factors: (1) the screening of compound collections is a widely used starting point in many drug discovery projects. (2) Multiple compound collections from different vendors are currently available presenting practitioners in the field with the challenge of how to select the best one for a given project. This is particularly true when one wishes to select a complete library rather than to cherry pick a set of compounds from multiple libraries. (3) A unified workflow for the ranking of multiple candidate screening libraries based on multiple criteria is unavailable.

The main focus of the workflow is the selection of complete libraries for unbiased screening, namely screening which is not biased by knowledge of the structure of the biological target or of compounds known to interact with it. This screening often uses a phenotypic biological end point. This scenario is typical in projects where this knowledge is lacking (i.e., projects targeting new biological targets or new indications) or in projects where previous efforts utilizing this knowledge did not lead to active compounds. Nevertheless, in its current implementation the workflow can take advantage of known active compounds and use them as part of the library selection scheme.

The workflow accepts as input virtual representations of a set of screening libraries, typically as SD files. Such files could be downloaded from the sites of most vendors. In the present work we have retrieved, analyzed and ranked for selection nine screening libraries from six different vendors for a total of ~380,000 compounds (see Table 1). Data curation suggests that all libraries are well curated with only a small number of duplicates (Table 2). These usually result from different salts which were stripped as part of the data curation process. Similarly, all libraries have overall a favorable ADME/T profiles as reflected by adherence to Lipinski’s and Veber’s rules and lack of promiscuous binders and frequent HTS hitters. These observations are in accord with the tendency of vendors to produce drug-like libraries. Interestingly, the Prestwick Chemical Library^®^ which consists of FDA approved drugs has the largest fraction of compounds failing Lipinski’s and Veber’s rules (7.9 and 13.5 %, respectively). These findings are in line with the notion that not all drugs obey the Lipinski/Veber rules [36, 37].

ADME/T profiling was augmented by blood brain permeation predictions. For this purpose we developed and validated a logBB QSAR model using the genetic function approximation algorithm. The performances of the model are in line with previously reported logBB models [21, 38, 39]. For the purpose of evaluating screening libraries the quantitative model predictions were converted into permeable/impermeable categories leading to a success rate of ~80 %. We view this categorization as viable since at this stage we are only interested in the percentage of library compounds predicted to be BBB permeable/impermeable and not in the precise logBB values. Based on this model, between 34 and 61 % of libraries compounds are predicted to be blood brain barrier permeating with the Maybridge screening collection presenting the largest proportion (61 %). Thus, this library may be the most appropriate for the discovery of drugs targeting the central nervous system [40].

The internal diversity of compounds collections is especially important in unbiased, phenotypic screening since it increases the probability of identifying active hits. Several studies for evaluating diversity descriptors have been reported in the past [11, 33, 41] and it was not our intention to repeat them. Rather, we wished to identify, from within the set of fingerprints available to us those that not only perform best in target space but also could be used to identify diverse compounds in indication space. Again this is highly relevant for screening where the biological end point is based on phenotypic changes rather than on binding to a specific target. The best results were obtained by the ECFP_2, ECFP_4, and ECFP_6 fingerprints. These descriptors are highly related and only differ in the maximal distance (in terms of bond lengths) used for their derivation (two, four, and six for ECFP_2, ECFP_4, and ECFP_6, respectively). Thus ECFP_6 contains all features within ECFP_4 and ECFP_2. Still, we find it interesting, that the smallest fingerprint (ECFP_2) performed as well as (and marginally better) than the larger fingerprints. The ECFP results are expected to be library dependent since for libraries that contain a lot of analogues the smaller fingerprints (i.e., ECFP_2) would show more similarity compared to the larger fingerprint (i.e., ECFP_6) which would also consider the “decorations” on the scaffold.

Similarity to active compounds (if such are known) could be used to bias the selection of the screening library. The amount of biasing is user defined and depends on the number of active compounds used for searching the libraries and on the similarity threshold. Introducing some biasing into the library selection process does not interfere with the main purpose of this workflow, namely, the selection of libraries for phenotypic screening but rather increases the probability of identifying active hits. As for diversity, our purpose was not to challenge the literature consensus on the performance of fingerprint in similarity searching [42]. Rather our findings that the ECFP_4 fingerprint performed best also in the limited similarity searches we have performed in this work strengthen this consensus and extends it to the field of biological indications. Other descriptors that performed well for similarity searches are ECFP_6, MDL, and PHFP_3.

In this work similarity to known active compounds was assessed by three metrics, namely, fingerprint-bases (as discussed above), pharmacophore-based and substructure-based. In the case of Kalydeco, the usage of multiple similarity metrics allowed for some instructive comparisons. While most of the best similarity descriptors (ECFP_4, ECFP_6 and PHFP_3) provided only few matches to Kalydeco, MDL fingerprints provided multiple matches from most libraries. With respect to pharmacophore-based similarity, only few matches were identified using the pharmacophore model with complete fitting. This result is not surprising due to the large number of pharmacophoric features in this model (seven). Feature-rich pharmacophores are useful for identifying compounds with high selectivity profiles. Allowing for the omission of two pharmacophoric features greatly increased the number of matches and in fact this measure provided the largest number of compounds similar to Kalydeco. Pharmacophore-based similarity can identify hits with structural diversity larger than those identified with substructure-based or fingerprint-based similarity searches. Finally, substructure-based similarity identified slightly larger numbers of matches than those identified by the complete matching pharmacophore model.

Overall we found no correlation between the library size and the number of similar compounds obtained from it. We further found no correlation between the number of compounds obtained using different similarity metrics. This last observation supports our decision to rank libraries based on their similarity to known active compounds using a consensus approach.

When in-house compound collections are available, they could also be used to bias library selection via two opposite strategies. (1) Select a library which is the most dissimilar from the in-house library in order to avoid compound redundancy and to improve coverage of chemistry space. (2) Select a library which is the most similar to the in-house library if the latter has favorable properties. In this work we found no overlap between the DIVERSet™-CL library (arbitrarily selected to represent an in-house compound collection) and any of the other libraries considered in this work. This finding however is largely dependent on the reference library.

One criterion which was not used for library ranking is library size. This is because high throughput screening is to a large extent “a game of numbers”. Resources affording, it is better to screen more compounds. This however, should be balanced against the additional resources required. Of note is that despite its size the Platinum collection did not come up as the highest ranking library under any of the scenarios except while considering the promiscuous binders ranking only.

Ranking of the nine libraries considered in this study in terms of the above-discussed criteria is provided in Tables 6, 8, 9, and 10. This information by itself may be useful for researchers interested in library screening. ADME/T profiling, compound promiscuity and internal diversity are the only metrics that do not depend on arbitrary choices made in this work (i.e., using Kalydeco as a reference active compound and DIVERSet™-CL as a reference in-house library). Based on these criteria, the best library is DIVERSet™-EXP Set which is closely followed by Enamine’s Pharmacological diversity and Drug-like sets. When incorporating the similarity to a known active compound criterion the best library is Chembridge’s DIVERSet™-EXP followed by Asinex’s platinum collection and when adding the distance from an in-house compounds collection the best libraries is still Chembridge’s DIVERSet™-EXP followed by Enamine’s Drug-like set. Using the different criteria the top ranked library in all three cases was Chembridge’s DIVERSet™-EXP however the second best library varied. Changing the active compound or the in-house reference library will likely change the results.

An important feature of the current workflow is its flexibility which is manifested in multiple ways: (1) new components could be easily added. These could include new algorithms for the assessment of diversity and similarity, new tools to flag compounds with undesired properties and new QSAR models, either for activity prediction or for additional ADME/T profiling. (2) The ranking scheme could be easily modified either by including/omitting new criteria or by allocation different weights to different criteria. In this way the workflow can be easily tailored to select the best library under different scenarios.

The current workflow was implemented using the Pipeline Pilot software yet most of its components are available through different resources and consequently it could be implemented using alternative tools. In the following we provide a short, non-exhaustive list of options. Tools for data curation are available through the ChemAxon cheminformatics platform [43] which also allows for similarity searches. Pharmacophore models could be derived and used for database searching using Pharmer [44, 45] and PharmaGist [46, 47]. Additional similarity searches based on fingerprints, 2D pharmacophoric fingerprints and the Tanimoto coefficient could be performed with RDKit [48] or with the CDK toolkit [49]. Multiple descriptors could be calculated with the Dragon program [50] and QSAR algorithms could be derived with WEKA [51]. Finally, these tools could be combined into a single workflow using component integration platforms such as KNIME [52].

## Conclusions

We have devised a new workflow for the rational selection of screening libraries under different scenarios. This workflow has been routinely used in our laboratory for the selection of such libraries in multiple projects and consistently selects libraries which are well balanced across multiple parameters. We therefore expect this workflow to be useful for other laboratories engaged in drug discovery projects. The Pipeline Pilot workflow could be found in the supporting information. This workflow could be easily modified, e.g., by including additional components.

## Methodology

The library selection workflow was created using the Pipeline Pilot software version 8.5 [53] (although other tools could also be used; see discussion section). The Pipeline Pilot implementation is shown in Additional file 1: Figure S2 and the workflow is available in Additional file 2.

### Data curation

Data curation includes the removal of duplicates, inorganic molecules, mixtures, and salts, the standardization of chemical structures, the determination of protonation states at physiological pH (7.4) and the selection of the tautomeric state. In this workflow we selected only a single tautomer for each compound in order not to inflate the screening libraries. However multiple tautomers could be readily added.

### ADME/T profiling

#### Adherence to Lipinski’s and Veber’s Rules

Compounds violating more than one of Lipinski’s criteria (H-bond donors >5, Molecular weight >500, CLogP >5, H-bond acceptors >10) or any of Veber’s criteria (rotatable bonds ≤10, and polar surface area ≤140 Å^2^ or H-bond count ≤12) were flagged and their number was used as a criterion for library selection.

#### Development of a blood brain barrier permeation (logBB) QSAR Model

A quantitative logBB model was developed from a database of 152 compounds with known logBB values which was assembled from the literature [21, 38, 54]. 70 of these compounds are positively charged, 75 are neutral and 7 are negatively charged. 33 of these compounds were identified as outliers using a new, *k* nearest neighbor-based outlier removal algorithm and removed [55]. The remaining compounds were divided into training and test sets (80 and 39 compounds, respectively) [56]. Models were generated based on training set compounds using the Genetic Function Approximation (GFA) as implemented in the Discovery Studio [57] and the best model (based on the lack-of-fit criterion) was validated with the test set. To guard against chance correlation, Y-scrambling was performed by randomly shuffling the activities within the training set and repeating the model generation and validation steps. This procedure was repeated ten times.

The validated model was added to the library selection workflow and was used to predict the logBB values of molecules in the input libraries. Quantitative results were transformed into qualitative results by considering compounds with predicted logBB ≥ 0 as permeating and compounds with logBB < 0 as non-permeating. This information was used as another library selection criterion.

### Promiscuous Binders

The filtration of promiscuous binders was implemented by using the HTS filter available in Pipeline pilot and by complementing it with additional substructures based on the PAINS filter [23]. A list of all substructures used for filtration is given in Additional file 1: Table S10.

### Internal diversity

Internal diversity was evaluated using molecular fingerprints to characterize compounds and the Tanimoto coefficient to calculate pair-wise distances between them. Other similarity coefficients are available but the Tanimoto coefficient represent a well validated and commonly used option [58, 59]. The average distance over all pairs was taken to represent the library’s internal diversity. Prior to library evaluation, the best “diversity descriptors” were selected by evaluating the performances of different fingerprints in terms of their ability to select diverse subsets from within the Drug Bank [60], the Comprehensive Medicinal Chemistry (CMC) [61] and the CHEMBL databases. Diversity was evaluated by the number of different targets/indications covered by the selected subsets. This (indirect) diversity measure has its disadvantages as similar ligands can bind to different targets or alternatively, structurally diverse ligands may bind to the same target. However, we chose this method since this was highly applicable to the initial question addressed in this work, namely, the selection of compound libraries which would produce active hits upon screening. Other methods are available for evaluating diversity but each would have its own limitations and biases.

The Drug Bank database (downloaded on Nov. 2013) contains 721 compounds covering 215 different targets. In order to improve the compound-target ratio, targets with only one compound were removed from the database resulting in a dataset of 597 compounds covering 91 targets. Data curation and descriptors calculation led to the removal of five entries containing more than a single compound, five inorganic compounds, three duplicates, and 77 compounds for which descriptors could not be calculated (typically since these compounds did not include the required number of pharmacophoric features) and to a final dataset of 507 compounds covering 84 targets (see Additional file 1: Table S11).

The CMC database (downloaded on Nov. 2013) contains 9522 pharmaceutical compounds. These were classified into different indications by manually inspecting all database entries (we define indication as a certain symptom which could be treated by the compound rather than the binding of the compound to a certain target). Following the removal of compounds classified into more than one target, the removal of targets with only one or two compounds and data curation (removal of 81 entries with more than a single molecule, 128 inorganic molecules, 21 duplicates, and 594 compounds for which descriptors could not be calculated), the final dataset contained 4264 compounds covering 104 different biological indications (see Additional file 1: Table S12).

The June 2015 version of the CHEMBL database was downloaded and filtered to retain all compounds containing a benzene ring and with MW < 800 Dalton leading to a dataset with 1,098,971 compounds. This dataset was further processed first by removing compounds classified to more than a single target, then by removing targets with only one or two compounds and finally by subjecting it to data curation. This last stage led to the removal of 376 inorganic molecules, 145 duplicates, and 2408 compounds for which descriptors could not be calculated. The final dataset contained 106,860 compounds covering 1207 different targets (see Additional file 1: Table S13).

Distributions of key properties of the three filtered datasets (molecular weight, AlogP, number of rotatable bonds, number of H-bond donors and acceptors) are given in Additional file 1: Figures S3–S5.

The curated datasets were subsequently used for the selection of the “diversity descriptors”. For this, 26 subsets covering a range of 20–507 in 20 compounds intervals were selected from the Drug Bank dataset, 22 subsets covering a range of 5000–106,860 were selected for the CHEMBL dataset and 43 subsets covering a range of 100–4264 in 100 compounds intervals were selected from the CMC dataset, using 25 2D fingerprints (FCFP_2, FCFP_4, FCFP_6, ECFP_2, ECFP_4, ECFP_6, MDL Public keys, PHFP_2, PHFP_3, PHFP_4, PHPFP_2, PHPFP_3, PHPFP_4, PHRFP_2, PHRFP_3, PHRFP_4, PHFC_2, PHFC_3, PHFC_4, PHPFC_2, PHPFC_3, PHPFC_4, PHRFC_2, PHRFC_3, PHRFC_4 [62, 63], see additional information for a brief description of the fingerprints evaluated in this work) for a total of 650, 1075 and 550 subsets for Drug Bank, CMC and CHEMBL, respectively. Selections were made using the ‘Diverse Molecule’ component as implemented in Pipeline Pilot [53]. Each subset was checked for the number of different targets (Drug Bank, CHEMBL)/indications (CMC) it covered and this number was plotted as a function of subset size for the different fingerprints (Fig. 2). In this graph, higher Y values correspond to better “diversity fingerprints”. The best fingerprint was taken as that with the highest averaged targets/indications coverage across all subsets and across the three datasets. For comparison we evaluated the performances of random numbers by selecting subsets of similar sizes at random.

### Similarity to known active compounds

Similarity to known active compounds was evaluated using three different approaches, namely, pharmacophore-based, fingerprint-based, and substructure-based. In this work we arbitrarily selected as an active compound the Cystic Fibrosis (CF) drug Kalydeco (Fig. 5a) which was recently approved by the FDA for the treatment of CF patients having the G551D mutation [35]. For pharmacophore based similarity, we used as another example, a series of benzothiazole derivatives with known anti-hyperglycemic activity [34].

#### Pharmacophore-based similarity

Pharmacophore models were derived using the common feature pharmacophore generation procedure as implemented in the Discovery Studio software [57] using the following pharmacophoric features: H-bond donors and acceptors, positive and negative ionizable centers, aromatic rings and hydrophobic centers. Two strategies were considered: (1) building a pharmacophore from multiple active and inactive compounds. In the present study, this strategy was exemplified by using a pharmacophore previously developed by us from a series of benzothiazole derivatives with known anti-hyperglycemic activity. This pharmacophore was developed based on five compounds (three active and two inactive) and was shown to accurately distinguish between active and inactive compounds in an external test set consisting of 32 compounds [34]. (2) Building a pharmacophore based on a single active compound. In the present study this strategy was exemplified by building a pharmacophore from the cystic fibrosis (CF) drug Kalydeco (Fig. 5b). No information is available about the bioactive conformation of Kalydeco however due to its relative rigidity a reasonable pharmacophore model could nevertheless be proposed. We note that this pharmacophore was not validated and is presented only as a proof of concept. In both cases, mapping was performed twice either while allowing pharmacophoric features to be omitted (a maximum of two for the Kalydeco pharmacophore and three for the benzothiazole derivatives pharmacophore) or with no omissions allowed.

#### Fingerprint-based similarity

As in the case of diversity analysis, prior to library evaluation it was necessary to select the best “similarity descriptors”. This was performed by evaluating the performances of the same fingerprints in terms of their ability to identify from within the Drug Bank, CHEMBL and CMC databases active compounds based on their similarity to known reference (active) compounds. For this purpose, two compounds representing the two largest target classes in Drug Bank (Fluocinolone acetonide (DB00591) and Carbinoxamine (DB00748) belonging to the Glucocorticiod receptor and Histamine H1 receptor classes, respectively) were selected. Next, all Drug Bank compounds were ranked according to their similarity with respect to each reference compound using all 25 fingerprints and each ranked list was used for the calculation of an enrichment curve. A similar analysis was performed on the CMC database using Haloperidol (MCMC00000084), and Lymecycline (MCMC00001545) representing, respectively, the antipsychotic, and antibiotic indications and on the CHEMBL database using CHEMBL488890 and CHEMBL14759 representing, respectively, the Melanin-concentrating hormone receptor 1 and Human immunodeficiency virus type 1 protease. The best “similarity descriptors” were taken to be those producing the highest enrichment across all six reference compounds from the three databases. Highest enrichment was considered as the highest averaged active compounds coverage over the entire enrichment curve and across all six compounds (Fig. 3). These descriptors were introduced into the workflow and their usage was exemplified by searching the screening libraries for compounds similar to Kalydeco.

#### Substructure-based similarity

A substructure of Kalydeco (Fig. 5c) was generated from its structure by removing all substituents and by setting the heteroatom in the pyridinone ring to nitrogen, oxygen, sulfur, aliphatic carbon or aromatic carbon. This substructure was used for library screening.

### Similarity to an ‘In-House’ library

Library comparison was performed by identifying compounds in the new library which are similar to compounds in an ‘in-house’ library. In this study we arbitrarily selected DIVERSet™-CL as the in-house library. Similarity was evaluated by calculating Tanimoto coefficients using the best similarity fingerprints identified in Sect. “Selection of similarity descriptors” above. For each new library, the number of compounds similar (using a similarity threshold of 0.7) to compounds within the in-house library was calculated and this number was used as a criterion for library selection (lower numbers corresponded to a high library rank).

### Consensus ranking

Consensus scoring was implemented by first ranking each library according to each criterion so that the best library gets a score of 1 and the worst, a score of *x*, *x* being the number of libraries and then by combining the individual ranks. The library with the lowest combined rank (score) is taken to be the best one.

### Application to external libraries

Nine libraries from six different vendors were downloaded from the corresponding sites (see Table 1) and subjected to the workflow described above. These libraries were analyzed under different scenarios and the best library in each case was identified.

## Additional files

10.1186/s13321-015-0108-0 Brief description of the evaluated fingerprints. **Table S1.** Diversity analysis results for the Drug Bank database, **Table S2.** the CMC database and **Table S3.** the CHEMBL database, **Table S4.** similarity analysis results for the six reference active drugs Carbinoxamine, **Table S5.** Fluocinolone acetonide, **Table S6.** Lymecycline, **Table S7.** Haloperidol, **Table S8.** CHEMBL488890 and **Table S9.** CHEMBL14759. **Table S10.** Substructures for promiscuous binders and HTS screening. **Table S11.** Target classification in the Drug Bank database, **Table S12.** indication classification in the CMC database, **Table S13.** target classification in the CHEMBL database. **Figure S1.** Correlation between experimental and predicted logBB values, **Figure S2.** full description of the workflow as implemented in Pipeline Pilot, ** Figure S3.** distributions and statistical values of key properties of the Drug Bank, **Figure S4.** CMC and **Figure S5.** CHEMBL databases.
10.1186/s13321-015-0108-0 xml file of presented workflow.
