# Supplementary material for: A reliable computational workflow for the selection of optimal screening libraries
Source: J Cheminform. 2015 Dec 11;7:61. doi: 10.1186/s13321-015-0108-0 (PMC4676138; doi:10.1186/s13321-015-0108-0)
Supplement: Supplementary file 1 — 10.1186/s13321-015-0108-0 Brief description of the evaluated fingerprints. Table S1. Diversity analysis results for the Drug Bank database, Table S2. the CMC database and Table S3. the CHEMBL database, Table S4. similarity analysis results for the six reference active drugs Carbinoxamine, Table S5. Fluocinolone acetonide, Table S6. Lymecycline, Table S7. Haloperidol, Table S8. CHEMBL488890 and Table S9. CHEMBL14759. Table S10. Substructures for promiscuous binders and HTS screening. Table S11. Target classification in the Drug Bank database, Table S12. indication classification in the CMC database, Table S13. target classification in the CHEMBL database. Figure S1. Correlation between experimental and predicted logBB values, Figure S2. full description of the workflow as implemented in Pipeline Pilot, Figure S3. distributions and statistical values of key properties of the Drug Bank, Figure S4. CMC and Figure S5. CHEMBL databases. [file 13321_2015_108_MOESM1_ESM.docx]

**Supporting Information**

**A Reliable Computational Workflow for the Selection of Optimal Screening Libraries**

Yocheved Gilad,^1^ Katalin Nadassy^2^ and Hanoch Senderowitz^1^*

^1^Department of Chemistry, Bar-Ilan University, Ramat-Gan 52900 Israel

^2^Dassault Systèmes BIOVIA, 334 Cambridge Science Park, Cambridge CB4 0WN UK

* e-mail address: hsenderowitz@gmail.com

**Fingerprints Description**

Below we provide some information on the molecular fingerprints considered in this work. More information could be found at Accelrys help center and several published papers.[^1-3^](#_ENREF_1)

2D molecular structures are uniquely characterized in a binary format using fingerprints.

Several sets of fingerprints were used in this study, namely, ECFP, FCFP, PHFP, PHPFP, and PHRFP.

ECFP fingerprints are extended-connectivity fingerprints which use atom type, charge and hybridization category for atom classes. The fingerprints are presented as a list of features present in the molecule.

FCFP fingerprints are also extended-connectivity fingerprints. However they use functional classes rather than atom types. These classes include H-bond acceptor and donor, positive and negative ionizable centers, halogen, aromatic, and a class for features which are none of the above. As for ECFP the fingerprints are presented as a list of features.

Both sets include fingerprints with a different limitation on the maximum diameter (2, 4, or 6 bond lengths).

PHFP fingerprints are two dimensional fingerprints which include the number of bonds in the shortest path between molecular features. The features of interest could be pairs (PHFP_2), triplets (PHFP_3) or quartets (PHFP_4).

PHPFP fingerprints also include information regarding the types of bonds in the path between the features in addition to the number of bonds.

PHRFP fingerprints include information of the number of bonds as well as the number of rotatable bonds in the path between the features.

| **Table S1.** Diversity analysis results for the Drug Bank database | PHRFC_4 | 17 | 27 | 35 | 42 | 46 | 57 | 56 | 57 | 63 | 63 | 64 | 67 | 73 | 78 | 80 | 82 | 82 | 83 | 83 | 83 | 84 | 84 | 84 | 84 | 84 | 84 | This table presents the number of targets covered by each one of the different subset sizes using 25 fingerprints and a random selection. |
| --- | --- | --- | --- | --- | --- | --- | --- | --- | --- | --- | --- | --- | --- | --- | --- | --- | --- | --- | --- | --- | --- | --- | --- | --- | --- | --- | --- | --- |
|  | PHRFC_3 | 16 | 27 | 39 | 44 | 53 | 55 | 59 | 61 | 65 | 68 | 71 | 72 | 76 | 79 | 81 | 81 | 82 | 83 | 83 | 83 | 84 | 84 | 84 | 84 | 84 | 84 |  |
|  | PHRFC_2 | 17 | 33 | 46 | 50 | 55 | 55 | 64 | 72 | 75 | 76 | 80 | 82 | 77 | 79 | 81 | 82 | 82 | 83 | 83 | 83 | 84 | 84 | 84 | 84 | 84 | 84 |  |
|  | PHPFC_4 | 15 | 28 | 35 | 40 | 51 | 53 | 55 | 57 | 60 | 62 | 64 | 65 | 73 | 77 | 81 | 81 | 82 | 83 | 83 | 83 | 84 | 84 | 84 | 84 | 84 | 84 |  |
|  | PHPFC_3 | 15 | 29 | 33 | 42 | 47 | 52 | 57 | 60 | 63 | 65 | 67 | 70 | 77 | 80 | 82 | 82 | 82 | 83 | 83 | 83 | 84 | 84 | 84 | 84 | 84 | 84 |  |
|  | PHPFC_2 | 18 | 31 | 39 | 48 | 56 | 606 | 62 | 63 | 73 | 75 | 79 | 82 | 78 | 80 | 82 | 82 | 82 | 83 | 83 | 83 | 84 | 84 | 84 | 84 | 84 | 84 |  |
|  | PHFC_4 | 14 | 27 | 34 | 43 | 49 | 55 | 56 | 56 | 62 | 64 | 67 | 70 | 74 | 79 | 82 | 82 | 82 | 83 | 83 | 83 | 84 | 84 | 84 | 84 | 84 | 84 |  |
|  | PHFC_3 | 16 | 31 | 33 | 42 | 55 | 54 | 56 | 60 | 66 | 67 | 70 | 72 | 77 | 80 | 82 | 82 | 82 | 83 | 83 | 83 | 84 | 84 | 84 | 84 | 84 | 84 |  |
|  | PHFC_2 | 14 | 30 | 39 | 47 | 55 | 62 | 65 | 69 | 76 | 78 | 78 | 79 | 78 | 80 | 82 | 82 | 82 | 83 | 83 | 83 | 84 | 84 | 84 | 84 | 84 | 84 |  |
|  | PHRFP_4 | 17 | 27 | 35 | 42 | 45 | 57 | 56 | 57 | 63 | 63 | 64 | 67 | 72 | 78 | 80 | 82 | 82 | 83 | 83 | 83 | 84 | 84 | 84 | 84 | 84 | 84 |  |
|  | PHRFP_3 | 16 | 27 | 39 | 44 | 53 | 55 | 59 | 61 | 65 | 68 | 71 | 72 | 76 | 79 | 81 | 81 | 82 | 83 | 83 | 83 | 84 | 84 | 84 | 84 | 84 | 84 |  |
|  | PHRFP_2 | 19 | 30 | 42 | 50 | 5 | 57 | 65 | 68 | 73 | 75 | 79 | 81 | 77 | 79 | 81 | 82 | 82 | 83 | 83 | 83 | 84 | 84 | 84 | 84 | 84 | 84 |  |
|  | PHPFP_4 | 13 | 28 | 37 | 40 | 51 | 53 | 55 | 57 | 60 | 62 | 64 | 65 | 72 | 77 | 80 | 82 | 82 | 83 | 83 | 83 | 84 | 84 | 84 | 84 | 84 | 84 |  |
|  | Subset Size | 20 | 40 | 60 | 80 | 100 | 120 | 140 | 160 | 180 | 200 | 220 | 240 | 260 | 280 | 300 | 320 | 340 | 360 | 380 | 400 | 420 | 440 | 460 | 480 | 500 | 507 |  |
| **Table S1.** Diversity analysis results for the Drug Bank database Cont. | PHPFP_3 | 15 | 29 | 33 | 42 | 47 | 52 | 57 | 60 | 63 | 65 | 67 | 70 | 77 | 80 | 82 | 82 | 82 | 83 | 83 | 83 | 84 | 84 | 84 | 84 | 84 | 84 | This table presents the number of targets covered by each one of the different subset sizes using 25 fingerprints and a random selection. |
|  | PHPFP_2 | 16 | 32 | 40 | 42 | 53 | 58 | 61 | 65 | 74 | 77 | 79 | 81 | 78 | 80 | 82 | 82 | 82 | 83 | 83 | 83 | 84 | 84 | 84 | 84 | 84 | 84 |  |
|  | PHFP_4 | 15 | 26 | 34 | 43 | 49 | 55 | 56 | 56 | 62 | 64 | 67 | 70 | 73 | 78 | 80 | 82 | 82 | 83 | 83 | 83 | 84 | 84 | 84 | 84 | 84 | 84 |  |
|  | PHFP_3 | 15 | 29 | 33 | 42 | 5 | 54 | 49 | 63 | 64 | 66 | 70 | 74 | 77 | 80 | 82 | 82 | 82 | 83 | 83 | 83 | 84 | 84 | 84 | 84 | 84 | 84 |  |
|  | PHFP_2 | 15 | 30 | 39 | 47 | 52 | 57 | 58 | 69 | 75 | 75 | 78 | 79 | 78 | 80 | 82 | 82 | 82 | 83 | 83 | 83 | 84 | 84 | 84 | 84 | 84 | 84 |  |
|  | MDL | 19 | 29 | 38 | 42 | 51 | 59 | 66 | 69 | 74 | 76 | 78 | 78 | 78 | 79 | 81 | 82 | 82 | 83 | 83 | 83 | 84 | 84 | 84 | 84 | 84 | 84 |  |
|  | ECFP_6 | 14 | 30 | 42 | 53 | 58 | 68 | 70 | 72 | 79 | 80 | 83 | 84 | 78 | 79 | 81 | 82 | 82 | 83 | 83 | 83 | 84 | 84 | 84 | 84 | 84 | 84 |  |
|  | ECFP_4 | 16 | 31 | 39 | 49 | 61 | 67 | 74 | 77 | 77 | 80 | 82 | 82 | 78 | 80 | 82 | 82 | 82 | 83 | 83 | 83 | 84 | 84 | 84 | 84 | 84 | 84 |  |
|  | ECFP_2 | 18 | 30 | 45 | 56 | 58 | 68 | 69 | 73 | 78 | 80 | 80 | 83 | 79 | 80 | 82 | 82 | 82 | 83 | 83 | 83 | 84 | 84 | 84 | 84 | 84 | 84 |  |
|  | FCFP_6 | 16 | 30 | 42 | 50 | 59 | 68 | 70 | 75 | 77 | 78 | 80 | 82 | 79 | 80 | 82 | 82 | 82 | 83 | 83 | 83 | 84 | 84 | 84 | 84 | 84 | 84 |  |
|  | FCFP_4 | 16 | 26 | 41 | 53 | 62 | 70 | 70 | 72 | 78 | 79 | 80 | 81 | 79 | 80 | 82 | 82 | 82 | 83 | 83 | 83 | 84 | 84 | 84 | 84 | 84 | 84 |  |
|  | FCFP_2 | 16 | 27 | 46 | 49 | 57 | 65 | 63 | 68 | 80 | 81 | 82 | 82 | 78 | 80 | 82 | 82 | 82 | 83 | 83 | 83 | 84 | 84 | 84 | 84 | 84 | 84 |  |
|  | Random | 14 | 23 | 34 | 41 | 51 | 56 | 63 | 65 | 67 | 70 | 72 | 73 | 74 | 78 | 79 | 80 | 80 | 81 | 81 | 83 | 83 | 83 | 84 | 84 | 84 | 84 |  |
|  | Subset Size | 20 | 40 | 60 | 80 | 100 | 120 | 140 | 160 | 180 | 200 | 220 | 240 | 260 | 280 | 300 | 320 | 340 | 360 | 380 | 400 | 420 | 440 | 460 | 480 | 500 | 507 |  |

| **Table S2.** Diversity analysis results for the CMC database | PHRFC_4 | 45 | 63 | 69 | 78 | 81 | 81 | 90 | 94 | 94 | 95 | 95 | 96 | 96 | 96 | 101 | 102 | 102 | 102 | 102 | 102 | 102 | 102 | 103 | 103 | 103 | 103 | 103 | 103 | 104 | 104 | 104 | 104 | 104 | 104 | 104 | 104 | 104 | 104 | 104 | 104 | 104 | 104 | 104 | This table presents the number of indications covered by each one of the different subset sizes using 25 fingerprints and a random selection. |
| --- | --- | --- | --- | --- | --- | --- | --- | --- | --- | --- | --- | --- | --- | --- | --- | --- | --- | --- | --- | --- | --- | --- | --- | --- | --- | --- | --- | --- | --- | --- | --- | --- | --- | --- | --- | --- | --- | --- | --- | --- | --- | --- | --- | --- | --- |
|  | PHRFC_3 | 47 | 60 | 73 | 79 | 82 | 89 | 87 | 89 | 92 | 93 | 95 | 96 | 97 | 99 | 100 | 102 | 103 | 103 | 103 | 103 | 103 | 102 | 103 | 103 | 103 | 103 | 103 | 103 | 104 | 104 | 104 | 104 | 104 | 104 | 104 | 104 | 104 | 104 | 104 | 104 | 104 | 104 | 104 |  |
|  | PHRFC_2 | 50 | 60 | 67 | 77 | 84 | 93 | 93 | 94 | 94 | 94 | 100 | 100 | 100 | 101 | 100 | 101 | 101 | 101 | 102 | 102 | 102 | 102 | 103 | 103 | 103 | 103 | 103 | 103 | 104 | 104 | 104 | 104 | 104 | 104 | 104 | 104 | 104 | 104 | 104 | 104 | 104 | 104 | 104 |  |
|  | PHPFC_4 | 41 | 55 | 73 | 68 | 81 | 86 | 90 | 91 | 94 | 95 | 95 | 95 | 98 | 98 | 98 | 101 | 102 | 102 | 102 | 103 | 103 | 102 | 103 | 103 | 103 | 103 | 103 | 103 | 104 | 104 | 104 | 104 | 104 | 104 | 104 | 104 | 104 | 104 | 104 | 104 | 104 | 104 | 104 |  |
|  | PHPFC_3 | 45 | 62 | 72 | 81 | 85 | 83 | 87 | 90 | 93 | 95 | 98 | 99 | 101 | 101 | 98 | 101 | 101 | 101 | 101 | 101 | 101 | 102 | 103 | 103 | 103 | 103 | 103 | 103 | 104 | 104 | 104 | 104 | 104 | 104 | 104 | 104 | 104 | 104 | 104 | 104 | 104 | 104 | 104 |  |
|  | PHPFC_2 | 46 | 63 | 69 | 75 | 78 | 83 | 88 | 91 | 94 | 96 | 94 | 95 | 96 | 96 | 99 | 99 | 100 | 100 | 102 | 102 | 102 | 102 | 103 | 103 | 103 | 103 | 103 | 103 | 104 | 104 | 104 | 104 | 104 | 104 | 104 | 104 | 104 | 104 | 104 | 104 | 104 | 104 | 104 |  |
|  | PHFC_4 | 41 | 62 | 75 | 79 | 80 | 90 | 93 | 91 | 96 | 97 | 98 | 99 | 99 | 99 | 98 | 99 | 100 | 100 | 100 | 100 | 102 | 102 | 103 | 103 | 103 | 103 | 103 | 103 | 104 | 104 | 104 | 104 | 104 | 104 | 104 | 104 | 104 | 104 | 104 | 104 | 104 | 104 | 104 |  |
|  | PHFC_3 | 46 | 65 | 77 | 79 | 83 | 86 | 90 | 92 | 89 | 90 | 94 | 95 | 97 | 98 | 100 | 102 | 102 | 103 | 103 | 103 | 103 | 102 | 103 | 103 | 103 | 103 | 103 | 103 | 104 | 104 | 104 | 104 | 104 | 104 | 104 | 104 | 104 | 104 | 104 | 104 | 104 | 104 | 104 |  |
|  | PHFC_2 | 50 | 72 | 76 | 79 | 83 | 81 | 90 | 86 | 89 | 90 | 93 | 95 | 96 | 96 | 100 | 100 | 100 | 101 | 101 | 101 | 101 | 102 | 103 | 103 | 103 | 103 | 103 | 103 | 104 | 104 | 104 | 104 | 104 | 104 | 104 | 104 | 104 | 104 | 104 | 104 | 104 | 104 | 104 |  |
|  | PHRFP_4 | 42 | 59 | 72 | 81 | 81 | 82 | 91 | 94 | 94 | 95 | 95 | 96 | 96 | 96 | 101 | 102 | 102 | 102 | 102 | 102 | 102 | 102 | 103 | 103 | 103 | 103 | 103 | 103 | 104 | 104 | 104 | 104 | 104 | 104 | 104 | 104 | 104 | 104 | 104 | 104 | 104 | 104 | 104 |  |
|  | PHRFP_3 | 46 | 60 | 73 | 77 | 82 | 88 | 87 | 89 | 92 | 93 | 95 | 96 | 97 | 99 | 100 | 101 | 102 | 102 | 102 | 102 | 102 | 102 | 103 | 103 | 103 | 103 | 103 | 103 | 104 | 104 | 104 | 104 | 104 | 104 | 104 | 104 | 104 | 104 | 104 | 104 | 104 | 104 | 104 |  |
|  | PHRFP_2 | 43 | 60 | 69 | 82 | 86 | 89 | 93 | 90 | 94 | 95 | 97 | 100 | 100 | 101 | 101 | 101 | 101 | 101 | 101 | 101 | 101 | 102 | 103 | 103 | 103 | 103 | 103 | 103 | 104 | 104 | 104 | 104 | 104 | 104 | 104 | 104 | 104 | 104 | 104 | 104 | 104 | 104 | 104 |  |
|  | PHPFP_4 | 46 | 56 | 72 | 73 | 80 | 86 | 91 | 92 | 94 | 95 | 95 | 95 | 98 | 98 | 98 | 101 | 102 | 102 | 102 | 103 | 103 | 102 | 103 | 103 | 103 | 103 | 103 | 103 | 104 | 104 | 104 | 104 | 104 | 104 | 104 | 104 | 104 | 104 | 104 | 104 | 104 | 104 | 104 |  |
|  | Subset Size | 100 | 200 | 300 | 400 | 500 | 600 | 700 | 800 | 900 | 1000 | 1100 | 1200 | 1300 | 1400 | 1500 | 1600 | 1700 | 1800 | 1900 | 2000 | 2100 | 2200 | 2300 | 2400 | 2500 | 2600 | 2700 | 2800 | 2900 | 3000 | 3100 | 3200 | 3300 | 3400 | 3500 | 3600 | 3700 | 3800 | 3900 | 4000 | 4100 | 4200 | 4263 |  |
| **Table S2.** Diversity analysis results for the CMC database Cont. | PHPFP_3 | 45 | 62 | 72 | 80 | 85 | 83 | 87 | 90 | 93 | 95 | 98 | 99 | 101 | 101 | 98 | 101 | 101 | 101 | 101 | 101 | 101 | 102 | 103 | 103 | 103 | 103 | 103 | 103 | 104 | 104 | 104 | 104 | 104 | 104 | 104 | 104 | 104 | 104 | 104 | 104 | 104 | 104 | 104 | This table presents the number of indications covered by each one of the different subset sizes using 25 fingerprints and a random selection. |
|  | PHPFP_2 | 48 | 62 | 65 | 76 | 80 | 87 | 87 | 91 | 95 | 96 | 94 | 95 | 97 | 97 | 99 | 99 | 100 | 100 | 102 | 102 | 102 | 102 | 103 | 103 | 103 | 103 | 103 | 103 | 104 | 104 | 104 | 104 | 104 | 104 | 104 | 104 | 104 | 104 | 104 | 104 | 104 | 104 | 104 |  |
|  | PHFP_4 | 43 | 64 | 76 | 79 | 80 | 90 | 93 | 91 | 95 | 97 | 98 | 99 | 99 | 99 | 98 | 99 | 100 | 100 | 100 | 100 | 101 | 102 | 103 | 103 | 103 | 103 | 103 | 103 | 104 | 104 | 104 | 104 | 104 | 104 | 104 | 104 | 104 | 104 | 104 | 104 | 104 | 104 | 104 |  |
|  | PHFP_3 | 48 | 65 | 77 | 79 | 83 | 86 | 89 | 93 | 90 | 91 | 94 | 95 | 97 | 98 | 98 | 102 | 102 | 103 | 103 | 103 | 103 | 102 | 103 | 103 | 103 | 103 | 103 | 103 | 104 | 104 | 104 | 104 | 104 | 104 | 104 | 104 | 104 | 104 | 104 | 104 | 104 | 104 | 104 |  |
|  | PHFP_2 | 45 | 64 | 77 | 78 | 85 | 84 | 85 | 88 | 93 | 95 | 93 | 96 | 96 | 96 | 99 | 99 | 99 | 100 | 100 | 100 | 101 | 102 | 103 | 103 | 103 | 103 | 103 | 103 | 104 | 104 | 104 | 104 | 104 | 104 | 104 | 104 | 104 | 104 | 104 | 104 | 104 | 104 | 104 |  |
|  | MDL | 42 | 59 | 71 | 87 | 83 | 89 | 96 | 97 | 96 | 96 | 98 | 100 | 100 | 100 | 101 | 101 | 101 | 102 | 102 | 102 | 102 | 102 | 103 | 103 | 103 | 103 | 103 | 103 | 104 | 104 | 104 | 104 | 104 | 104 | 104 | 104 | 104 | 104 | 104 | 104 | 104 | 104 | 104 |  |
|  | ECFP_6 | 47 | 63 | 73 | 78 | 77 | 86 | 91 | 93 | 91 | 92 | 98 | 99 | 99 | 99 | 103 | 103 | 103 | 104 | 104 | 104 | 104 | 102 | 103 | 103 | 103 | 103 | 103 | 103 | 104 | 104 | 104 | 104 | 104 | 104 | 104 | 104 | 104 | 104 | 104 | 104 | 104 | 104 | 104 |  |
|  | ECFP_4 | 41 | 59 | 68 | 77 | 84 | 92 | 91 | 94 | 93 | 94 | 99 | 99 | 99 | 100 | 103 | 103 | 104 | 104 | 104 | 104 | 104 | 102 | 103 | 103 | 103 | 103 | 103 | 103 | 104 | 104 | 104 | 104 | 104 | 104 | 104 | 104 | 104 | 104 | 104 | 104 | 104 | 104 | 104 |  |
|  | ECFP_2 | 48 | 62 | 71 | 85 | 85 | 87 | 90 | 97 | 97 | 97 | 102 | 102 | 102 | 102 | 102 | 102 | 103 | 103 | 103 | 103 | 103 | 103 | 103 | 103 | 103 | 103 | 103 | 103 | 104 | 104 | 104 | 104 | 104 | 104 | 104 | 104 | 104 | 104 | 104 | 104 | 104 | 104 | 104 |  |
|  | FCFP_6 | 43 | 61 | 70 | 78 | 89 | 92 | 88 | 87 | 89 | 92 | 96 | 96 | 96 | 97 | 101 | 101 | 101 | 101 | 101 | 101 | 101 | 102 | 103 | 103 | 103 | 103 | 103 | 103 | 104 | 104 | 104 | 104 | 104 | 104 | 104 | 104 | 104 | 104 | 104 | 104 | 104 | 104 | 104 |  |
|  | FCFP_4 | 38 | 66 | 70 | 85 | 82 | 87 | 85 | 93 | 95 | 96 | 96 | 97 | 97 | 98 | 101 | 101 | 101 | 101 | 101 | 101 | 101 | 102 | 103 | 103 | 103 | 103 | 103 | 103 | 104 | 104 | 104 | 104 | 104 | 104 | 104 | 104 | 104 | 104 | 104 | 104 | 104 | 104 | 104 |  |
|  | FCFP_2 | 41 | 55 | 70 | 78 | 80 | 88 | 88 | 90 | 97 | 97 | 94 | 96 | 98 | 98 | 97 | 97 | 97 | 97 | 97 | 98 | 98 | 102 | 103 | 103 | 103 | 103 | 103 | 103 | 104 | 104 | 104 | 104 | 104 | 104 | 104 | 104 | 104 | 104 | 104 | 104 | 104 | 104 | 104 |  |
|  | Random | 43 | 62 | 72 | 78 | 81 | 87 | 91 | 92 | 94 | 97 | 97 | 98 | 98 | 99 | 100 | 100 | 100 | 100 | 100 | 100 | 100 | 101 | 101 | 101 | 103 | 103 | 103 | 103 | 103 | 103 | 103 | 103 | 103 | 104 | 104 | 104 | 104 | 104 | 104 | 104 | 104 | 104 | 104 |  |
|  | Subset Size | 100 | 200 | 300 | 400 | 500 | 600 | 700 | 800 | 900 | 1000 | 1100 | 1200 | 1300 | 1400 | 1500 | 1600 | 1700 | 1800 | 1900 | 2000 | 2100 | 2200 | 2300 | 2400 | 2500 | 2600 | 2700 | 2800 | 2900 | 3000 | 3100 | 3200 | 3300 | 3400 | 3500 | 3600 | 3700 | 3800 | 3900 | 4000 | 4100 | 4200 | 4263 |  |

| **Table S3.** Diversity analysis results for the CHEMBL database | PHRFC_4 | 792 | 972 | 1042 | 1094 | 1126 | 1149 | 1157 | 1168 | 1173 | 1174 | 1192 | 1196 | 1200 | 1202 | 1206 | 1206 | 1207 | 1207 | 1207 | 1207 | 1207 | 1207 | This table presents the number of targets covered by each one of the different subset sizes using 25 fingerprints and a random selection. |
| --- | --- | --- | --- | --- | --- | --- | --- | --- | --- | --- | --- | --- | --- | --- | --- | --- | --- | --- | --- | --- | --- | --- | --- | --- |
|  | PHRFC_3 | 819 | 1005 | 1084 | 1131 | 1159 | 1180 | 1196 | 1198 | 1199 | 1192 | 1196 | 1200 | 1202 | 1206 | 1206 | 1207 | 1207 | 1207 | 1207 | 1207 | 1207 | 1207 |  |
|  | PHRFC_2 | 858 | 1036 | 1123 | 1163 | 1186 | 1191 | 1194 | 1202 | 1205 | 1205 | 1192 | 1196 | 1200 | 1202 | 1206 | 1206 | 1207 | 1207 | 1207 | 1207 | 1207 | 1207 |  |
|  | PHPFC_4 | 802 | 970 | `046 | 1098 | 1118 | 1145 | 1151 | 1165 | 1170 | 1172 | 1192 | 1196 | 1200 | 1202 | 1206 | 1206 | 1207 | 1207 | 1207 | 1207 | 1207 | 1207 |  |
|  | PHPFC_3 | 813 | 971 | 1073 | 1110 | 1138 | 1165 | 1175 | 1197 | 1201 | 1203 | 1192 | 1196 | 1200 | 1202 | 1206 | 1206 | 1207 | 1207 | 1207 | 1207 | 1207 | 1207 |  |
|  | PHPFC_2 | 862 | 1048 | 1128 | 1157 | 1174 | 1188 | 1192 | 1202 | 1205 | 1205 | 1192 | 1196 | 1200 | 1202 | 1206 | 1206 | 1207 | 1207 | 1207 | 1207 | 1207 | 1207 |  |
|  | PHFC_4 | 799 | 971 | 1063 | 1107 | 1130 | 1150 | 1157 | 1174 | 1176 | 1180 | 1192 | 1196 | 1200 | 1202 | 1206 | 1206 | 1207 | 1207 | 1207 | 1207 | 1207 | 1207 |  |
|  | PHFC_3 | 838 | 1008 | 1095 | 1139 | 1163 | 1184 | 1191 | 1196 | 1198 | 1199 | 1192 | 1196 | 1200 | 1202 | 1206 | 1206 | 1207 | 1207 | 1207 | 1207 | 1207 | 1207 |  |
|  | PHFC_2 | 846 | 1023 | 1107 | 1139 | 1166 | 1193 | 1199 | 1198 | 1201 | 1201 | 1192 | 1196 | 1200 | 1202 | 1206 | 1206 | 1207 | 1207 | 1207 | 1207 | 1207 | 1207 |  |
|  | PHRFP_4 | 805 | 974 | 1042 | 1094 | 1126 | 1148 | 1157 | 1169 | 1173 | 1174 | 1192 | 1196 | 1200 | 1202 | 1206 | 1206 | 1207 | 1207 | 1207 | 1207 | 1207 | 1207 |  |
|  | PHRFP_3 | 823 | 1005 | 1084 | 1131 | 1159 | 1180 | 1187 | 1196 | 1198 | 1199 | 1192 | 1196 | 1200 | 1202 | 1206 | 1206 | 1207 | 1207 | 1207 | 1207 | 1207 | 1207 |  |
|  | PHRFP_2 | 858 | 1036 | 1123 | 1163 | 1186 | 1191 | 1194 | 1202 | 1205 | 1205 | 1192 | 1196 | 1200 | 1202 | 1206 | 1206 | 1207 | 1207 | 1207 | 1207 | 1207 | 1207 |  |
|  | PHPFP_4 | 811 | 969 | 1046 | 1098 | 1118 | 1145 | 1151 | 1165 | 1170 | 1172 | 1192 | 1196 | 1200 | 1202 | 1206 | 1206 | 1207 | 1207 | 1207 | 1207 | 1207 | 1207 |  |
|  | Subset Size | 5000 | 10000 | 15000 | 20000 | 25000 | 30000 | 35000 | 40000 | 45000 | 50000 | 55000 | 60000 | 65000 | 70000 | 75000 | 80000 | 85000 | 90000 | 95000 | 100000 | 105000 | 106860 |  |
| **Table S3.** Diversity analysis results for the CHEMBL database Cont. | PHPFP_3 | 813 | 971 | 1073 | 1110 | 1186 | 1165 | 1175 | 1196 | 1200 | 1202 | 1192 | 1196 | 1200 | 1202 | 1206 | 1206 | 1207 | 1207 | 1207 | 1207 | 1207 | 1207 | This table presents the number of targets covered by each one of the different subset sizes using 25 fingerprints and a random selection. |
|  | PHPFP_2 | 862 | 1048 | 1128 | 1157 | 1118 | 1188 | 1192 | 1202 | 1205 | 1205 | 1102 | 1196 | 1200 | 1202 | 1206 | 1206 | 1207 | 1207 | 1207 | 1207 | 1207 | 1207 |  |
|  | PHFP_4 | 808 | 974 | 1068 | 1106 | 1138 | 1151 | 1157 | 1173 | 1175 | 1179 | 1192 | 1196 | 1200 | 1202 | 1206 | 1206 | 1207 | 1207 | 1207 | 1207 | 1207 | 1207 |  |
|  | PHFP_3 | 830 | 1008 | 1096 | 1139 | 1131 | 1184 | 1191 | 1195 | 1197 | 1198 | 1192 | 1196 | 1200 | 1202 | 1206 | 1206 | 1207 | 1207 | 1207 | 1207 | 1207 | 1207 |  |
|  | PHFP_2 | 846 | 1023 | 1107 | 1139 | 1166 | 1193 | 1199 | 1198 | 1201 | 1201 | 1192 | 1196 | 1200 | 1202 | 1206 | 1206 | 1207 | 1207 | 1207 | 1207 | 1207 | 1207 |  |
|  | MDL | 893 | 1067 | 1143 | 1170 | 1185 | 1196 | 1198 | 1204 | 1205 | 1205 | 1192 | 1196 | 1200 | 1202 | 1206 | 1206 | 1207 | 1207 | 1207 | 1207 | 1207 | 1207 |  |
|  | ECFP_6 | 949 | 1102 | 1153 | 1183 | 1196 | 1202 | 1203 | 1205 | 1207 | 1207 | 1192 | 1196 | 1200 | 1202 | 1206 | 1206 | 1207 | 1207 | 1207 | 1207 | 1207 | 1207 |  |
|  | ECFP_4 | 970 | 1117 | 1156 | 1182 | 1194 | 1201 | 1203 | 12-2 | 1204 | 1204 | 1192 | 1196 | 1200 | 1202 | 1206 | 1206 | 1207 | 1207 | 1207 | 1207 | 1207 | 1207 |  |
|  | ECFP_2 | 938 | 1103 | 1156 | 1184 | 1193 | 1198 | 1202 | 1203 | 1205 | 1205 | 1192 | 1196 | 1200 | 1202 | 1206 | 1206 | 1207 | 1207 | 1207 | 1207 | 1207 | 1207 |  |
|  | FCFP_6 | 947 | 1083 | 1161 | 1182 | 1193 | 1202 | 1203 | 1202 | 1205 | 1205 | 1192 | 1196 | 1200 | 1202 | 1206 | 1206 | 1207 | 1207 | 1207 | 1207 | 1207 | 1207 |  |
|  | FCFP_4 | 935 | 1089 | 1151 | 1177 | 1191 | 1198 | 1200 | 1203 | 1205 | 1205 | 1192 | 1196 | 1200 | 1202 | 1206 | 1206 | 1207 | 1207 | 1207 | 1207 | 1207 | 1207 |  |
|  | FCFP_2 | 890 | 1041 | 1116 | 1156 | 1168 | 1187 | 1194 | 1196 | 1199 | 1200 | 1192 | 1196 | 1200 | 1202 | 1206 | 1206 | 1207 | 1207 | 1207 | 1207 | 1207 | 1207 |  |
|  | Random | 813 | 964 | 1036 | 1091 | 1122 | 1140 | 1153 | 1168 | 1177 | 1185 | 1192 | 1196 | 1200 | 1203 | 1205 | 1205 | 1206 | 1206 | 1206 | 1207 | 1207 | 1207 |  |
|  | Subset Size | 5000 | 10000 | 15000 | 20000 | 25000 | 30000 | 35000 | 40000 | 45000 | 50000 | 55000 | 60000 | 65000 | 70000 | 75000 | 80000 | 85000 | 90000 | 95000 | 100000 | 105000 | 106860 |  |

| **Table S4**. Similarity analysis results for the active drug Carbinoxamine (Histamine H1 receptor) | PHRFC_4 | 3 | 3 | 3 | 3 | 3 | 3 | 3 | 3 | 3 | 3 | 3 | 3 | 3 | 3 | 3 | 3 | 3 | 3 | 3 | 3 | 3 | 3 | 3 | 3 | 3 | 3 | 3 | 3 | 3 | 3 | 3 | 29 | This table presents the number active compounds identified within a certain % of the Drug Bank library using 25 fingerprints. |  |
| --- | --- | --- | --- | --- | --- | --- | --- | --- | --- | --- | --- | --- | --- | --- | --- | --- | --- | --- | --- | --- | --- | --- | --- | --- | --- | --- | --- | --- | --- | --- | --- | --- | --- | --- | --- |
|  | PHRFC_3 | 4 | 6 | 7 | 9 | 10 | 13 | 13 | 13 | 13 | 13 | 13 | 13 | 13 | 13 | 13 | 13 | 13 | 13 | 13 | 13 | 13 | 13 | 13 | 13 | 13 | 13 | 13 | 13 | 13 | 13 | 13 | 29 |  |  |
|  | PHRFC_2 | 4 | 5 | 6 | 6 | 6 | 6 | 8 | 10 | 11 | 11 | 13 | 15 | 15 | 15 | 16 | 16 | 17 | 19 | 19 | 20 | 20 | 20 | 20 | 20 | 20 | 20 | 20 | 20 | 20 | 20 | 20 | 29 |  |  |
|  | PHPFC_4 | 3 | 3 | 3 | 3 | 3 | 3 | 3 | 3 | 3 | 3 | 3 | 3 | 3 | 3 | 3 | 3 | 3 | 3 | 3 | 3 | 3 | 3 | 3 | 3 | 3 | 3 | 3 | 3 | 3 | 3 | 3 | 29 |  |  |
|  | PHPFC_3 | 4 | 6 | 8 | 10 | 12 | 14 | 14 | 14 | 14 | 14 | 14 | 14 | 14 | 14 | 14 | 14 | 14 | 14 | 14 | 14 | 14 | 14 | 14 | 14 | 14 | 14 | 14 | 14 | 14 | 14 | 14 | 29 |  |  |
|  | PHPFC_2 | 4 | 5 | 6 | 6 | 6 | 6 | 8 | 10 | 13 | 13 | 14 | 14 | 14 | 14 | 14 | 15 | 16 | 16 | 17 | 18 | 18 | 18 | 18 | 18 | 18 | 18 | 18 | 18 | 18 | 18 | 18 | 29 |  |  |
|  | PHFC_4 | 5 | 5 | 5 | 5 | 5 | 5 | 5 | 5 | 5 | 5 | 5 | 5 | 5 | 5 | 5 | 5 | 5 | 5 | 5 | 5 | 5 | 5 | 5 | 5 | 5 | 5 | 5 | 5 | 5 | 5 | 5 | 29 |  |  |
|  | PHFC_3 | 4 | 7 | 9 | 9 | 12 | 12 | 13 | 15 | 17 | 18 | 18 | 20 | 20 | 21 | 21 | 21 | 21 | 21 | 21 | 21 | 21 | 21 | 21 | 21 | 21 | 21 | 21 | 21 | 21 | 21 | 21 | 29 |  |  |
|  | PHFC_2 | 4 | 4 | 6 | 6 | 7 | 10 | 11 | 13 | 14 | 16 | 17 | 17 | 17 | 17 | 17 | 18 | 22 | 23 | 25 | 25 | 26 | 27 | 28 | 28 | 28 | 28 | 29 | 29 | 29 | 29 | 29 | 29 |  |  |
|  | PHRFP_4 | 3 | 3 | 3 | 3 | 3 | 3 | 3 | 3 | 3 | 3 | 3 | 3 | 3 | 3 | 3 | 3 | 3 | 3 | 3 | 3 | 3 | 3 | 3 | 3 | 3 | 3 | 3 | 3 | 3 | 3 | 3 | 29 |  |  |
|  | PHRFP_3 | 4 | 6 | 10 | 10 | 12 | 13 | 13 | 13 | 13 | 13 | 13 | 13 | 13 | 13 | 13 | 13 | 13 | 13 | 13 | 13 | 13 | 13 | 13 | 13 | 13 | 13 | 13 | 13 | 13 | 13 | 13 | 29 |  |  |
|  | PHRFP_2 | 4 | 6 | 6 | 6 | 6 | 7 | 7 | 8 | 10 | 10 | 13 | 14 | 15 | 16 | 16 | 16 | 18 | 18 | 19 | 20 | 20 | 20 | 20 | 20 | 20 | 20 | 20 | 20 | 20 | 20 | 20 | 29 |  |  |
|  | PHPFP_4 | 3 | 3 | 3 | 3 | 3 | 3 | 3 | 3 | 3 | 3 | 3 | 3 | 3 | 3 | 3 | 3 | 3 | 3 | 3 | 3 | 3 | 3 | 3 | 3 | 3 | 3 | 3 | 3 | 3 | 3 | 3 | 29 |  |  |
|  | % Library | 1 | 2 | 3 | 4 | 5 | 6 | 7 | 8 | 9 | 10 | 11 | 12 | 13 | 14 | 15 | 20 | 25 | 30 | 35 | 40 | 45 | 50 | 55 | 60 | 65 | 70 | 75 | 80 | 85 | 90 | 95 | 100 |  |  |
| **Table S4**. Similarity analysis results for the active drug Carbinoxamine (Histamine H1 receptor) Cont. | | PHPFP_3 | 4 | 7 | 10 | 11 | 13 | 14 | 14 | 14 | 14 | 14 | 14 | 14 | 14 | 14 | 14 | 14 | 14 | 14 | 14 | 14 | 14 | 14 | 14 | 14 | 14 | 14 | 14 | 14 | 14 | 14 | 14 | 29 | This table presents the number active compounds identified within a certain % of the Drug Bank library using 25 fingerprints. |
|  |  | PHPFP_2 | 4 | 5 | 6 | 6 | 6 | 6 | 6 | 6 | 9 | 10 | 13 | 13 | 14 | 15 | 15 | 15 | 17 | 17 | 18 | 18 | 18 | 18 | 18 | 18 | 18 | 18 | 18 | 18 | 18 | 18 | 18 | 29 |  |
|  |  | PHFP_4 | 5 | 5 | 5 | 5 | 5 | 5 | 5 | 5 | 5 | 5 | 5 | 5 | 5 | 5 | 5 | 5 | 5 | 5 | 5 | 5 | 5 | 5 | 5 | 5 | 5 | 5 | 5 | 5 | 5 | 5 | 5 | 29 |  |
|  |  | PHFP_3 | 4 | 6 | 7 | 10 | 13 | 16 | 17 | 20 | 20 | 20 | 21 | 21 | 21 | 21 | 21 | 21 | 21 | 21 | 21 | 21 | 21 | 21 | 21 | 21 | 21 | 21 | 21 | 21 | 21 | 21 | 21 | 29 |  |
|  |  | PHFP_2 | 4 | 4 | 4 | 7 | 10 | 13 | 14 | 15 | 15 | 17 | 17 | 17 | 18 | 19 | 19 | 19 | 20 | 21 | 22 | 23 | 26 | 26 | 27 | 28 | 29 | 29 | 29 | 29 | 29 | 29 | 29 | 29 |  |
|  |  | MDL | 5 | 9 | 12 | 13 | 15 | 16 | 19 | 19 | 19 | 21 | 23 | 24 | 24 | 24 | 24 | 25 | 27 | 27 | 27 | 29 | 29 | 29 | 29 | 29 | 29 | 29 | 29 | 29 | 29 | 29 | 29 | 29 |  |
|  |  | ECFP_6 | 5 | 9 | 12 | 14 | 16 | 17 | 18 | 18 | 19 | 20 | 21 | 21 | 21 | 22 | 23 | 24 | 25 | 26 | 27 | 28 | 28 | 29 | 29 | 29 | 29 | 29 | 29 | 29 | 29 | 29 | 29 | 29 |  |
|  |  | ECFP_4 | 5 | 9 | 12 | 15 | 16 | 18 | 18 | 19 | 19 | 20 | 21 | 21 | 22 | 23 | 23 | 25 | 25 | 27 | 28 | 28 | 28 | 29 | 29 | 29 | 29 | 29 | 29 | 29 | 29 | 29 | 29 | 29 |  |
|  |  | ECFP_2 | 5 | 9 | 11 | 14 | 16 | 17 | 17 | 18 | 19 | 19 | 20 | 22 | 23 | 23 | 23 | 24 | 24 | 25 | 27 | 28 | 28 | 28 | 29 | 29 | 29 | 29 | 29 | 29 | 29 | 29 | 29 | 29 |  |
|  |  | FCFP_6 | 5 | 9 | 11 | 12 | 14 | 14 | 14 | 15 | 16 | 17 | 18 | 19 | 19 | 19 | 19 | 21 | 24 | 25 | 26 | 27 | 29 | 29 | 29 | 29 | 29 | 29 | 29 | 29 | 29 | 29 | 29 | 29 |  |
|  |  | FCFP_4 | 5 | 9 | 11 | 13 | 14 | 15 | 15 | 16 | 16 | 17 | 18 | 18 | 18 | 18 | 18 | 23 | 24 | 25 | 27 | 27 | 29 | 29 | 29 | 29 | 29 | 29 | 29 | 29 | 29 | 29 | 29 | 29 |  |
|  |  | FCFP_2 | 5 | 8 | 11 | 13 | 15 | 16 | 16 | 16 | 16 | 16 | 16 | 17 | 17 | 18 | 18 | 22 | 23 | 26 | 26 | 28 | 28 | 29 | 29 | 29 | 29 | 29 | 29 | 29 | 29 | 29 | 29 | 29 |  |
|  |  | % Library | 1 | 2 | 3 | 4 | 5 | 6 | 7 | 8 | 9 | 10 | 11 | 12 | 13 | 14 | 15 | 20 | 25 | 30 | 35 | 40 | 45 | 50 | 55 | 60 | 65 | 70 | 75 | 80 | 85 | 90 | 95 | 100 |  |

| **Table S5**. Similarity analysis results for the active drug Fluocinolone acetonide (Glucocorticiod receptor) | PHRFC_4 | 5 | 10 | 14 | 19 | 23 | 23 | 23 | 23 | 23 | 23 | 23 | 23 | 23 | 23 | 23 | 23 | 23 | 23 | 23 | 23 | 23 | 23 | 23 | 23 | 23 | 23 | 23 | 23 | 23 | 23 | 23 | 29 | This table presents the number active compounds identified within a certain % of the Drug Bank library using 25 fingerprints. |
| --- | --- | --- | --- | --- | --- | --- | --- | --- | --- | --- | --- | --- | --- | --- | --- | --- | --- | --- | --- | --- | --- | --- | --- | --- | --- | --- | --- | --- | --- | --- | --- | --- | --- | --- |
|  | PHRFC_3 | 4 | 9 | 14 | 19 | 24 | 28 | 29 | 29 | 29 | 29 | 29 | 29 | 29 | 29 | 29 | 29 | 29 | 29 | 29 | 29 | 29 | 29 | 29 | 29 | 29 | 29 | 29 | 29 | 92 | 29 | 29 | 29 |  |
|  | PHRFC_2 | 5 | 9 | 14 | 19 | 21 | 23 | 26 | 26 | 28 | 29 | 29 | 29 | 29 | 29 | 29 | 29 | 29 | 29 | 29 | 29 | 29 | 29 | 29 | 29 | 29 | 29 | 29 | 29 | 92 | 29 | 29 | 29 |  |
|  | PHPFC_4 | 5 | 9 | 14 | 19 | 20 | 20 | 20 | 20 | 20 | 20 | 20 | 20 | 20 | 20 | 20 | 20 | 20 | 20 | 20 | 20 | 20 | 20 | 20 | 20 | 20 | 20 | 20 | 20 | 20 | 20 | 20 | 29 |  |
|  | PHPFC_3 | 5 | 9 | 14 | 19 | 24 | 28 | 29 | 29 | 29 | 29 | 29 | 29 | 29 | 29 | 29 | 29 | 29 | 29 | 29 | 29 | 29 | 29 | 29 | 29 | 29 | 29 | 29 | 29 | 92 | 29 | 29 | 29 |  |
|  | PHPFC_2 | 5 | 10 | 14 | 19 | 24 | 29 | 29 | 29 | 29 | 29 | 29 | 29 | 29 | 29 | 29 | 29 | 29 | 29 | 29 | 29 | 29 | 29 | 29 | 29 | 29 | 29 | 29 | 29 | 92 | 29 | 29 | 29 |  |
|  | PHFC_4 | 5 | 9 | 14 | 19 | 24 | 29 | 29 | 29 | 29 | 29 | 29 | 29 | 29 | 29 | 29 | 29 | 29 | 29 | 29 | 29 | 29 | 29 | 29 | 29 | 29 | 29 | 29 | 29 | 92 | 29 | 29 | 29 |  |
|  | PHFC_3 | 5 | 9 | 14 | 19 | 24 | 29 | 29 | 29 | 29 | 29 | 29 | 29 | 29 | 29 | 29 | 29 | 29 | 29 | 29 | 29 | 29 | 29 | 29 | 29 | 29 | 29 | 29 | 29 | 92 | 29 | 29 | 29 |  |
|  | PHFC_2 | 5 | 9 | 14 | 19 | 24 | 28 | 29 | 29 | 29 | 29 | 29 | 29 | 29 | 29 | 29 | 29 | 29 | 29 | 29 | 29 | 29 | 29 | 29 | 29 | 29 | 29 | 29 | 29 | 92 | 29 | 29 | 29 |  |
|  | PHRFP_4 | 5 | 10 | 14 | 19 | 23 | 23 | 23 | 23 | 23 | 23 | 23 | 23 | 23 | 23 | 23 | 23 | 23 | 23 | 23 | 23 | 23 | 23 | 23 | 23 | 23 | 23 | 23 | 23 | 23 | 23 | 23 | 29 |  |
|  | PHRFP_3 | 5 | 9 | 14 | 19 | 24 | 28 | 29 | 29 | 29 | 29 | 29 | 29 | 29 | 29 | 29 | 29 | 29 | 29 | 29 | 29 | 29 | 29 | 29 | 29 | 29 | 29 | 29 | 29 | 92 | 29 | 29 | 29 |  |
|  | PHRFP_2 | 5 | 9 | 14 | 19 | 22 | 24 | 26 | 28 | 29 | 29 | 29 | 29 | 29 | 29 | 29 | 29 | 29 | 29 | 29 | 29 | 29 | 29 | 29 | 29 | 29 | 29 | 29 | 29 | 29 | 29 | 92 | 29 |  |
|  | PHPFP_4 | 5 | 9 | 14 | 19 | 20 | 20 | 20 | 20 | 20 | 20 | 20 | 20 | 20 | 20 | 20 | 20 | 20 | 20 | 20 | 20 | 20 | 20 | 20 | 20 | 20 | 20 | 20 | 20 | 20 | 20 | 20 | 29 |  |
|  | PHPFP_3 | 5 | 9 | 14 | 19 | 24 | 28 | 29 | 29 | 29 | 29 | 29 | 29 | 29 | 29 | 29 | 29 | 29 | 29 | 29 | 29 | 29 | 29 | 29 | 29 | 29 | 29 | 29 | 29 | 29 | 29 | 92 | 29 |  |
|  | % Library | 1 | 2 | 3 | 4 | 5 | 6 | 7 | 8 | 9 | 10 | 11 | 12 | 13 | 14 | 15 | 20 | 25 | 30 | 35 | 40 | 45 | 50 | 55 | 60 | 65 | 70 | 75 | 80 | 85 | 90 | 95 | 100 |  |
| **Table S5**. Similarity analysis results for the active drug Fluocinolone acetonide (Glucocorticiod receptor) Cont. | PHPFP_2 | 5 | 10 | 14 | 19 | 24 | 29 | 29 | 29 | 29 | 29 | 29 | 29 | 29 | 29 | 29 | 29 | 29 | 29 | 29 | 29 | 29 | 29 | 29 | 29 | 29 | 29 | 29 | 29 | 29 | 29 | 92 | 29 | This table presents the number active compounds identified within a certain % of the Drug Bank library using 25 fingerprints. |
|  | PHFP_4 | 5 | 9 | 14 | 19 | 24 | 29 | 29 | 29 | 29 | 29 | 29 | 29 | 29 | 29 | 29 | 29 | 29 | 29 | 29 | 29 | 29 | 29 | 29 | 29 | 29 | 29 | 29 | 29 | 29 | 29 | 92 | 29 |  |
|  | PHFP_3 | 5 | 9 | 14 | 19 | 24 | 29 | 29 | 29 | 29 | 29 | 29 | 29 | 29 | 29 | 29 | 29 | 29 | 29 | 29 | 29 | 29 | 29 | 29 | 29 | 29 | 29 | 29 | 29 | 29 | 29 | 92 | 29 |  |
|  | PHFP_2 | 4 | 9 | 14 | 19 | 24 | 28 | 29 | 29 | 29 | 29 | 29 | 29 | 29 | 29 | 29 | 29 | 29 | 29 | 29 | 29 | 29 | 29 | 29 | 29 | 29 | 29 | 29 | 29 | 29 | 29 | 92 | 29 |  |
|  | MDL | 5 | 10 | 14 | 19 | 24 | 26 | 28 | 28 | 29 | 29 | 29 | 29 | 29 | 29 | 29 | 29 | 29 | 29 | 29 | 29 | 29 | 29 | 29 | 29 | 29 | 29 | 29 | 29 | 29 | 29 | 92 | 29 |  |
|  | ECFP_6 | 5 | 10 | 25 | 20 | 24 | 28 | 29 | 29 | 29 | 29 | 29 | 29 | 29 | 29 | 29 | 29 | 29 | 29 | 29 | 29 | 29 | 29 | 29 | 29 | 29 | 29 | 29 | 29 | 29 | 29 | 92 | 29 |  |
|  | ECFP_4 | 5 | 10 | 15 | 20 | 24 | 28 | 29 | 29 | 29 | 29 | 29 | 29 | 29 | 29 | 29 | 29 | 29 | 29 | 29 | 29 | 29 | 29 | 29 | 29 | 29 | 29 | 29 | 29 | 29 | 29 | 92 | 29 |  |
|  | ECFP_2 | 5 | 10 | 15 | 19 | 24 | 28 | 29 | 29 | 29 | 29 | 29 | 29 | 29 | 29 | 29 | 29 | 29 | 29 | 29 | 29 | 29 | 29 | 29 | 29 | 29 | 29 | 29 | 29 | 29 | 29 | 92 | 29 |  |
|  | FCFP_6 | 5 | 10 | 15 | 20 | 24 | 27 | 29 | 29 | 29 | 29 | 29 | 29 | 29 | 29 | 29 | 29 | 29 | 29 | 29 | 29 | 29 | 29 | 29 | 29 | 29 | 29 | 29 | 29 | 29 | 29 | 92 | 29 |  |
|  | FCFP_4 | 5 | 10 | 15 | 20 | 24 | 27 | 28 | 29 | 29 | 29 | 29 | 29 | 29 | 29 | 29 | 29 | 29 | 29 | 29 | 29 | 29 | 29 | 29 | 29 | 29 | 29 | 29 | 29 | 29 | 29 | 92 | 29 |  |
|  | FCFP_2 | 5 | 10 | 15 | 19 | 23 | 26 | 27 | 27 | 28 | 29 | 29 | 29 | 29 | 29 | 29 | 29 | 29 | 29 | 29 | 29 | 29 | 29 | 29 | 29 | 29 | 29 | 29 | 29 | 29 | 29 | 92 | 29 |  |
|  | % Library | 1 | 2 | 3 | 4 | 5 | 6 | 7 | 8 | 9 | 10 | 11 | 12 | 13 | 14 | 15 | 20 | 25 | 30 | 35 | 40 | 45 | 50 | 55 | 60 | 65 | 70 | 75 | 80 | 85 | 90 | 95 | 100 |  |

| **Table S6**. Similarity analysis results for the active drug Lymecycline (Antibiotic) | PHRFC_4 | 12 | 14 | 19 | 25 | 31 | 38 | 40 | 40 | 40 | 40 | 40 | 40 | 40 | 40 | 40 | 40 | 40 | 40 | 40 | 40 | 40 | 40 | 40 | 40 | 40 | 40 | 40 | 40 | 40 | 40 | 40 | 129 | This table presents the number active compounds identified within a certain % of the CMC library using 25 fingerprints. |
| --- | --- | --- | --- | --- | --- | --- | --- | --- | --- | --- | --- | --- | --- | --- | --- | --- | --- | --- | --- | --- | --- | --- | --- | --- | --- | --- | --- | --- | --- | --- | --- | --- | --- | --- |
|  | PHRFC_3 | 12 | 19 | 22 | 25 | 30 | 40 | 46 | 48 | 54 | 57 | 57 | 58 | 60 | 61 | 62 | 72 | 80 | 81 | 94 | 97 | 97 | 97 | 97 | 97 | 97 | 97 | 97 | 97 | 97 | 97 | 97 | 129 |  |
|  | PHRFC_2 | 13 | 19 | 29 | 32 | 34 | 43 | 47 | 53 | 57 | 61 | 65 | 66 | 68 | 70 | 71 | 79 | 90 | 101 | 109 | 117 | 118 | 122 | 123 | 125 | 125 | 127 | 127 | 127 | 127 | 128 | 129 | 129 |  |
|  | PHPFC_4 | 9 | 14 | 14 | 14 | 14 | 14 | 14 | 14 | 14 | 14 | 14 | 14 | 14 | 14 | 14 | 14 | 14 | 14 | 14 | 14 | 14 | 14 | 14 | 14 | 14 | 14 | 14 | 14 | 14 | 14 | 14 | 129 |  |
|  | PHPFC_3 | 12 | 16 | 19 | 21 | 24 | 29 | 31 | 35 | 38 | 38 | 42 | 43 | 44 | 48 | 49 | 65 | 65 | 65 | 65 | 65 | 65 | 65 | 65 | 65 | 65 | 65 | 65 | 65 | 65 | 65 | 65 | 129 |  |
|  | PHPFC_2 | 14 | 16 | 23 | 29 | 34 | 40 | 44 | 47 | 50 | 52 | 55 | 57 | 58 | 62 | 65 | 76 | 86 | 96 | 109 | 114 | 114 | 115 | 115 | 116 | 117 | 119 | 122 | 122 | 125 | 125 | 125 | 129 |  |
|  | PHFC_4 | 11 | 12 | 24 | 31 | 41 | 46 | 53 | 59 | 62 | 65 | 70 | 72 | 74 | 76 | 81 | 89 | 99 | 104 | 104 | 104 | 104 | 104 | 104 | 104 | 104 | 104 | 104 | 104 | 104 | 104 | 104 | 129 |  |
|  | PHFC_3 | 10 | 16 | 24 | 36 | 40 | 47 | 56 | 62 | 63 | 70 | 72 | 74 | 77 | 79 | 79 | 92 | 99 | 106 | 113 | 117 | 119 | 122 | 124 | 125 | 126 | 127 | 127 | 128 | 128 | 128 | 128 | 129 |  |
|  | PHFC_2 | 13 | 20 | 27 | 30 | 37 | 44 | 50 | 58 | 66 | 70 | 71 | 74 | 78 | 81 | 82 | 92 | 102 | 106 | 112 | 116 | 117 | 121 | 123 | 123 | 128 | 128 | 128 | 128 | 128 | 128 | 128 | 129 |  |
|  | PHRFP_4 | 11 | 14 | 24 | 26 | 32 | 40 | 41 | 41 | 41 | 41 | 41 | 41 | 41 | 41 | 41 | 41 | 41 | 41 | 41 | 41 | 41 | 41 | 41 | 41 | 41 | 41 | 41 | 41 | 41 | 41 | 41 | 129 |  |
|  | PHRFP_3 | 12 | 18 | 27 | 30 | 40 | 43 | 48 | 48 | 53 | 53 | 55 | 57 | 61 | 62 | 64 | 71 | 80 | 88 | 92 | 97 | 97 | 97 | 97 | 97 | 97 | 97 | 97 | 97 | 97 | 97 | 97 | 129 |  |
|  | PHRFP_2 | 15 | 21 | 36 | 40 | 43 | 46 | 53 | 55 | 58 | 59 | 64 | 64 | 66 | 68 | 71 | 77 | 85 | 92 | 105 | 109 | 113 | 116 | 124 | 124 | 125 | 125 | 127 | 127 | 128 | 128 | 129 | 129 |  |
|  | PHPFP_4 | 9 | 15 | 15 | 15 | 15 | 15 | 15 | 15 | 15 | 15 | 15 | 15 | 15 | 15 | 15 | 15 | 15 | 15 | 15 | 15 | 15 | 15 | 15 | 15 | 15 | 15 | 15 | 15 | 15 | 15 | 15 | 129 |  |
|  | PHPFP_3 | 9 | 12 | 13 | 19 | 23 | 26 | 32 | 33 | 37 | 40 | 43 | 44 | 48 | 48 | 49 | 65 | 65 | 65 | 65 | 65 | 65 | 65 | 65 | 65 | 65 | 65 | 65 | 65 | 65 | 65 | 65 | 129 |  |
|  | % Library | 1 | 2 | 3 | 4 | 5 | 6 | 7 | 8 | 9 | 10 | 11 | 12 | 13 | 14 | 15 | 20 | 25 | 30 | 35 | 40 | 45 | 50 | 55 | 60 | 65 | 70 | 75 | 80 | 85 | 90 | 95 | 100 |  |
| **Table S6**. Similarity analysis results for the active drug Lymecycline (Antibiotic) Cont. | PHPFP_2 | 10 | 16 | 19 | 23 | 28 | 38 | 43 | 50 | 52 | 56 | 63 | 65 | 67 | 68 | 69 | 82 | 96 | 103 | 106 | 108 | 110 | 113 | 115 | 117 | 120 | 121 | 121 | 123 | 125 | 125 | 125 | 129 | This table presents the number active compounds identified within a certain % of the CMC library using 25 fingerprints. |
|  | PHFP_4 | 9 | 14 | 21 | 31 | 38 | 43 | 49 | 54 | 62 | 69 | 73 | 73 | 76 | 78 | 79 | 90 | 100 | 104 | 104 | 104 | 104 | 104 | 104 | 104 | 104 | 104 | 104 | 104 | 104 | 104 | 104 | 129 |  |
|  | PHFP_3 | 15 | 21 | 26 | 31 | 39 | 49 | 54 | 60 | 62 | 68 | 75 | 77 | 78 | 78 | 79 | 90 | 97 | 102 | 109 | 112 | 116 | 120 | 123 | 123 | 126 | 126 | 127 | 128 | 128 | 128 | 128 | 129 |  |
|  | PHFP_2 | 12 | 24 | 28 | 37 | 41 | 49 | 58 | 60 | 64 | 67 | 68 | 73 | 76 | 79 | 80 | 91 | 98 | 102 | 105 | 108 | 109 | 112 | 115 | 115 | 116 | 117 | 120 | 122 | 125 | 127 | 128 | 129 |  |
|  | MDL | 9 | 10 | 11 | 13 | 17 | 19 | 20 | 22 | 23 | 24 | 26 | 26 | 30 | 31 | 34 | 46 | 55 | 61 | 70 | 74 | 79 | 87 | 95 | 99 | 99 | 99 | 104 | 111 | 120 | 122 | 126 | 129 |  |
|  | ECFP_6 | 10 | 14 | 20 | 25 | 28 | 30 | 32 | 35 | 38 | 38 | 38 | 42 | 44 | 47 | 49 | 52 | 60 | 68 | 77 | 82 | 87 | 91 | 98 | 101 | 105 | 110 | 111 | 115 | 119 | 119 | 125 | 129 |  |
|  | ECFP_4 | 10 | 14 | 18 | 25 | 26 | 30 | 32 | 35 | 39 | 40 | 40 | 43 | 45 | 46 | 48 | 53 | 59 | 66 | 76 | 83 | 89 | 91 | 95 | 100 | 104 | 109 | 111 | 113 | 118 | 119 | 126 | 129 |  |
|  | ECFP_2 | 11 | 16 | 17 | 21 | 27 | 31 | 33 | 37 | 42 | 43 | 45 | 48 | 48 | 49 | 50 | 54 | 63 | 73 | 78 | 83 | 90 | 91 | 93 | 99 | 104 | 108 | 111 | 114 | 118 | 119 | 128 | 129 |  |
|  | FCFP_6 | 9 | 9 | 10 | 12 | 17 | 18 | 20 | 21 | 24 | 24 | 25 | 26 | 30 | 32 | 36 | 48 | 54 | 59 | 66 | 75 | 82 | 85 | 92 | 98 | 99 | 101 | 105 | 108 | 118 | 124 | 127 | 129 |  |
|  | FCFP_4 | 9 | 10 | 11 | 139 | 17 | 19 | 20 | 22 | 23 | 24 | 26 | 26 | 30 | 31 | 34 | 46 | 55 | 61 | 70 | 74 | 79 | 87 | 95 | 99 | 99 | 99 | 104 | 11 | 120 | 122 | 126 | 129 |  |
|  | FCFP_2 | 9 | 13 | 18 | 19 | 23 | 23 | 25 | 28 | 33 | 34 | 37 | 44 | 46 | 48 | 49 | 59 | 68 | 78 | 85 | 90 | 93 | 94 | 98 | 102 | 107 | 113 | 117 | 121 | 123 | 125 | 127 | 129 |  |
|  | % Library | 1 | 2 | 3 | 4 | 5 | 6 | 7 | 8 | 9 | 10 | 11 | 12 | 13 | 14 | 15 | 20 | 25 | 30 | 35 | 40 | 45 | 50 | 55 | 60 | 65 | 70 | 75 | 80 | 85 | 90 | 95 | 100 |  |

| **Table S7.** Similarity analysis results for the active drug Haloperidol (Antipsychotic) | PHRFC_4 | 20 | 20 | 20 | 20 | 20 | 20 | 20 | 20 | 20 | 20 | 20 | 20 | 20 | 20 | 20 | 20 | 20 | 20 | 20 | 20 | 20 | 20 | 20 | 20 | 20 | 20 | 20 | 20 | 20 | 20 | 20 | 164 | This table presents the number active compounds identified within a certain % of the CMC library using 25 fingerprints. |
| --- | --- | --- | --- | --- | --- | --- | --- | --- | --- | --- | --- | --- | --- | --- | --- | --- | --- | --- | --- | --- | --- | --- | --- | --- | --- | --- | --- | --- | --- | --- | --- | --- | --- | --- |
|  | PHRFC_3 | 23 | 27 | 34 | 35 | 35 | 35 | 35 | 35 | 35 | 35 | 35 | 35 | 35 | 35 | 35 | 35 | 35 | 35 | 35 | 35 | 35 | 35 | 35 | 35 | 35 | 35 | 35 | 35 | 35 | 35 | 35 | 164 |  |
|  | PHRFC_2 | 23 | 28 | 28 | 29 | 29 | 29 | 33 | 36 | 38 | 41 | 43 | 44 | 46 | 47 | 48 | 59 | 65 | 73 | 84 | 93 | 105 | 118 | 121 | 125 | 129 | 129 | 129 | 129 | 129 | 129 | 129 | 164 |  |
|  | PHPFC_4 | 21 | 21 | 21 | 21 | 21 | 21 | 21 | 21 | 21 | 21 | 21 | 21 | 21 | 21 | 21 | 21 | 21 | 21 | 21 | 21 | 21 | 21 | 21 | 21 | 21 | 21 | 21 | 21 | 21 | 21 | 21 | 164 |  |
|  | PHPFC_3 | 21 | 26 | 35 | 35 | 35 | 35 | 35 | 35 | 35 | 35 | 35 | 35 | 35 | 35 | 35 | 35 | 35 | 35 | 35 | 35 | 35 | 35 | 35 | 35 | 35 | 35 | 35 | 35 | 35 | 35 | 35 | 164 |  |
|  | PHPFC_2 | 14 | 20 | 29 | 30 | 33 | 34 | 35 | 38 | 38 | 41 | 44 | 47 | 50 | 52 | 55 | 61 | 65 | 71 | 75 | 91 | 100 | 100 | 100 | 100 | 100 | 100 | 100 | 100 | 100 | 100 | 100 | 164 |  |
|  | PHFC_4 | 28 | 30 | 30 | 30 | 30 | 30 | 30 | 30 | 30 | 30 | 30 | 30 | 30 | 30 | 30 | 30 | 30 | 30 | 30 | 30 | 30 | 30 | 30 | 30 | 30 | 30 | 30 | 30 | 30 | 30 | 30 | 164 |  |
|  | PHFC_3 | 19 | 33 | 38 | 45 | 54 | 57 | 63 | 67 | 74 | 80 | 85 | 89 | 94 | 94 | 96 | 99 | 99 | 99 | 99 | 99 | 99 | 99 | 99 | 99 | 99 | 99 | 99 | 99 | 99 | 99 | 99 | 164 |  |
|  | PHFC_2 | 17 | 26 | 31 | 34 | 41 | 48 | 52 | 54 | 56 | 63 | 68 | 71 | 73 | 75 | 77 | 94 | 102 | 117 | 124 | 125 | 131 | 135 | 139 | 141 | 150 | 151 | 154 | 156 | 162 | 162 | 162 | 164 |  |
|  | PHRFP_4 | 20 | 20 | 20 | 20 | 20 | 20 | 20 | 20 | 20 | 20 | 20 | 20 | 20 | 20 | 20 | 20 | 20 | 20 | 20 | 20 | 20 | 20 | 20 | 20 | 20 | 20 | 20 | 20 | 20 | 20 | 20 | 164 |  |
|  | PHRFP_3 | 22 | 27 | 34 | 35 | 35 | 35 | 35 | 35 | 35 | 35 | 35 | 35 | 35 | 35 | 35 | 35 | 35 | 35 | 35 | 35 | 35 | 35 | 35 | 35 | 35 | 35 | 35 | 35 | 35 | 35 | 35 | 164 |  |
|  | PHRFP_2 | 20 | 27 | 30 | 34 | 34 | 38 | 40 | 42 | 43 | 44 | 48 | 49 | 52 | 53 | 55 | 69 | 77 | 84 | 89 | 90 | 94 | 96 | 111 | 116 | 130 | 130 | 130 | 130 | 130 | 130 | 130 | 164 |  |
|  | PHPFP_4 | 21 | 21 | 21 | 21 | 21 | 21 | 21 | 21 | 21 | 21 | 21 | 21 | 21 | 21 | 21 | 21 | 21 | 21 | 21 | 21 | 21 | 21 | 21 | 21 | 21 | 21 | 21 | 21 | 21 | 21 | 21 | 164 |  |
|  | PHPFP_3 | 20 | 27 | 35 | 35 | 35 | 35 | 35 | 35 | 35 | 35 | 35 | 35 | 35 | 35 | 35 | 35 | 35 | 35 | 35 | 35 | 35 | 35 | 35 | 35 | 35 | 35 | 35 | 35 | 35 | 35 | 35 | 164 |  |
|  | % Library | 1 | 2 | 3 | 4 | 5 | 6 | 7 | 8 | 9 | 10 | 11 | 12 | 13 | 14 | 15 | 20 | 25 | 30 | 35 | 40 | 45 | 50 | 55 | 60 | 65 | 70 | 75 | 80 | 85 | 90 | 95 | 100 |  |
| **Table S7.** Similarity analysis results for the active drug Haloperidol (Antipsychotic) Cont. | PHPFP_2 | 16 | 24 | 29 | 30 | 33 | 34 | 37 | 40 | 41 | 44 | 48 | 49 | 51 | 52 | 54 | 62 | 70 | 71 | 76 | 91 | 100 | 100 | 100 | 100 | 100 | 100 | 100 | 100 | 100 | 100 | 100 | 164 | This table presents the number active compounds identified within a certain % of the CMC library using 25 fingerprints. |
|  | PHFP_4 | 29 | 30 | 30 | 30 | 30 | 30 | 30 | 30 | 30 | 30 | 30 | 30 | 30 | 30 | 30 | 30 | 30 | 30 | 30 | 30 | 30 | 30 | 30 | 30 | 30 | 30 | 30 | 30 | 30 | 30 | 30 | 164 |  |
|  | PHFP_3 | 21 | 32 | 39 | 45 | 59 | 65 | 71 | 76 | 80 | 86 | 87 | 91 | 93 | 96 | 98 | 98 | 99 | 99 | 99 | 99 | 99 | 99 | 99 | 99 | 99 | 99 | 99 | 99 | 99 | 99 | 99 | 164 |  |
|  | PHFP_2 | 18 | 25 | 34 | 35 | 37 | 44 | 51 | 56 | 59 | 60 | 62 | 64 | 68 | 73 | 77 | 92 | 104 | 111 | 121 | 125 | 130 | 137 | 139 | 141 | 143 | 150 | 151 | 156 | 160 | 163 | 163 | 164 |  |
|  | MDL | 17 | 24 | 29 | 38 | 47 | 59 | 65 | 72 | 77 | 82 | 84 | 89 | 94 | 97 | 100 | 112 | 123 | 129 | 133 | 140 | 145 | 147 | 154 | 154 | 155 | 157 | 158 | 160 | 161 | 163 | 163 | 164 |  |
|  | ECFP_6 | 30 | 38 | 43 | 48 | 53 | 55 | 58 | 66 | 69 | 75 | 78 | 81 | 84 | 85 | 89 | 96 | 113 | 117 | 123 | 128 | 131 | 134 | 137 | 138 | 140 | 148 | 153 | 154 | 156 | 162 | 164 | 164 |  |
|  | ECFP_4 | 26 | 39 | 48 | 52 | 55 | 61 | 67 | 73 | 78 | 81 | 84 | 85 | 86 | 88 | 90 | 106 | 116 | 118 | 124 | 130 | 134 | 135 | 137 | 140 | 143 | 149 | 154 | 155 | 159 | 162 | 163 | 164 |  |
|  | ECFP_2 | 24 | 40 | 50 | 57 | 61 | 67 | 71 | 74 | 78 | 82 | 85 | 86 | 87 | 94 | 95 | 103 | 116 | 123 | 128 | 131 | 136 | 137 | 139 | 142 | 144 | 149 | 154 | 155 | 159 | 162 | 163 | 164 |  |
|  | FCFP_6 | 25 | 33 | 38 | 43 | 50 | 55 | 59 | 61 | 66 | 69 | 77 | 79 | 83 | 86 | 89 | 104 | 109 | 116 | 121 | 125 | 130 | 136 | 145 | 147 | 150 | 153 | 155 | 158 | 159 | 161 | 164 | 164 |  |
|  | FCFP_4 | 21 | 31 | 41 | 52 | 54 | 58 | 60 | 66 | 72 | 77 | 80 | 82 | 88 | 97 | 102 | 107 | 114 | 120 | 123 | 129 | 131 | 138 | 146 | 148 | 152 | 153 | 157 | 157 | 158 | 164 | 164 | 164 |  |
|  | FCFP_2 | 17 | 28 | 34 | 44 | 53 | 56 | 62 | 63 | 71 | 76 | 77 | 82 | 87 | 89 | 95 | 112 | 116 | 122 | 127 | 134 | 137 | 141 | 144 | 146 | 149 | 153 | 157 | 157 | 162 | 164 | 164 | 164 |  |
|  | % Library | 1 | 2 | 3 | 4 | 5 | 6 | 7 | 8 | 9 | 10 | 11 | 12 | 13 | 14 | 15 | 20 | 25 | 30 | 35 | 40 | 45 | 50 | 55 | 60 | 65 | 70 | 75 | 80 | 85 | 90 | 95 | 100 |  |

| **Table S8.** Similarity analysis results for the active drug CHEMBL488890 (Melanin-concentrating hormone receptor 1) | PHRFC_4 | 38 | 47 | 47 | 47 | 47 | 47 | 47 | 47 | 47 | 47 | 47 | 47 | 47 | 47 | 47 | 47 | 47 | 47 | 47 | 47 | 47 | 47 | 47 | 47 | 47 | 47 | 47 | 47 | 47 | 47 | 47 | 1174 | This table presents the number active compounds identified within a certain % of the CHEMBL library using 25 fingerprints. |
| --- | --- | --- | --- | --- | --- | --- | --- | --- | --- | --- | --- | --- | --- | --- | --- | --- | --- | --- | --- | --- | --- | --- | --- | --- | --- | --- | --- | --- | --- | --- | --- | --- | --- | --- |
|  | PHRFC_3 | 31 | 46 | 57 | 67 | 83 | 87 | 92 | 100 | 109 | 123 | 147 | 192 | 208 | 223 | 236 | 250 | 251 | 251 | 251 | 251 | 251 | 251 | 251 | 251 | 251 | 251 | 251 | 251 | 251 | 251 | 251 | 1174 |  |
|  | PHRFC_2 | 22 | 39 | 48 | 56 | 65 | 92 | 102 | 109 | 118 | 123 | 132 | 145 | 155 | 162 | 169 | 198 | 256 | 335 | 413 | 461 | 591 | 695 | 781 | 825 | 879 | 958 | 999 | 1047 | 1091 | 1128 | 1159 | 1174 |  |
|  | PHPFC_4 | 45 | 46 | 46 | 46 | 46 | 46 | 46 | 46 | 46 | 46 | 46 | 46 | 46 | 46 | 46 | 46 | 46 | 46 | 46 | 46 | 46 | 46 | 46 | 46 | 46 | 46 | 46 | 46 | 46 | 46 | 46 | 1174 |  |
|  | PHPFC_3 | 48 | 73 | 81 | 92 | 115 | 140 | 190 | 221 | 235 | 241 | 268 | 283 | 283 | 283 | 284 | 284 | 284 | 284 | 284 | 284 | 284 | 284 | 284 | 284 | 284 | 284 | 284 | 284 | 284 | 284 | 284 | 1174 |  |
|  | PHPFC_2 | 46 | 57 | 71 | 88 | 106 | 113 | 138 | 163 | 171 | 186 | 192 | 207 | 219 | 224 | 262 | 335 | 413 | 489 | 544 | 636 | 698 | 734 | 799 | 867 | 923 | 952 | 1010 | 1060 | 1114 | 1143 | 1143 | 1174 |  |
|  | PHFC_4 | 71 | 90 | 103 | 130 | 153 | 161 | 163 | 166 | 167 | 168 | 168 | 169 | 169 | 169 | 169 | 169 | 169 | 169 | 169 | 169 | 169 | 169 | 169 | 169 | 169 | 169 | 169 | 169 | 169 | 169 | 169 | 1174 |  |
|  | PHFC_3 | 62 | 77 | 87 | 98 | 109 | 117 | 130 | 145 | 158 | 180 | 196 | 213 | 227 | 253 | 271 | 353 | 419 | 459 | 491 | 568 | 623 | 708 | 789 | 883 | 907 | 928 | 928 | 928 | 928 | 928 | 928 | 1174 |  |
|  | PHFC_2 | 40 | 61 | 72 | 82 | 113 | 122 | 140 | 151 | 171 | 180 | 194 | 224 | 238 | 256 | 273 | 349 | 436 | 512 | 576 | 619 | 696 | 751 | 794 | 844 | 918 | 968 | 1013 | 1095 | 1122 | 1147 | 1173 | 1174 |  |
|  | PHRFP_4 | 31 | 47 | 47 | 47 | 47 | 47 | 47 | 47 | 47 | 47 | 47 | 47 | 47 | 47 | 47 | 47 | 47 | 47 | 47 | 47 | 47 | 47 | 47 | 47 | 47 | 47 | 47 | 47 | 47 | 47 | 47 | 1174 |  |
|  | PHRFP_3 | 31 | 45 | 57 | 79 | 87 | 90 | 92 | 94 | 97 | 108 | 116 | 143 | 148 | 158 | 208 | 248 | 251 | 251 | 251 | 251 | 251 | 251 | 251 | 251 | 251 | 251 | 251 | 251 | 251 | 251 | 251 | 1174 |  |
|  | PHRFP_2 | 37 | 42 | 53 | 56 | 65 | 70 | 78 | 82 | 96 | 112 | 122 | 125 | 131 | 141 | 147 | 182 | 244 | 324 | 373 | 465 | 606 | 683 | 761 | 806 | 870 | 962 | 1007 | 1056 | 1092 | 1109 | 1157 | 1174 |  |
|  | PHPFP_4 | 46 | 46 | 46 | 46 | 46 | 46 | 46 | 46 | 46 | 46 | 46 | 46 | 46 | 46 | 46 | 46 | 46 | 46 | 46 | 46 | 46 | 46 | 46 | 46 | 46 | 46 | 46 | 46 | 46 | 46 | 46 | 1174 |  |
|  | PHPFP_3 | 48 | 72 | 83 | 95 | 108 | 145 | 170 | 227 | 243 | 273 | 280 | 283 | 284 | 284 | 284 | 291 | 291 | 291 | 291 | 291 | 291 | 291 | 291 | 291 | 291 | 291 | 291 | 291 | 291 | 291 | 291 | 1174 |  |
|  | % Library | 1 | 2 | 3 | 4 | 5 | 6 | 7 | 8 | 9 | 10 | 11 | 12 | 13 | 14 | 15 | 20 | 25 | 30 | 35 | 40 | 45 | 50 | 55 | 60 | 65 | 70 | 75 | 80 | 85 | 90 | 95 | 100 |  |
| **Table S8.** Similarity analysis results for the active drug CHEMBL488890 (Melanin-concentrating hormone receptor 1) Cont. | PHPFP_2 | 38 | 62 | 82 | 104 | 130 | 148 | 158 | 164 | 173 | 177 | 197 | 207 | 217 | 238 | 248 | 352 | 427 | 478 | 546 | 607 | 655 | 745 | 817 | 875 | 899 | 975 | 1016 | 1061 | 1114 | 1128 | 1128 | 1174 | This table presents the number active compounds identified within a certain % of the CHEMBL library using 25 fingerprints. |
|  | PHFP_4 | 76 | 95 | 122 | 137 | 147 | 156 | 164 | 165 | 166 | 167 | 169 | 169 | 169 | 169 | 169 | 169 | 169 | 169 | 169 | 169 | 169 | 169 | 169 | 169 | 169 | 169 | 169 | 169 | 169 | 169 | 169 | 1174 |  |
|  | PHFP_3 | 61 | 83 | 98 | 118 | 129 | 141 | 147 | 156 | 169 | 190 | 200 | 205 | 215 | 239 | 251 | 354 | 395 | 445 | 501 | 552 | 610 | 656 | 720 | 807 | 902 | 928 | 928 | 928 | 928 | 928 | 928 | 1174 |  |
|  | PHFP_2 | 49 | 79 | 87 | 94 | 100 | 110 | 121 | 129 | 136 | 148 | 156 | 176 | 190 | 204 | 234 | 312 | 412 | 475 | 526 | 613 | 707 | 744 | 794 | 873 | 943 | 979 | 1009 | 1068 | 1118 | 1143 | 1173 | 1174 |  |
|  | MDL | 128 | 181 | 226 | 262 | 302 | 320 | 355 | 382 | 3938 | 428 | 460 | 479 | 491 | 516 | 544 | 639 | 746 | 820 | 873 | 925 | 971 | 1010 | 1064 | 1101 | 1120 | 1136 | 1155 | 1164 | 1170 | 1173 | 1173 | 1174 |  |
|  | ECFP_6 | 55 | 82 | 98 | 111 | 119 | 130 | 144 | 153 | 164 | 176 | 190 | 203 | 216 | 226 | 234 | 302 | 362 | 480 | 572 | 661 | 725 | 812 | 870 | 918 | 980 | 1031 | 1067 | 1098 | 1124 | 1153 | 1170 | 1174 |  |
|  | ECFP_4 | 61 | 92 | 106 | 113 | 128 | 136 | 147 | 161 | 177 | 184 | 199 | 210 | 220 | 228 | 249 | 324 | 398 | 510 | 596 | 675 | 741 | 805 | 875 | 927 | 980 | 1032 | 1075 | 1107 | 1126 | 1154 | 1170 | 1174 |  |
|  | ECFP_2 | 85 | 98 | 109 | 119 | 129 | 139 | 152 | 166 | 184 | 199 | 213 | 222 | 236 | 248 | 259 | 333 | 402 | 492 | 583 | 666 | 732 | 805 | 858 | 913 | 970 | 1005 | 1063 | 1107 | 1129 | 1163 | 1172 | 1174 |  |
|  | FCFP_6 | 38 | 65 | 80 | 94 | 99 | 105 | 111 | 121 | 131 | 142 | 147 | 157 | 169 | 175 | 191 | 232 | 313 | 376 | 433 | 503 | 553 | 601 | 662 | 746 | 834 | 903 | 966 | 1019 | 1079 | 1116 | 1153 | 1174 |  |
|  | FCFP_4 | 55 | 73 | 82 | 90 | 98 | 105 | 112 | 121 | 133 | 143 | 149 | 159 | 173 | 186 | 203 | 272 | 326 | 392 | 445 | 504 | 558 | 617 | 686 | 760 | 836 | 922 | 983 | 1034 | 1084 | 1117 | 1158 | 1174 |  |
|  | FCFP_2 | 25 | 45 | 68 | 79 | 85 | 95 | 111 | 124 | 127 | 141 | 157 | 162 | 172 | 182 | 188 | 256 | 317 | 387 | 459 | 515 | 591 | 688 | 745 | 806 | 873 | 940 | 1003 | 1049 | 1095 | 1129 | 1170 | 1174 |  |
|  | % Library | 1 | 2 | 3 | 4 | 5 | 6 | 7 | 8 | 9 | 10 | 11 | 12 | 13 | 14 | 15 | 20 | 25 | 30 | 35 | 40 | 45 | 50 | 55 | 60 | 65 | 70 | 75 | 80 | 85 | 90 | 95 | 100 |  |

| **Table S9.** Similarity analysis results for the active drug CHEMBL14759 (Human immunodeficiency virus type 1 protease) | PHRFC_4 | 171 | 171 | 171 | 171 | 171 | 171 | 171 | 171 | 171 | 171 | 171 | 171 | 171 | 171 | 171 | 171 | 171 | 171 | 171 | 171 | 171 | 171 | 171 | 171 | 171 | 171 | 171 | 171 | 171 | 171 | 171 | 1694 | This table presents the number active compounds identified within a certain % of the CHEMBL library using 25 fingerprints. |
| --- | --- | --- | --- | --- | --- | --- | --- | --- | --- | --- | --- | --- | --- | --- | --- | --- | --- | --- | --- | --- | --- | --- | --- | --- | --- | --- | --- | --- | --- | --- | --- | --- | --- | --- |
|  | PHRFC_3 | 169 | 196 | 205 | 214 | 222 | 231 | 246 | 259 | 270 | 274 | 279 | 290 | 292 | 303 | 313 | 343 | 343 | 343 | 343 | 343 | 343 | 343 | 343 | 343 | 343 | 343 | 343 | 343 | 343 | 343 | 343 | 1694 |  |
|  | PHRFC_2 | 158 | 168 | 172 | 180 | 193 | 202 | 220 | 230 | 241 | 273 | 288 | 299 | 305 | 313 | 326 | 411 | 477 | 586 | 663 | 720 | 773 | 849 | 920 | 1002 | 1113 | 1222 | 1351 | 1477 | 1562 | 1668 | 1668 | 1694 |  |
|  | PHPFC_4 | 166 | 166 | 166 | 166 | 166 | 166 | 166 | 166 | 166 | 166 | 166 | 166 | 166 | 166 | 166 | 166 | 166 | 166 | 166 | 166 | 166 | 166 | 166 | 166 | 166 | 166 | 166 | 166 | 166 | 166 | 166 | 1694 |  |
|  | PHPFC_3 | 228 | 243 | 270 | 272 | 272 | 272 | 272 | 272 | 272 | 272 | 272 | 272 | 272 | 272 | 272 | 272 | 272 | 272 | 272 | 272 | 272 | 272 | 272 | 272 | 272 | 272 | 272 | 272 | 272 | 272 | 272 | 1694 |  |
|  | PHPFC_2 | 234 | 266 | 274 | 283 | 302 | 307 | 315 | 319 | 320 | 327 | 330 | 344 | 348 | 356 | 361 | 440 | 500 | 605 | 735 | 920 | 920 | 920 | 920 | 920 | 920 | 920 | 920 | 920 | 920 | 920 | 920 | 1694 |  |
|  | PHFC_4 | 154 | 171 | 211 | 231 | 268 | 314 | 340 | 362 | 374 | 374 | 374 | 374 | 374 | 374 | 374 | 374 | 374 | 374 | 374 | 374 | 374 | 374 | 374 | 374 | 374 | 374 | 374 | 374 | 374 | 374 | 374 | 1694 |  |
|  | PHFC_3 | 156 | 202 | 235 | 248 | 265 | 284 | 295 | 307 | 316 | 322 | 340 | 348 | 356 | 366 | 379 | 479 | 587 | 688 | 798 | 941 | 1136 | 1394 | 1394 | 1394 | 1394 | 1394 | 1394 | 1394 | 1394 | 1394 | 1394 | 1694 |  |
|  | PHFC_2 | 131 | 151 | 164 | 178 | 191 | 200 | 207 | 236 | 270 | 279 | 288 | 298 | 308 | 324 | 335 | 380 | 420 | 503 | 566 | 641 | 738 | 797 | 891 | 974 | 1055 | 1150 | 1248 | 1353 | 1444 | 1585 | 1673 | 1694 |  |
|  | PHRFP_4 | 171 | 171 | 171 | 171 | 171 | 171 | 171 | 171 | 171 | 171 | 171 | 171 | 171 | 171 | 171 | 171 | 171 | 171 | 171 | 171 | 171 | 171 | 171 | 171 | 171 | 171 | 171 | 171 | 171 | 171 | 171 | 1694 |  |
|  | PHRFP_3 | 190 | 220 | 230 | 234 | 249 | 252 | 271 | 274 | 275 | 276 | 281 | 292 | 296 | 304 | 310 | 347 | 347 | 347 | 347 | 347 | 347 | 347 | 347 | 347 | 347 | 347 | 347 | 347 | 347 | 347 | 347 | 1694 |  |
|  | PHRFP_2 | 159 | 185 | 204 | 219 | 226 | 231 | 242 | 253 | 260 | 269 | 276 | 280 | 283 | 289 | 296 | 335 | 371 | 406 | 453 | 496 | 556 | 644 | 739 | 840 | 943 | 1051 | 1167 | 1306 | 1471 | 1668 | 1685 | 1694 |  |
|  | PHPFP_4 | 166 | 166 | 166 | 166 | 166 | 166 | 166 | 166 | 166 | 166 | 166 | 166 | 166 | 166 | 166 | 166 | 166 | 166 | 166 | 166 | 166 | 166 | 166 | 166 | 166 | 166 | 166 | 166 | 166 | 166 | 166 | 1694 |  |
|  | PHPFP_3 | 230 | 251 | 269 | 271 | 271 | 271 | 271 | 271 | 271 | 271 | 271 | 271 | 271 | 271 | 271 | 271 | 271 | 271 | 271 | 271 | 271 | 271 | 271 | 271 | 271 | 271 | 271 | 271 | 271 | 271 | 271 | 1694 |  |
|  | % Library | 1 | 2 | 3 | 4 | 5 | 6 | 7 | 8 | 9 | 10 | 11 | 12 | 13 | 14 | 15 | 20 | 25 | 30 | 35 | 40 | 45 | 50 | 55 | 60 | 65 | 70 | 75 | 80 | 85 | 90 | 95 | 100 |  |
| **Table S9.** Similarity analysis results for the active drug CHEMBL14759 (Human immunodeficiency virus type 1 protease) Cont. | PHPFP_2 | 240 | 273 | 296 | 303 | 317 | 326 | 338 | 346 | 352 | 363 | 374 | 381 | 416 | 420 | 427 | 471 | 539 | 651 | 790 | 917 | 917 | 917 | 917 | 917 | 917 | 917 | 917 | 917 | 917 | 917 | 917 | 1694 | This table presents the number active compounds identified within a certain % of the CHEMBL library using 25 fingerprints. |
|  | PHFP_4 | 159 | 176 | 212 | 232 | 251 | 265 | 307 | 368 | 373 | 373 | 373 | 373 | 373 | 373 | 373 | 373 | 373 | 373 | 373 | 373 | 373 | 373 | 373 | 373 | 373 | 373 | 373 | 373 | 373 | 373 | 373 | 1694 |  |
|  | PHFP_3 | 182 | 207 | 237 | 255 | 266 | 299 | 306 | 330 | 336 | 351 | 361 | 378 | 389 | 402 | 413 | 491 | 574 | 671 | 770 | 917 | 1097 | 1376 | 1376 | 1376 | 1376 | 1376 | 1376 | 1376 | 1376 | 1376 | 1376 | 1694 |  |
|  | PHFP_2 | 127 | 164 | 184 | 203 | 215 | 230 | 244 | 256 | 267 | 279 | 293 | 315 | 322 | 331 | 339 | 429 | 471 | 514 | 567 | 624 | 706 | 828 | 936 | 1052 | 1164 | 1268 | 1393 | 1483 | 1580 | 1663 | 1694 | 1694 |  |
|  | MDL | 168 | 203 | 227 | 234 | 241 | 246 | 261 | 272 | 275 | 282 | 292 | 297 | 300 | 303 | 307 | 335 | 364 | 401 | 462 | 503 | 583 | 649 | 750 | 854 | 1003 | 1129 | 1291 | 1416 | 1544 | 1615 | 168 | 1694 |  |
|  | ECFP_6 | 125 | 165 | 204 | 241 | 278 | 317 | 349 | 381 | 405 | 437 | 468 | 492 | 518 | 540 | 571 | 712 | 883 | 1065 | 1232 | 1336 | 1416 | 1493 | 1510 | 1529 | 1541 | 1556 | 1578 | 1600 | 1615 | 1652 | 1692 | 1694 |  |
|  | ECFP_4 | 124 | 164 | 188 | 223 | 250 | 278 | 290 | 308 | 333 | 355 | 386 | 407 | 417 | 435 | 452 | 541 | 626 | 712 | 813 | 932 | 1083 | 1209 | 1351 | 1429 | 1498 | 1549 | 1572 | 1591 | 1611 | 1658 | 1691 | 1694 |  |
|  | ECFP_2 | 151 | 182 | 206 | 229 | 240 | 246 | 250 | 260 | 264 | 272 | 278 | 285 | 296 | 305 | 315 | 362 | 410 | 443 | 483 | 539 | 606 | 684 | 755 | 828 | 928 | 1064 | 1209 | 1372 | 1519 | 1622 | 1687 | 1694 |  |
|  | FCFP_6 | 114 | 153 | 196 | 227 | 259 | 292 | 317 | 351 | 379 | 408 | 437 | 462 | 485 | 494 | 513 | 598 | 669 | 747 | 841 | 933 | 1033 | 1129 | 1252 | 1340 | 1445 | 1507 | 1557 | 1597 | 1628 | 1643 | 1681 | 1694 |  |
|  | FCFP_4 | 104 | 125 | 143 | 166 | 182 | 204 | 226 | 243 | 257 | 269 | 278 | 284 | 296 | 312 | 318 | 366 | 462 | 582 | 663 | 741 | 805 | 909 | 1011 | 1108 | 1203 | 1323 | 1415 | 1518 | 1594 | 1646 | 1679 | 1694 |  |
|  | FCFP_2 | 116 | 162 | 178 | 189 | 194 | 208 | 212 | 222 | 236 | 256 | 263 | 270 | 280 | 285 | 290 | 338 | 392 | 445 | 518 | 629 | 725 | 832 | 942 | 1038 | 1150 | 1283 | 1408 | 1491 | 1578 | 1667 | 1680 | 1694 |  |
|  | % Library | 1 | 2 | 3 | 4 | 5 | 6 | 7 | 8 | 9 | 10 | 11 | 12 | 13 | 14 | 15 | 20 | 25 | 30 | 35 | 40 | 45 | 50 | 55 | 60 | 65 | 70 | 75 | 80 | 85 | 90 | 95 | 100 |  |

**Table S10.** Substructures for promiscuous binders and HTS screening


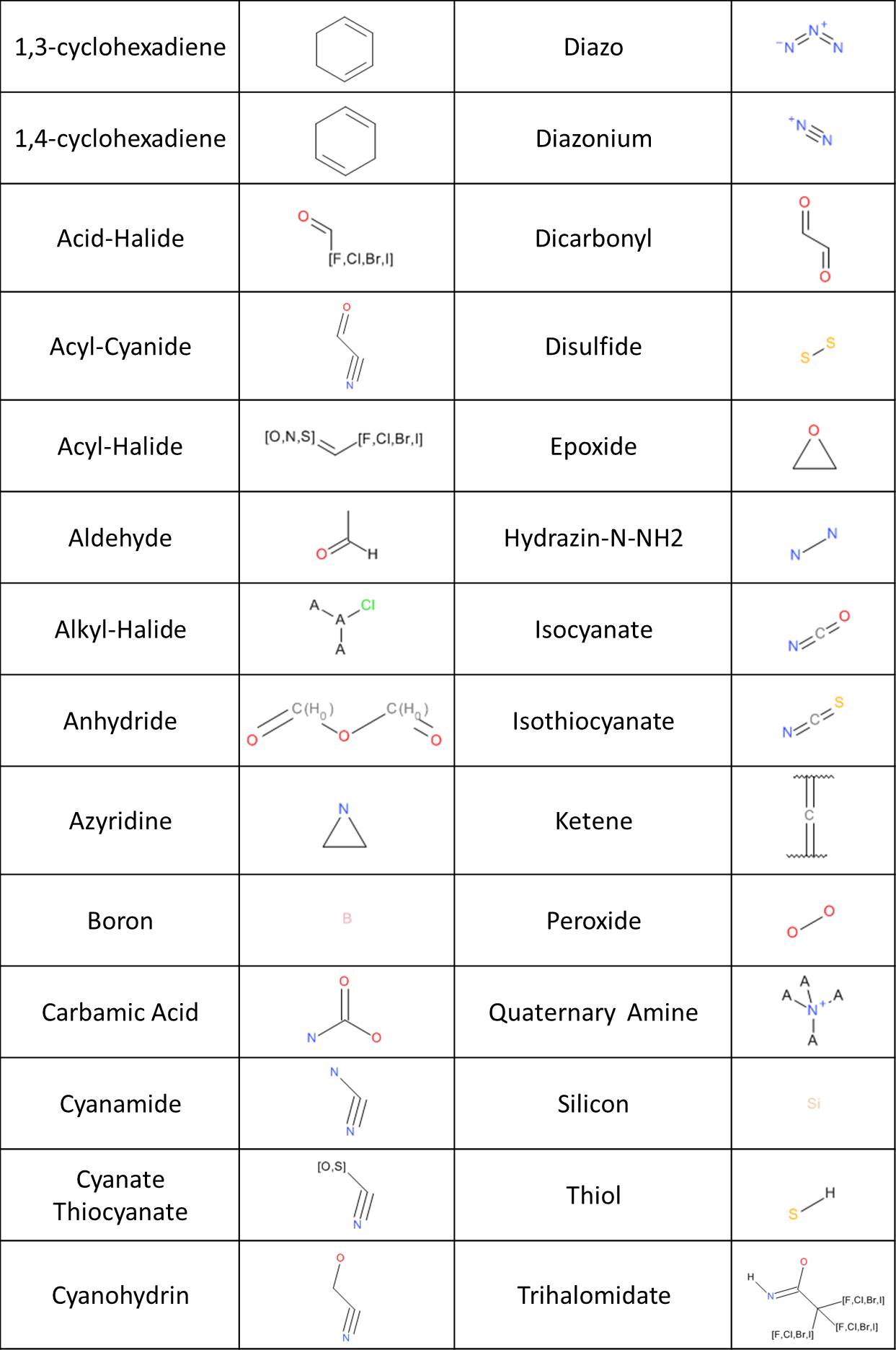


**Table S11.** Target Classification in the Drug Bank Database

| **Target** | **Size** | **Target** | **Size** |
| --- | --- | --- | --- |
| 1,3-beta-glucan synthase component FKS1 | 3 | Histamine H1 receptor | 39 |
| 23S rRNA | 3 | Histamine H2 receptor | 8 |
| 30S ribosomal protein S12 | 4 | HIV-1 protease | 10 |
| 3-hydroxy-3-methylglutaryl-coenzyme A reductase | 9 | Hypoxanthine-guanine phosphoribosyltransferase | 2 |
| 50S ribosomal protein L10 | 3 | Mineralocorticoid receptor | 4 |
| 5-hydroxytryptamine 1D receptor | 2 | Muscarinic acetylcholine receptor M1 | 10 |
| 5-hydroxytryptamine 3 receptor | 5 | Muscarinic acetylcholine receptor M3 | 7 |
| Acetylcholinesterase | 6 | Mu-type opioid receptor | 16 |
| Alpha-1A adrenergic receptor | 9 | Neuronal acetylcholine receptor subunit alpha-2 | 11 |
| Alpha-2A adrenergic receptor | 5 | P2Y purinoceptor 12 | 3 |
| Androgen receptor | 15 | Penicillin-binding protein 2 | 3 |
| Angiotensin-converting enzyme | 11 | Penicillin-binding protein 3 | 2 |
| Arachidonate 5-lipoxygenase | 3 | Penicillin-binding proteins 1A/1B | 10 |
| ATP-binding cassette transporter sub-family C member 8 | 8 | Peroxisome proliferator-activated receptor alpha | 3 |
| ATP-sensitive inward rectifier potassium channel 1 | 4 | Peroxisome proliferator-activated receptor gamma | 3 |
| Atrial natriuretic peptide receptor A | 4 | Potassium-transporting ATPase alpha chain 1 | 5 |
| bacterial outer membrane | 3 | Progesterone receptor | 4 |
| Beta-1 adrenergic receptor | 18 | Prostaglandin F2-alpha receptor | 4 |
| Beta-2 adrenergic receptor | 14 | Prostaglandin G/H synthase 2 | 20 |
| Calcium-activated potassium channel subunit alpha 1 | 2 | Prothrombin | 3 |
| Cannabinoid receptor 1 | 2 | Pyridoxal kinase | 2 |
| Carbonic anhydrase 2 | 2 | Renin | 2 |
| Catechol O-methyltransferase | 2 | Reverse transcriptase | 10 |
| cGMP-inhibited 3',5'-cyclic phosphodiesterase A | 5 | Serine/threonine-protein kinase mTOR | 3 |
| cGMP-specific 3',5'-cyclic phosphodiesterase | 5 | Sodium channel protein type 1 subunit alpha | 3 |
| Cholinesterase | 3 | Sodium channel protein type 10 subunit alpha | 9 |
| Coagulation factor X | 2 | Sodium channel protein type 5 subunit alpha | 17 |
| Cysteinyl leukotriene receptor 1 | 4 | Sodium/potassium-transporting ATPase alpha-1 chain | 7 |
| Cytochrome P450 19A1 | 5 | Sodium-dependent dopamine transporter | 4 |
| D(2) dopamine receptor | 10 | Sodium-dependent noradrenaline transporter | 7 |
| Dihydrofolate reductase | 3 | Sodium-dependent serotonin transporter | 9 |
| Dihydropteroate synthase | 7 | Solute carrier family 12 member 1 | 6 |
| Dihydropteroate synthetase | 6 | Solute carrier family 12 member 3 | 4 |
| Dipeptidyl peptidase 4 | 5 | Squalene monooxygenase | 4 |
| DNA | 37 | Synaptic vesicular amine transporter | 4 |
| DNA topoisomerase 2-alpha | 3 | Thymidylate synthase | 4 |
| Ergosterol, Candida albicans | 5 | Tubulin beta chain | 2 |
| Estrogen receptor | 13 | Type-1 angiotensin II receptor | 10 |
| Gamma-aminobutyric-acid receptor subunit alpha-1 | 4 | Vasopressin V2 receptor | 2 |
| Glucocorticoid receptor | 32 | Vitamin D3 receptor | 7 |
| Gonadotropin-releasing hormone receptor | 2 | Vitamin K epoxide reductase complex subunit 1 | 5 |
| Hepatitis C virus NS3-4A serine protease | 2 | Voltage-dependent calcium channel gamma-1 subunit | 2 |

The table lists all the targets remaining in the Drug Bank database after removing all singletons.

**Table S12.** Indication Classification in the CMC Database

| **Indication** | **Size** | **Indication** | **Size** |
| --- | --- | --- | --- |
| Adrenergic | 95 | Antitussive | 61 |
| Anabolic | 38 | Antiulcerative | 64 |
| Analeptic | 6 | Antiviral | 120 |
| Analgesic | 256 | Anxiolytic | 186 |
| Androgenic | 28 | Bone resorption inhibitor | 7 |
| Anesthetic | 118 | Bronchodilator | 58 |
| Antacid | 10 | Cardioprotectant | 10 |
| Anthelmintic | 116 | Cardiotonic | 82 |
| Antiacne | 7 | Choleretic | 37 |
| Antiallergic/Antiasthmatic | 84 | Cholinergic | 25 |
| Anti-Alzheimer's Disease | 9 | CNS stimulant | 59 |
| Antiamebic | 25 | Cognition enhancer | 12 |
| Antianemic | 6 | Contraceptive | 6 |
| Antianginal | 14 | Dental caries prophylactic | 10 |
| Antianorexic | 37 | Dermatologic | 23 |
| Antiarrhythmic | 85 | Detoxicant | 5 |
| Antiarthritic | 10 | Disinfectant | 28 |
| Antiatherosclerotic | 17 | Diuretic | 66 |
| Antibacterial | 27 | Ectoparasiticide | 7 |
| Antibiotic | 144 | Emetic | 6 |
| Anticholelithogenic | 7 | Erectile dysfunction agent | 5 |
| Anticholinergic | 17 | Estrogenic | 46 |
| Anticoagulant | 41 | Flavoring agent | 6 |
| Anticonvulsant | 83 | Gastric motility stimulant | 8 |
| Antidepressant | 204 | Gonad stimulator | 6 |
| Antidiabetic | 123 | Growth promoter | 16 |
| Antidiarrheal | 10 | Hematinic | 11 |
| Antiemetic | 34 | Hemostatic | 18 |
| Antiestrogen | 9 | Hepatoprotective | 12 |
| Antifungal | 151 | Hypocholesterolemic | 6 |
| Antiglaucoma | 13 | Hypolipidemic | 105 |
| Antihistamine | 96 | Laxative | 21 |
| Antihypertensive | 197 | Mucolytic | 33 |
| Antihypotensive | 5 | Multiple sclerosis (therapeutic) | 6 |
| Antiinfective | 40 | Muscle relaxant | 35 |
| Antiinflammatory | 230 | Narcotic | 14 |
| Antimalarial | 52 | Neuromuscular blocker | 13 |
| Antimigraine | 17 | NMDA antagonist | 7 |
| Antineoplastic | 654 | Progestin | 65 |
| Antiobesity | 14 | Prostaglandin | 5 |
| Antioxidant | 18 | Psychodysleptic | 7 |
| Antiparasitic | 9 | Respiratory stimulant | 11 |
| Antiparkinsonian | 35 | Secretory stimulant (gastric acid) | 5 |
| Antiperistaltic | 7 | Sedative/Hypnotic | 41 |
| Antiprotozoal | 37 | Spasmolytic | 37 |
| Antipruritic | 7 | Sweetener | 16 |
| Antipsychotic | 176 | Sympatholytic | 5 |
| Antipyretic | 6 | Sympathomimetic | 5 |
| Antirheumatic | 15 | Thyroid inhibitor | 7 |
| Antischistosomal | 14 | Uricosuric | 14 |
| Antisecretory (gastric) | 20 | Uterine stimulant | 11 |
| Antiseptic | 37 | Vasoconstrictor | 16 |
| Antithrombotic | 33 | Vasodilator | 56 |
| Antitrypanosomal | 7 |  |  |

The table lists all the indications remaining in the CMC database after removing singletons and doublets.

**Table S13.** Target Classification in the CHEMBL Database

| **Target** | **Size** | **Target** | **Size** |
| --- | --- | --- | --- |
| 11-beta-hydroxysteroid dehydrogenase 1 | 258 | Leukotriene B4 receptor 1 | 93 |
| 15-hydroxyprostaglandin dehydrogenase [NAD+] | 115 | Lignostilbene alpha, beta-dioxygenase | 6 |
| 1-acylglycerol-3-phosphate O-acyltransferase beta | 19 | LIM domain kinase 1 | 18 |
| 1-deoxy-D-xylulose-5-phosphate synthase | 18 | LIM domain kinase 2 | 23 |
| 1-deoxyxylulose-5-phosphate reductoisomerase | 14 | Lipoxin A4 receptor | 39 |
| 1-phosphatidylinositol-4,5-bisphosphate phosphodiesterase gamma-1 | 4 | Lipoxygenase | 4 |
| 3 beta-hydroxysteroid dehydrogenase/Delta 5-->4-isomerase | 5 | Liver glycogen phosphorylase | 101 |
| 3-dehydroquinate dehydratase | 27 | L-lactate dehydrogenase A chain | 11 |
| 3-hydroxy-3-methylglutaryl-coenzyme A reductase | 17 | Low affinity neurotrophin receptor p75NTR | 13 |
| 3-keto-steroid reductase | 46 | Low affinity sodium-glucose cotransporter | 5 |
| 3-oxoacyl-[acyl-carrier-protein] synthase 3 | 70 | Low molecular weight phosphotyrosine protein phosphatase | 21 |
| 3-phosphoinositide dependent protein kinase-1 | 120 | L-type amino acid transporter 1 | 3 |
| 4-hydroxyphenylpyruvate dioxygenase | 24 | Luciferase | 26 |
| 4'-phosphopantetheinyl transferase ffp | 103 | Luciferin 4-monooxygenase | 68 |
| 5-enolpyruvylshikimate-3-phosphate synthase | 6 | Luteinizing hormone/Choriogonadotropin receptor | 4 |
| 5-lipoxygenase activating protein | 6 | LXR-beta | 89 |
| 5'-methylthioadenosine/S-adenosylhomocysteine nucleosidase | 3 | Lysine-specific demethylase 4A | 56 |
| 5'-nucleotidase | 14 | Lysine-specific demethylase 4D-like | 30 |
| 6-O-methylguanine-DNA methyltransferase | 10 | Lysine-specific histone demethylase 1 | 103 |
| 6-phospho-1-fructokinase | 15 | Lysophosphatidic acid receptor 5 | 7 |
| 6-phosphofructo-2-kinase/fructose-2,6-bisphosphatase 3 | 15 | Lysophosphatidic acid receptor Edg-4 | 13 |
| 72 kDa type IV collagenase | 6 | Lysophosphatidic acid receptor Edg-7 | 3 |
| 78 kDa glucose-regulated protein | 10 | Lysosomal alpha-glucosidase | 14 |
| Aberrant vpr protein | 83 | Lysosomal protective protein | 23 |
| Accessory gene regulator protein A | 3 | Lysosomal Pro-X carboxypeptidase | 141 |
| Acetolactate synthase | 30 | Lysyl-tRNA synthetase | 13 |
| Acetylcholine receptor protein delta chain | 6 | Macrophage colony stimulating factor receptor | 103 |
| Acetylcholine-binding protein | 8 | Macrophage migration inhibitory factor | 109 |
| Acetylcholinesterase | 1186 | Macrophage migration inhibitory factor homologue | 42 |
| Acetyl-CoA carboxylase 1 | 4 | Major prion protein | 5 |
| Acetyl-CoA carboxylase 2 | 5 | Malonyl-CoA decarboxylase | 147 |
| Acid ceramidase | 7 | Mannose-6-phosphate isomerase | 23 |
| Acidic alpha-glucosidase | 6 | MAP kinase ERK2 | 21 |
| Acidic mammalian chitinase | 5 | MAP kinase p38 | 82 |
| Acrosin | 161 | MAP kinase p38 alpha | 777 |
| Acyl coenzyme A:cholesterol acyltransferase 1 | 316 | MAP kinase p38 delta | 3 |
| Acyl-CoA desaturase | 36 | MAP kinase-activated protein kinase 2 | 423 |
| Acyl-CoA desaturase 1 | 186 | MAP kinase-interacting serine/threonine-protein kinase MNK1 | 11 |
| Acyl-CoA:cholesterol acyltransferase | 194 | Mas-related G-protein coupled receptor member X1 | 11 |
| ADAM17 | 311 | Mast cell carboxypeptidase A | 3 |
| ADAMTS4 | 58 | Matriptase | 10 |
| ADAMTS5 | 235 | Matrix metalloproteinase 12 | 35 |
| Adenosine A1 receptor | 176 | Matrix metalloproteinase 13 | 106 |
| Adenosine A2a receptor | 129 | Matrix metalloproteinase 14 | 18 |
| Adenosine A2b receptor | 33 | Matrix metalloproteinase 3 | 106 |
| Adenosine A3 receptor | 72 | Matrix metalloproteinase 7 | 15 |
| Adenosine deaminase | 43 | Matrix metalloproteinase 8 | 103 |
| Adenosine kinase | 172 | Matrix metalloproteinase 9 | 58 |
| Adenosylmethionine-8-amino-7-oxononanoate aminotransferase | 71 | Matrix metalloproteinase-1 | 190 |
| Adenylate cyclase type V | 4 | Matrix metalloproteinase-2 | 41 |
| ADM | 3 | Melanin-concentrating hormone receptor 1 | 1182 |
| Adrenergic receptor alpha | 26 | Melanin-concentrating hormone receptor 2 | 23 |
| Adrenergic receptor alpha-1 | 49 | Melanocortin receptor 1 | 10 |
| Adrenergic receptor alpha-2 | 6 | Melanocortin receptor 4 | 523 |
| Adrenergic receptor beta | 58 | Melanocortin receptor 5 | 14 |
| Adrenomedullin receptor | 13 | Melatonin receptor | 64 |
| Advanced glycosylation end product-specific receptor | 45 | Melatonin receptor 1A | 98 |
| AICAR transformylase | 37 | Melatonin receptor 1B | 21 |
| Alcohol dehydrogenase | 8 | Membrane-associated guanylate kinase-related 3 | 4 |
| Aldehyde dehydrogenase 1A1 | 86 | Membrane-bound transcription factor site-1 protease | 31 |
| Aldehyde oxidase | 9 | Menin | 21 |
| Aldo-keto-reductase family 1 member C3 | 60 | Metabotropic glutamate receptor 1 | 109 |
| Aldose reductase | 696 | Metabotropic glutamate receptor 2 | 240 |
| ALK tyrosine kinase receptor | 48 | Metabotropic glutamate receptor 3 | 12 |
| Alkaline phosphatase tissue-nonspecific | 54 | Metabotropic glutamate receptor 4 | 43 |
| Alkaline phosphatase, tissue-nonspecific isozyme | 19 | Metabotropic glutamate receptor 5 | 911 |
| Allene oxide synthase, chloroplastic | 11 | Metabotropic glutamate receptor 7 | 15 |
| Alpha-1,6-mannosyl-glycoprotein 2-beta-N-acetylglucosaminyltransferase | 8 | Metastin receptor | 26 |
| Alpha-1a adrenergic receptor | 48 | Methionine aminopeptidase 2 | 95 |
| Alpha-1-acid glycoprotein 1 | 3 | Methionyl-tRNA synthetase | 31 |
| Alpha-1d adrenergic receptor | 15 | MHC class II | 11 |
| Alpha-2c adrenergic receptor | 8 | Microbial collagenase | 203 |
| Alpha-chymotrypsin | 6 | Microsomal triglyceride transfer protein large subunit | 110 |
| Alpha-galactosidase A | 10 | Microtubule-associated protein tau | 147 |
| Alpha-glucosidase MAL12 | 14 | Mineralocorticoid receptor | 200 |
| Alpha-glucosidase MAL62 | 19 | Mitochondrial complex I; NADH oxidoreductase | 7 |
| Alpha-ketoglutarate-dependent dioxygenase alkB homolog 3 | 25 | Mitogen-activated protein kinase 7 | 3 |
| Alpha-L-fucosidase I | 9 | Mitogen-activated protein kinase kinase kinase 14 | 24 |
| Alpha-synuclein | 120 | Mitogen-activated protein kinase kinase kinase 5 | 51 |
| Amiloride-sensitive cation channel 3 | 40 | Mitogen-activated protein kinase kinase kinase 8 | 113 |
| Amine oxidase, copper containing | 16 | Mitogen-activated protein kinase kinase kinase kinase 4 | 15 |
| Aminopeptidase B | 4 | Mitotic checkpoint serine/threonine-protein kinase BUB1 | 6 |
| Aminopeptidase N | 160 | Moloney murine leukaemia virus Pol protein | 30 |
| AMP-activated protein kinase, alpha-1 subunit | 36 | Monoamine oxidase | 30 |
| AMP-activated protein kinase, alpha-2 subunit | 5 | Monoamine oxidase A | 101 |
| AMP-activated protein kinase, AMPK | 5 | Monoamine oxidase B | 47 |
| Anandamide amidohydrolase | 676 | Monoglyceride lipase | 11 |
| Androgen Receptor | 319 | Mothers against decapentaplegic homolog 3 | 274 |
| Angiotensin II receptor | 381 | Motilin receptor | 144 |
| Angiotensin II receptor (AT-1) type-1 | 112 | Mu opioid receptor | 146 |
| Angiotensin II type 1a (AT-1a) receptor | 366 | Multidrug resistance-associated protein 1 | 43 |
| Angiotensin II type 2 (AT-2) receptor | 20 | Multidrug resistance-associated protein 5 | 6 |
| Angiotensin-converting enzyme | 433 | Multidrug resistance-associated protein 7 | 3 |
| Angiotensin-converting enzyme 2 | 16 | Multidrug translocase mdfA | 26 |
| Anoctamin-1 | 29 | Muscarinic acetylcholine receptor | 30 |
| Anthrax lethal factor | 218 | Muscarinic acetylcholine receptor M1 | 334 |
| Anti-estrogen binding site (AEBS) | 6 | Muscarinic acetylcholine receptor M2 | 39 |
| Antrax lethal toxin | 41 | Muscarinic acetylcholine receptor M3 | 114 |
| Apoptosis regulator Bcl-2 | 53 | Muscarinic acetylcholine receptor M4 | 44 |
| Apoptosis regulator Bcl-W | 3 | Muscarinic acetylcholine receptor M5 | 49 |
| Apoptosis regulator Bcl-X | 135 | Muscle glycogen phosphorylase | 27 |
| Apoptotic protease-activating factor 1 | 66 | Myc proto-oncogene protein | 16 |
| Arabinose 5-phosphate isomerase | 4 | Mycophenolic acid acyl-glucuronide esterase, mitochondrial | 4 |
| Arachidonate 12-lipoxygenase | 51 | Mycothiol S-conjugate amidase | 19 |
| Arachidonate 15-lipoxygenase | 81 | Myelin basic protein | 11 |
| Arachidonate 5-lipoxygenase | 516 | Myosin light chain kinase, smooth muscle | 58 |
| Arginase | 6 | NAALADase II | 6 |
| Aryl hydrocarbon receptor | 93 | N-acylethanolamine-hydrolyzing acid amidase | 30 |
| Arylamine N-acetyltransferase 2 | 22 | N-acylsphingosine-amidohydrolase | 15 |
| Asialoglycoprotein receptor 1 | 5 | NAD-dependent deacetylase HST2 | 6 |
| Ataxin-2 | 359 | NAD-dependent deacetylase sirtuin 1 | 82 |
| ATPase family AAA domain-containing protein 5 | 144 | NAD-dependent deacetylase sirtuin 2 | 257 |
| ATP-binding cassette sub-family G member 2 | 74 | NAD-dependent deacetylase sirtuin 3 | 17 |
| ATP-citrate synthase | 95 | NAD-dependent histone deacetylase SIR2 | 21 |
| ATP-dependent Clp protease proteolytic subunit | 92 | NAD-dependent protein deacetylase sirtuin-6 | 13 |
| ATP-dependent RNA helicase DDX3X | 19 | NADP-dependent malic enzyme | 13 |
| ATP-sensitive inward rectifier potassium channel 1 | 20 | NADPH oxidase 1 | 98 |
| Autoinducer 1 sensor kinase/phosphatase luxN | 5 | NADPH oxidase 4 | 42 |
| Autotaxin | 452 | Neprilysin | 154 |
| Avian myoblastosis virus polyprotein II | 17 | Nerve growth factor receptor Trk-A | 52 |
| B1 bradykinin receptor | 40 | Neuraminidase | 3 |
| Baculoviral IAP repeat-containing protein 2 | 8 | Neurokinin 1 receptor | 1170 |
| Baculoviral IAP repeat-containing protein 5 | 24 | Neurokinin 2 receptor | 235 |
| Bcl2-antagonist of cell death (BAD) | 10 | Neurokinin 3 receptor | 193 |
| Bcr/Abl fusion protein | 15 | Neuromedin B receptor | 234 |
| BDNF/NT-3 growth factors receptor | 9 | Neuronal acetylcholine receptor | 57 |
| Beta amyloid A4 protein | 110 | Neuronal acetylcholine receptor protein alpha-10 subunit | 46 |
| Beta tubulin | 6 | Neuronal acetylcholine receptor protein alpha-7 subunit | 210 |
| Beta-1 adrenergic receptor | 26 | Neuronal acetylcholine receptor protein alpha-9 subunit | 7 |
| Beta-1,4-galactosyltransferase 1 | 5 | Neuronal acetylcholine receptor; alpha3/beta4 | 9 |
| Beta-2 adrenergic receptor | 48 | Neuronal acetylcholine receptor; alpha4/beta2 | 91 |
| Beta-3 adrenergic receptor | 162 | Neuropeptide FF receptor 2 | 3 |
| Beta-adrenergic receptor | 18 | Neuropeptide S receptor | 29 |
| Beta-chymotrypsin | 20 | Neuropeptide Y receptor type 1 | 361 |
| Beta-glucocerebrosidase | 65 | Neuropeptide Y receptor type 2 | 126 |
| Beta-glucosidase | 7 | Neuropeptide Y receptor type 5 | 613 |
| Beta-glucuronidase | 15 | Neuropeptides B/W receptor type 1 | 74 |
| Beta-ketoacyl-ACP synthase III | 8 | Neuropilin-1 | 85 |
| Beta-lactamase | 50 | Neurotensin receptor 1 | 29 |
| Beta-lactamase AmpC | 421 | Neurotensin receptor 3 | 30 |
| Beta-lactamase L1 | 69 | Neutral cholesterol ester hydrolase 1 | 26 |
| Beta-lactamase OXA-9 | 6 | NH(3)-dependent NAD(+) synthetase | 7 |
| Beta-lactamase TEM | 21 | Nicotinamide phosphoribosyltransferase | 12 |
| Beta-N-acetyl-D-hexosaminidase-A/B | 14 | Nicotinate-nucleotide adenylyltransferase | 8 |
| Beta-secretase (BACE) | 12 | Nicotinic acetylcholine receptor alpha8 subunit | 9 |
| Beta-secretase 1 | 1056 | Niemann-Pick C1-like protein 1 | 106 |
| Bile acid receptor | 3 | Nischarin | 63 |
| Bile acid receptor FXR | 171 | Nitric oxide synthase, inducible | 51 |
| Bile salt export pump | 4 | Nitric-oxide synthase, brain | 109 |
| Bloom syndrome protein | 46 | Nitric-oxide synthase, endothelial | 26 |
| Bombesin receptor subtype-3 | 12 | Nociceptin receptor | 184 |
| Bone morphogenetic protein 1 | 294 | Nonstructural protein 1 | 46 |
| Bone morphogenetic protein 4 | 42 | Norepinephrine transporter | 21 |
| Botulinum neurotoxin type A | 157 | Nuclear factor erythroid 2-related factor 2 | 232 |
| Bradykinin B1 receptor | 211 | Nuclear factor NF-kappa-B complex | 16 |
| Bradykinin B2 receptor | 146 | Nuclear factor NF-kappa-B p105 subunit | 56 |
| Brain glycogen phosphorylase | 10 | Nuclear receptor ROR-gamma | 44 |
| Branched-chain-amino-acid transferase | 25 | Nuclear receptor subfamily 4 group A member 1 | 35 |
| Breast cancer type 1 susceptibility protein | 55 | O-acetylserine sulfhydrylase | 20 |
| Bromodomain adjacent to zinc finger domain protein 2B | 59 | Oligopeptide transporter small intestine isoform | 50 |
| Bromodomain-containing protein 4 | 53 | Oligosaccharyl transferase 48 kDa subunit | 6 |
| Butyrylcholinesterase | 26 | Opioid receptor | 68 |
| C3a anaphylatoxin chemotactic receptor | 54 | Opioid receptors; mu/kappa/delta | 3 |
| C5a anaphylatoxin chemotactic receptor | 139 | Orexin receptor 1 | 12 |
| Calcitonin gene-related peptide type 1 receptor | 160 | Ornithine decarboxylase | 18 |
| Calcitonin-gene-related peptide receptor, CALCRL/RAMP1 | 16 | Oxoeicosanoid receptor 1 | 25 |
| Calcium sensing receptor | 307 | Oxysterols receptor LXR-beta | 29 |
| Calcium-activated potassium channel subunit alpha-1 | 233 | Oxytocin receptor | 152 |
| Calcium-independent phospholipase A2 | 3 | P2X purinoceptor 1 | 3 |
| Calmodulin-sensitive adenylate cyclase | 6 | P2X purinoceptor 3 | 24 |
| Calpain 1 | 335 | P2X purinoceptor 7 | 1041 |
| Calpain 2 | 13 | p53-binding protein Mdm-2 | 229 |
| Calpain small subunit 1 | 22 | Paired box protein Pax-8 | 173 |
| CaM kinase II delta | 83 | Pancreatic elastase | 8 |
| cAMP and cAMP-inhibited cGMP 3',5'-cyclic phosphodiesterase 10A | 114 | Pancreatic triacylglycerol lipase | 9 |
| cAMP-dependent protein kinase alpha-catalytic subunit | 4 | Pantothenate synthetase | 9 |
| Canalicular multispecific organic anion transporter 1 | 27 | Papain | 45 |
| Candidapepsin-2 | 20 | Parathyroid hormone receptor | 28 |
| Cannabinoid CB1 receptor | 965 | Penicillopepsin | 3 |
| Cannabinoid CB2 receptor | 191 | Pepsin A | 10 |
| Cannabinoid receptor | 5 | Peptide deformylase | 34 |
| Carbonic anhydrase | 5 | Peptide deformylase mitochondrial | 4 |
| Carbonic anhydrase II | 223 | Peptide N-myristoyltransferase | 7 |
| Carboxylesterase | 3 | Peptide N-myristoyltransferase 1 | 7 |
| Carboxypeptidase A1 | 61 | Peptidyl-glycine alpha-amidating monooxygenase | 21 |
| Casein kinase I alpha | 4 | Peptidyl-prolyl cis-trans isomerase FKBP5 | 17 |
| Casein kinase I delta | 21 | Peptidyl-prolyl cis-trans isomerase NIMA-interacting 1 | 179 |
| Casein kinase II | 28 | Perforin-1 | 134 |
| Casein kinase II alpha | 30 | Peripheral myelin protein 22 | 10 |
| Caspase-1 | 305 | Peripheral-type benzodiazepine receptor | 194 |
| Caspase-3 | 279 | Peroxisome proliferator-activated receptor alpha | 75 |
| Catechol O-methyltransferase | 85 | Peroxisome proliferator-activated receptor delta | 48 |
| Catenin beta-1 | 3 | Peroxisome proliferator-activated receptor gamma | 448 |
| Cathepsin B | 70 | P-glycoprotein 1 | 540 |
| Cathepsin D | 69 | Phenylalanyl-tRNA synthetase alpha chain | 33 |
| Cathepsin G | 14 | Phenylethanolamine N-methyltransferase | 77 |
| Cathepsin K | 285 | Phosphatidylinositol 3,4,5-trisphosphate 5-phosphatase 2 | 12 |
| Cathepsin L | 39 | Phosphatidylinositol 4-kinase, PI4K | 5 |
| Cathepsin S | 162 | Phosphatidylinositol-3,4,5-trisphosphate 5-phosphatase 1 | 3 |
| C-C chemokine receptor type 1 | 250 | Phosphodiesterase 1 | 14 |
| C-C chemokine receptor type 2 | 472 | Phosphodiesterase 10A | 271 |
| C-C chemokine receptor type 3 | 567 | Phosphodiesterase 2A | 17 |
| C-C chemokine receptor type 4 | 213 | Phosphodiesterase 3 | 31 |
| C-C chemokine receptor type 5 | 482 | Phosphodiesterase 4 | 69 |
| C-C chemokine receptor type 8 | 127 | Phosphodiesterase 4A | 74 |
| C-C motif chemokine 2 | 10 | Phosphodiesterase 4B | 308 |
| Cdc2-related kinase | 6 | Phosphodiesterase 4D | 58 |
| CDK2/Cyclin A | 37 | Phosphodiesterase 5A | 363 |
| CDK9/cyclin T1 | 72 | Phosphodiesterase 6D | 27 |
| Cell division cycle 7-related protein kinase | 43 | Phosphodiesterase 7A | 110 |
| Cellular tumor antigen p53 | 143 | Phosphodiesterase 8B | 35 |
| Centromere-associated protein E | 12 | Phosphodiesterase 9A | 12 |
| Ceramide glucosyltransferase | 19 | Phosphoenolpyruvate carboxykinase cytosolic | 9 |
| cGMP-dependent protein kinase | 8 | Phosphoglycerate kinase | 89 |
| Chaperone protein dnaK | 5 | Phospholipase A2 group 1B | 65 |
| Chitin synthase 1 | 5 | Phospholipase A2 group IIA | 75 |
| Chitinase | 6 | Phospholipase A2 group IIC | 4 |
| Chitinase B | 5 | Phospholipase A2 group V | 7 |
| Cholecystokinin A receptor | 89 | Phospholipase A2 isozyme CM-I | 9 |
| Cholecystokinin B receptor | 71 | Phospholipase D2 | 10 |
| Cholecystokinin receptor | 26 | Phosphotyrosine-protein phosphatase PTPB | 10 |
| Cholesteryl ester transfer protein | 445 | Photosystem Q(B) protein | 4 |
| Choline acetylase | 30 | PI3-kinase p110-alpha subunit | 106 |
| Cholinesterase | 6 | PI3-kinase p110-alpha/p85-alpha | 20 |
| Chondroitin sulfate N-acetylgalactosaminyltransferase 1 | 23 | PI3-kinase p110-beta subunit | 5 |
| Chorismate mutase-related protein | 12 | PI3-kinase p110-delta subunit | 22 |
| Chorismate synthase | 41 | PI3-kinase p110-gamma subunit | 43 |
| Chromobox protein homolog 1 | 595 | PI4-kinase alpha subunit | 17 |
| Chromobox protein homolog 7 | 53 | Pituitary adenylate cyclase-activating polypeptide type I receptor | 29 |
| Chymase | 151 | Plasma retinol-binding protein | 49 |
| c-Jun N-terminal kinase 1 | 137 | Plasmepsin 2 | 14 |
| c-Jun N-terminal kinase 3 | 103 | Plasminogen | 3 |
| Class 1 phosphodiesterase PDEB1 | 44 | Plasminogen activator inhibitor-1 | 212 |
| Clathrin heavy chain 1 | 13 | Platelet activating factor receptor | 291 |
| Coagulation factor III | 14 | Platelet-activating factor acetylhydrolase | 3 |
| Coagulation factor VII | 25 | Platelet-derived growth factor receptor | 158 |
| Coagulation factor VII/tissue factor | 19 | Platelet-derived growth factor receptor beta | 161 |
| Coagulation factor X | 1147 | Poly [ADP-ribose] polymerase-1 | 542 |
| Coagulation factor XI | 22 | Polyamine oxidase | 5 |
| Coagulation factor XIII | 7 | Polymerase acidic protein | 48 |
| Collagen-binding protein 1 | 4 | Potassium channel, inwardly rectifying, subfamily J, member 11 | 66 |
| Complement C1r | 21 | Potassium-transporting ATPase | 80 |
| Complement C1s | 55 | Pregnane X receptor | 32 |
| Complement factor D | 5 | Prelamin-A/C | 96 |
| Corticotropin releasing factor receptor | 60 | Prenyl protein specific protease | 12 |
| Corticotropin releasing factor receptor 1 | 1344 | Prion protein | 9 |
| CpG DNA methylase | 8 | Probable G-protein coupled receptor 142 | 3 |
| Creatine kinase M | 4 | Probable low molecular weight protein-tyrosine-phosphatase | 45 |
| Creatine kinase M-type | 3 | Probable protein-cysteine N-palmitoyltransferase porcupine | 55 |
| Creatine transporter | 3 | Progesterone receptor | 345 |
| Cruzipain | 223 | Prokineticin receptor 1 | 22 |
| C-X-C chemokine receptor type 3 | 363 | Proliferating cell nuclear antigen | 7 |
| C-X-C chemokine receptor type 4 | 67 | Prolyl 4-hydroxylase alpha-1 subunit | 9 |
| C-X-C chemokine receptor type 7 | 42 | Prolyl endopeptidase | 174 |
| Cyclin-dependent kinase 1 | 159 | Prostaglandin E synthase | 196 |
| Cyclin-dependent kinase 1/cyclin B | 13 | Prostaglandin E synthase 2 | 14 |
| Cyclin-dependent kinase 1/cyclin B1 | 7 | Prostanoid DP receptor | 65 |
| Cyclin-dependent kinase 2 | 84 | Prostanoid EP1 receptor | 257 |
| Cyclin-dependent kinase 2/cyclin A | 103 | Prostanoid EP2 receptor | 74 |
| Cyclin-dependent kinase 2/cyclin E | 80 | Prostanoid EP3 receptor | 147 |
| Cyclin-dependent kinase 4 | 9 | Prostanoid EP4 receptor | 41 |
| Cyclin-dependent kinase 4/cyclin D | 7 | Prostanoid FP receptor | 9 |
| Cyclin-dependent kinase 4/cyclin D1 | 81 | Prostanoid IP receptor | 51 |
| Cyclin-dependent kinase 5/CDK5 activator 1 | 171 | Prostasin | 22 |
| Cyclooxygenase | 30 | Prostate specific antigen | 49 |
| Cyclooxygenase-1 | 62 | Prostatic acid phosphatase | 30 |
| Cyclooxygenase-2 | 135 | Protease | 56 |
| Cyclophilin A | 67 | Proteasome Macropain subunit MB1 | 14 |
| Cystathionine beta-lyase metC | 4 | Protein arginine N-methyltransferase 3 | 46 |
| Cysteine protease | 4 | Protein disulfide-isomerase | 6 |
| Cysteinyl leukotriene receptor | 267 | Protein E6 | 3 |
| Cysteinyl leukotriene receptor 1 | 205 | Protein farnesyltransferase | 606 |
| Cystic fibrosis transmembrane conductance regulator | 124 | Protein farnesyltransferase (PFT) | 6 |
| Cystine/glutamate transporter | 29 | Protein HEXIM1 | 3 |
| Cystinyl aminopeptidase | 36 | Protein kinase C (PKC) | 86 |
| Cytochrome b | 3 | Protein kinase C alpha | 230 |
| Cytochrome b6-f complex subunit 4 | 9 | Protein kinase C delta | 5 |
| Cytochrome P450 11A1 | 3 | Protein kinase C epsilon | 19 |
| Cytochrome P450 11B1 | 4 | Protein kinase C gamma | 50 |
| Cytochrome P450 11B2 | 7 | Protein kinase C theta | 99 |
| Cytochrome P450 17A1 | 62 | Protein kinase C zeta | 48 |
| Cytochrome P450 19A1 | 447 | Protein kinase Pfmrk | 47 |
| Cytochrome P450 24A1 | 20 | Protein Mdm4 | 18 |
| Cytochrome P450 26A1 | 46 | Protein phosphatase methylesterase 1 | 4 |
| Cytochrome P450 2A6 | 5 | Protein prune homolog | 4 |
| Cytochrome P450 2B1 | 4 | Protein skinhead-1 | 4 |
| Cytochrome P450 2C19 | 4 | Protein tyrosine kinase 2 beta | 67 |
| Cytochrome P450 2C9 | 100 | Protein-arginine deiminase type-4 | 3 |
| Cytochrome P450 2D6 | 57 | Protein-arginine N-methyltransferase 1 | 44 |
| Cytochrome P450 2J2 | 11 | Proteinase-activated receptor 1 | 317 |
| Cytochrome P450 3A4 | 73 | Proteinase-activated receptor 2 | 13 |
| Cytochrome P450 4F2 | 3 | Protein-glutamine gamma-glutamyltransferase | 87 |
| Cytochrome P450 51 | 65 | Protein-tyrosine phosphatase 1B | 1018 |
| Cytochrome P450 monooxygenase | 8 | Protein-tyrosine phosphatase 2C | 42 |
| Cytosolic phospholipase A2 | 44 | Protein-tyrosine phosphatase 4A3 | 20 |
| D-amino-acid oxidase | 38 | Protein-tyrosine phosphatase LC-PTP | 30 |
| Delta opioid receptor | 44 | Protein-tyrosine sulfotransferase 2 | 14 |
| Dengue virus type 2 NS3 protein | 14 | Proto-oncogene c-JUN | 3 |
| Deoxycytidine kinase | 26 | Proto-oncogene protein Wnt-3 | 42 |
| Diacylglycerol O-acyltransferase 1 | 144 | Proto-oncogene tyrosine-protein kinase ROS | 37 |
| Dihydrodipicolinate synthase-like, mitochondrial | 4 | Protoporphyrinogen oxidase | 42 |
| Dihydrofolate reductase | 516 | P-selectin | 86 |
| Dihydrofolate reductase type 1 | 5 | P-selectin glycoprotein ligand 1 | 54 |
| Dihydroorotate dehydrogenase | 157 | Pseudolysin | 58 |
| Dihydropteroate synthase | 34 | Pteridine reductase 1 | 30 |
| Dipeptidyl peptidase I | 26 | Pteridine reductase, putative | 19 |
| Dipeptidyl peptidase IV | 618 | Purine nucleoside phosphorylase | 38 |
| Diphosphomevalonate decarboxylase | 26 | Purinergic receptor P2Y1 | 115 |
| Disks large homolog 4 | 10 | Purinergic receptor P2Y12 | 82 |
| DNA (cytosine-5)-methyltransferase 1 | 41 | Purinergic receptor P2Y2 | 21 |
| DNA dC->dU-editing enzyme APOBEC-3F | 20 | Puromycin-sensitive aminopeptidase | 4 |
| DNA dC->dU-editing enzyme APOBEC-3G | 5 | Putative fructose-1,6-bisphosphate aldolase | 8 |
| DNA gyrase | 50 | Putative uncharacterized protein | 5 |
| DNA ligase | 4 | Putative uncharacterized protein pk7 | 17 |
| DNA polymerase beta | 84 | Pyridoxine-5'-phosphate oxidase | 3 |
| DNA polymerase eta | 20 | Pyroglutamylated RFamide peptide receptor | 4 |
| DNA polymerase iota | 809 | Pyruvate dehydrogenase kinase | 99 |
| DNA polymerase kappa | 27 | Pyruvate kinase | 36 |
| DNA repair protein RAD52 homolog | 9 | Pyruvate kinase isozymes M1/M2 | 38 |
| DNA topoisomerase I | 196 | Quinolone resistance protein norA | 46 |
| DNA topoisomerase II | 136 | Quinone oxidoreductase | 9 |
| DNA topoisomerase II alpha | 41 | Quinone reductase 1) | 35 |
| DNA topoisomerase III | 17 | Quinone reductase 2 | 30 |
| DNA-(apurinic or apyrimidinic site) lyase | 118 | Ras-related C3 botulinum toxin substrate 1 | 42 |
| DNA-dependent protein kinase | 429 | Receptor protein-tyrosine kinase erbB-2 | 34 |
| DOPA decarboxylase | 4 | Receptor-type tyrosine-protein phosphatase beta | 5 |
| Dopamine beta-hydroxylase | 52 | Receptor-type tyrosine-protein phosphatase F (LAR) | 5 |
| Dopamine D1 receptor | 34 | Regulator of G-protein signaling 4 | 15 |
| Dopamine D2 receptor | 197 | Relaxin receptor 1 | 39 |
| Dopamine D3 receptor | 75 | Renal dipeptidase | 44 |
| Dopamine D4 receptor | 72 | Renal sodium-dependent phosphate transport protein 1 | 3 |
| Dopamine receptor | 59 | Renin | 848 |
| Dopamine receptors; D2 & D3 | 3 | Replication protein A 70 kDa DNA-binding subunit | 47 |
| Dopamine transporter | 326 | Replication protein E1 | 18 |
| dTDP-4-dehydrorhamnose reductase | 7 | Retinoic acid receptor gamma | 3 |
| Dual specificity mitogen-activated protein kinase kinase 1 | 101 | Retinoid X receptor alpha | 72 |
| Dual specificity mitogen-activated protein kinase kinase; MEK1/2 | 12 | Reverse transcriptase | 5 |
| Dual specificity phosphatase 22 | 27 | Rho-associated protein kinase 1 | 38 |
| Dual specificity phosphatase Cdc25A | 23 | Rho-associated protein kinase 2 | 42 |
| Dual specificity phosphatase Cdc25B | 108 | Ribonuclease pancreatic | 4 |
| Dual specificity phosphatase Cdc25C | 14 | Ribonucleoside-diphosphate reductase RR1 | 15 |
| Dual specificity protein kinase CLK4 | 8 | Ribosomal protein S6 kinase 1 | 18 |
| Dual specificity protein kinase TTK | 9 | Ribosomal protein S6 kinase alpha 1 | 6 |
| Dual specificity protein phosphatase 26 | 13 | Ribosomal protein S6 kinase alpha 3 | 28 |
| Dual specificity protein phosphatase 3 | 46 | Ribosomal protein S6 kinase alpha 5 | 7 |
| Dual specificity tyrosine-phosphorylation-regulated kinase 1A | 23 | Ribosome maturation protein SDO1 | 12 |
| dUTP pyrophosphatase | 53 | Ricin | 37 |
| Dynamin-1 | 133 | RNase L | 9 |
| Ecdysone receptor | 20 | Ryanodine receptor 1 | 7 |
| Egl nine homolog 1 | 142 | S-100 protein beta chain | 5 |
| Egl nine homolog 3 | 7 | S-adenosylhomocysteine nucleosidase | 28 |
| Elastase 2A | 3 | S-adenosylmethionine decarboxylase 1 | 3 |
| Elongation of very long chain fatty acids protein 6 | 93 | Sarcoplasmic/endoplasmic reticulum calcium ATP-ase | 8 |
| Endoplasmic reticulum-associated amyloid beta-peptide-binding protein | 42 | Sarcoplasmic/endoplasmic reticulum calcium ATPase 1 | 5 |
| Endothelial lipase | 4 | Sarcoplasmic/endoplasmic reticulum calcium ATPase 2 | 8 |
| Endothelin receptor ET-A | 314 | SARS coronavirus 3C-like proteinase | 74 |
| Endothelin receptor type A | 36 | Scavenger receptor class B member 1 | 38 |
| Endothelin-converting enzyme 1 | 223 | Scytalone dehydratase | 9 |
| Enoyl-[acyl-carrier-protein] reductase | 109 | Secreted effector protein | 28 |
| Enoyl-[acyl-carrier-protein] reductase [NADH] | 3 | Secreted frizzled-related protein 1 | 20 |
| Envelope polyprotein GP160 | 12 | Seed lipoxygenase-1 | 8 |
| Ephrin type-A receptor 2 | 3 | Selectin E | 25 |
| Ephrin type-A receptor 4 | 6 | Sentrin-specific protease 1 | 69 |
| Ephrin type-B receptor 2 | 9 | Serine racemase | 11 |
| Ephrin type-B receptor 3 | 33 | Serine/threonine-protein kinase AKT | 254 |
| Ephrin type-B receptor 4 | 125 | Serine/threonine-protein kinase AKT2 | 8 |
| Epidermal growth factor receptor erbB1 | 632 | Serine/threonine-protein kinase AKT3 | 33 |
| Epoxide hydratase | 364 | Serine/threonine-protein kinase Aurora-A | 218 |
| Epoxide hydrolase 1 | 23 | Serine/threonine-protein kinase B-raf | 178 |
| Equilibrative nucleoside transporter 1 | 172 | Serine/threonine-protein kinase Chk1 | 491 |
| ERO1-like protein alpha | 9 | Serine/threonine-protein kinase Chk2 | 107 |
| Estradiol 17-beta-dehydrogenase 1 | 12 | Serine/threonine-protein kinase EEF2K | 12 |
| Estradiol 17-beta-dehydrogenase 2 | 93 | Serine/threonine-protein kinase NEK2 | 29 |
| Estradiol 17-beta-dehydrogenase 3 | 34 | Serine/threonine-protein kinase PAK 1 | 4 |
| Estrogen receptor | 420 | Serine/threonine-protein kinase PIM1 | 108 |
| Estrogen receptor alpha | 155 | Serine/threonine-protein kinase PIM2 | 20 |
| Estrogen receptor beta | 26 | Serine/threonine-protein kinase PLK1 | 126 |
| Estrogen sulfotransferase | 15 | Serine/threonine-protein kinase RAF | 117 |
| Estrogen-related receptor alpha | 47 | Serine/threonine-protein kinase Sgk1 | 3 |
| Estrogen-related receptor gamma | 4 | Serine/threonine-protein kinase TBK1 | 15 |
| Eukaryotic initiation factor 4A-I | 4 | Serine/threonine-protein kinase/endoribonuclease IRE1 | 25 |
| Eukaryotic translation initation factor | 8 | Serine/threonine-protein phosphatase | 3 |
| Eukaryotic translation initiation factor 2-alpha kinase 1 | 27 | Serotonin (5-HT) receptor | 21 |
| Eukaryotic translation initiation factor 2-alpha kinase 3 | 9 | Serotonin 1a (5-HT1a) receptor | 444 |
| Excitatory amino acid transporter 1 | 3 | Serotonin 1b (5-HT1b) receptor | 25 |
| Excitatory amino acid transporter 3 | 12 | Serotonin 1d (5-HT1d) receptor | 28 |
| Exportin-1 | 5 | Serotonin 1f (5-HT1f) receptor | 9 |
| Eyes absent homolog 2 | 20 | Serotonin 2 (5-HT2) receptor | 79 |
| Falcipain 2 | 19 | Serotonin 2a (5-HT2a) receptor | 90 |
| Farnesyl diphosphate synthase | 11 | Serotonin 2b (5-HT2b) receptor | 127 |
| Farnesyltransferase | 10 | Serotonin 2c (5-HT2c) receptor | 83 |
| Fatty acid binding protein adipocyte | 46 | Serotonin 3 (5-HT3) receptor | 316 |
| Fatty acid synthase | 65 | Serotonin 3a (5-HT3a) receptor | 133 |
| Fatty acid transport protein 4 | 7 | Serotonin 4 (5-HT4) receptor | 107 |
| Ferritin light chain | 109 | Serotonin 5a (5-HT5a) receptor | 43 |
| Fibonectin-binding protein C | 9 | Serotonin 6 (5-HT6) receptor | 1097 |
| Fibroblast growth factor 22 | 76 | Serotonin 7 (5-HT7) receptor | 255 |
| Fibroblast growth factor receptor 1 | 46 | Serotonin transporter | 167 |
| Fibroblast growth factor receptor 2 | 12 | Serum albumin | 68 |
| Fibroblast growth factor receptor 3 | 9 | SHC-transforming protein 1 | 36 |
| FK506 binding protein 12 | 68 | Short transient receptor potential channel 4 | 3 |
| FK506-binding protein 1A | 208 | Sialidase 3 | 55 |
| Flap endonuclease 1 | 21 | Sigma opioid receptor | 230 |
| Focal adhesion kinase 1 | 131 | Sigma-1 receptor | 16 |
| Follicle stimulating hormone receptor | 77 | Signal transducer and activator of transcription 3 | 317 |
| Folylpoly-gamma-glutamate synthetase | 14 | Signal transducer and activator of transcription 6 | 52 |
| Formyl peptide receptor 1 | 90 | Small conductance calcium-activated potassium channel | 25 |
| Free fatty acid receptor 1 | 95 | Small conductance calcium-activated potassium channel protein 3 | 36 |
| Fructose-1,6-bisphosphatase | 163 | Smoothened homolog | 78 |
| Fructose-1,6-bisphosphatase 1 | 16 | Snake venom metalloproteinase Bap1 | 15 |
| Furin | 61 | Sodium channel alpha subunits; brain (Types I, II, III) | 8 |
| G protein-coupled receptor 44 | 523 | Sodium channel protein type II alpha subunit | 22 |
| GABA B receptor | 3 | Sodium channel protein type IV alpha subunit | 28 |
| GABA receptor rho-1 subunit | 6 | Sodium channel protein type IX alpha subunit | 1163 |
| GABA transporter 1 | 96 | Sodium channel protein type V alpha subunit | 35 |
| GABA-A receptor; alpha-3/beta-3/gamma-2 | 4 | Sodium channel protein type VIII alpha subunit | 16 |
| GABA-A receptor; anion channel | 600 | Sodium channel protein type X alpha subunit | 22 |
| GABA-B receptor | 6 | Sodium/calcium exchanger 1 | 25 |
| GABA-B receptor 1 | 5 | Sodium/glucose cotransporter 1 | 17 |
| Galactokinase | 29 | Sodium/glucose cotransporter 2 | 361 |
| Galanin receptor 3 | 16 | Sodium/hydrogen exchanger | 110 |
| Galectin-1 | 3 | Sodium/hydrogen exchanger 1 | 161 |
| Galectin-3 | 9 | Sodium/iodide cotransporter | 6 |
| Gamma-amino-N-butyrate transaminase | 39 | Sodium/potassium-transporting ATPase | 34 |
| Gamma-hydroxybutyrate receptor | 3 | Solute carrier family 12 member 5 | 46 |
| Gamma-secretase | 194 | Solute carrier family 22 member 1 | 6 |
| Gamma-secretase subunit PEN-2 | 10 | Solute carrier family 22 member 6 | 3 |
| GAR transformylase | 3 | Solute carrier family 22 member 8 | 4 |
| Geminin | 273 | Solute carrier family 28 member 3 | 12 |
| Genome polyprotein | 34 | Solute carrier organic anion transporter family member 1A1 | 3 |
| Geranylgeranyl transferase type I | 42 | Solute carrier organic anion transporter family member 1B1 | 3 |
| Ghrelin receptor | 371 | Somatostatin receptor | 14 |
| Glucagon receptor | 190 | Somatostatin receptor 2 | 52 |
| Glucagon-like peptide 1 receptor | 255 | Somatostatin receptor 3 | 35 |
| Glucagon-like peptide 2 receptor | 27 | Somatostatin receptor 4 | 5 |
| Glucocorticoid receptor | 125 | Somatostatin receptor 5 | 27 |
| Glucokinase regulatory protein | 76 | Sonic hedgehog protein | 71 |
| Glucose-6-phosphatase | 27 | Sorbitol dehydrogenase | 15 |
| Glucose-6-phosphate 1-dehydrogenase | 5 | Sortase | 41 |
| Glucose-6-phosphate translocase | 5 | Spermidine synthase | 9 |
| Glucose-dependent insulinotropic receptor | 41 | Sphingomyelin phosphodiesterase | 8 |
| Glutamate (NMDA) receptor subunit zeta 1 | 189 | Sphingosine 1-phosphate receptor Edg-1 | 156 |
| Glutamate [NMDA] receptor | 5 | Sphingosine 1-phosphate receptor Edg-3 | 7 |
| Glutamate [NMDA] receptor subunit epsilon 2 | 57 | Sphingosine 1-phosphate receptor Edg-6 | 123 |
| Glutamate [NMDA] receptor subunit epsilon 3 | 13 | Sphingosine kinase 1 | 51 |
| Glutamate carboxypeptidase II | 142 | Sphingosine kinase 2 | 8 |
| Glutamate NMDA receptor | 510 | Sphingosine-1-phosphate lyase 1 | 14 |
| Glutamate NMDA receptor; Grin1/Grin2a | 5 | Squalene monooxygenase | 42 |
| Glutamate NMDA receptor; GRIN1/GRIN2B | 27 | Squalene synthetase | 270 |
| Glutamate receptor ionotropic AMPA | 23 | Squalene-hopene cyclase | 15 |
| Glutamate receptor ionotropic kainate 1 | 55 | Steroid 5-alpha-reductase | 63 |
| Glutamate receptor ionotropic, AMPA | 161 | Steroid 5-alpha-reductase 1 | 78 |
| Glutamate receptor ionotropic, AMPA 2 | 27 | Steroid 5-alpha-reductase 2 | 40 |
| Glutamate receptor ionotropic, AMPA 4 | 43 | Sterol regulatory element-binding protein 2 | 21 |
| Glutaminase kidney isoform, mitochondrial | 117 | Steryl-sulfatase | 171 |
| Glutaminyl-peptide cyclotransferase | 102 | Strictosidine synthase | 4 |
| Glutamyl endopeptidase | 3 | Subtilisin-like protease | 27 |
| Glutathione reductase | 25 | Succinate receptor 1 | 6 |
| Glutathione S-transferase A1 | 11 | Sulfonylurea receptor 2, Kir6.2 | 17 |
| Glutathione S-transferase Pi | 44 | Sulfonylurea receptors; K-ATP channels | 93 |
| Glutathione transferase omega 1 | 4 | Survival motor neuron protein | 25 |
| Glyceraldehyde-3-phosphate dehydrogenase cytosolic | 23 | Syk protein | 33 |
| Glyceraldehyde-3-phosphate dehydrogenase liver | 175 | Synaptic vesicular amine transporter | 35 |
| Glyceraldehyde-3-phosphate dehydrogenase, glycosomal | 22 | Tankyrase-2 | 9 |
| Glycine transporter 1 | 210 | Taq polymerase 1 | 4 |
| Glycine transporter 2 | 49 | TAR DNA-binding protein 43 | 272 |
| Glycogen phosphorylase, muscle form | 167 | T-cell surface antigen CD4 | 19 |
| Glycogen synthase kinase-3 | 23 | T-complex protein 1 subunit beta | 7 |
| Glycogen synthase kinase-3 alpha | 38 | Telomerase reverse transcriptase | 140 |
| Glycogen synthase kinase-3 beta | 538 | Testis-specific androgen-binding protein | 19 |
| Glycoprotein hormones alpha chain | 77 | Testis-specific serine/threonine-protein kinase 1 | 6 |
| Glycosyltransferase-like protein LARGE1 | 7 | Tetanus toxin | 17 |
| Glycylpeptide N-tetradecanoyltransferase | 11 | TGF-beta receptor type I | 41 |
| Glyoxalase I | 50 | Thermolysin | 129 |
| Gonadotropin-releasing hormone receptor | 728 | Thiopurine S-methyltransferase | 11 |
| G-protein coupled bile acid receptor 1 | 80 | Thioredoxin glutathione reductase | 48 |
| G-protein coupled receptor 55 | 10 | Thioredoxin peroxidase | 6 |
| G-protein coupled receptor kinase 2 | 7 | Thioredoxin reductase 1, cytoplasmic | 113 |
| Granulocyte colony stimulating factor receptor | 4 | Thrombin | 1194 |
| Granzyme B | 9 | Thrombopoietin receptor | 141 |
| Group IID secretory phospholipase A2 | 3 | Thromboxane A2 receptor | 99 |
| Group X secretory phospholipase A2 | 13 | Thromboxane-A synthase | 163 |
| Growth factor receptor-bound protein 2 | 135 | Thymidine kinase | 6 |
| Guanine deaminase | 3 | Thymidine kinase, cytosolic | 4 |
| Guanine nucleotide-binding protein G(i), alpha-1 subunit | 12 | Thymidine phosphorylase | 69 |
| Guanine nucleotide-binding protein G(s), subunit alpha | 1146 | Thymidylate kinase | 28 |
| Heart phosphodiesterase | 4 | Thymidylate synthase | 47 |
| Heat shock factor protein 1 | 10 | Thyroid hormone receptor | 17 |
| Heat shock protein HSP 90 (HSP82) | 9 | Thyroid hormone receptor alpha | 13 |
| Heat shock protein HSP 90-alpha | 22 | Thyroid hormone receptor beta-1 | 82 |
| Heat shock protein HSP 90-beta | 68 | Thyroid stimulating hormone receptor | 28 |
| Heat shock protein HSP90 | 41 | Thyrotropin-releasing hormone receptor 2 | 3 |
| Hemagglutinin-neuraminidase | 7 | Tissue-type plasminogen activator | 15 |
| Hematopoietic cell protein-tyrosine phosphatase 70Z-PEP | 64 | TNF-alpha | 74 |
| Hematopoietic prostaglandin D synthase | 30 | Toll-like receptor 4 | 68 |
| Heparanase | 60 | Toll-like receptor 7 | 13 |
| Hepatitis C virus NS3 protease/helicase | 266 | Toll-like receptor 8 | 9 |
| Hepatitis C virus NS5B RNA-dependent RNA polymerase | 499 | Toll-like receptor 9 | 4 |
| Hepatitis C virus polyprotein | 94 | Trace amine-associated receptor 1 | 20 |
| Hepatitis C virus serine protease, NS3/NS4A | 66 | TRAF2- and NCK-interacting kinase | 5 |
| Hepatocyte growth factor receptor | 422 | Trans-cinnamate 4-monooxygenase | 20 |
| HERG | 175 | Transcription factor HES-1 | 3 |
| Hexokinase | 17 | Transcriptional activator protein luxR | 6 |
| Hexokinase type IV | 133 | Transcriptional regulator ERG | 10 |
| Histamine H1 receptor | 76 | Transforming protein p21/H-Ras-1 | 39 |
| Histamine H2 receptor | 42 | Transforming protein RhoA | 32 |
| Histamine H3 receptor | 689 | Transient receptor potential cation channel subfamily A member 1 | 125 |
| Histamine H4 receptor | 365 | Transient receptor potential cation channel subfamily M member 8 | 50 |
| Histidine biosynthesis bifunctional protein HisB | 3 | Transient receptor potential cation channel subfamily V member 4 | 20 |
| Histidine-rich protein | 6 | Transient receptor potential M8 protein | 34 |
| Histone acetyltransferase GCN5 | 44 | Transitional endoplasmic reticulum ATPase | 93 |
| Histone acetyltransferase p300 | 3 | Translocator protein | 3 |
| Histone acetyltransferase PCAF | 31 | Transmembrane protease serine 11D | 25 |
| Histone deacetylase | 170 | Transmembrane protease serine 4 | 14 |
| Histone deacetylase (HDAC1 and HDAC2) | 67 | Trans-sialidase | 14 |
| Histone deacetylase 1 | 124 | Transthyretin | 177 |
| Histone deacetylase 2 | 7 | Tripeptidyl aminopeptidase | 71 |
| Histone deacetylase 6 | 7 | tRNA-guanine transglycosylase | 6 |
| Histone deacetylase 8 | 69 | Trypanothione reductase | 149 |
| Histone deacetylase HD2 | 14 | Trypsin I | 20 |
| Histone-arginine methyltransferase CARM1 | 90 | Tryptase | 63 |
| Histone-lysine N-methyltransferase EZH2 | 34 | Tryptase beta-1 | 36 |
| Histone-lysine N-methyltransferase MLL | 30 | Tryptophan 5-hydroxylase 1 | 22 |
| Histone-lysine N-methyltransferase SETD7 | 27 | Tubulin | 51 |
| Histone-lysine N-methyltransferase, H3 lysine-79 specific | 19 | Tubulin alpha chain | 41 |
| Histone-lysine N-methyltransferase, H3 lysine-9 specific 3 | 166 | Tubulin alpha-1 chain | 6 |
| HMG-CoA reductase | 127 | Tubulin beta chain | 84 |
| Hormone-sensitive lipase | 16 | Tubulin beta-1 chain | 13 |
| HTH-type transcriptional regulator EthR | 33 | Tumor necrosis factor receptor R1 | 42 |
| Human herpes virus 5 capsid protein P40 | 101 | Tumor necrosis factor receptor superfamily member 10B | 3 |
| Human herpesvirus 1 protease | 12 | Type III iodothyronine deiodinase | 4 |
| Human herpesvirus 5 DNA polymerase | 6 | Type-1 angiotensin II receptor | 49 |
| Human herpesvirus 6 DNA polymerase | 22 | Type-1A angiotensin II receptor | 33 |
| Human immunodeficiency virus type 1 integrase | 68 | Type-1B angiotensin II receptor | 153 |
| Human immunodeficiency virus type 1 protease | 1701 | Tyrosinase | 229 |
| Human immunodeficiency virus type 1 reverse transcriptase | 450 | Tyrosine kinase non-receptor protein 2 | 33 |
| Human papillomavirus regulatory protein E2 | 25 | Tyrosine-protein kinase ABL | 32 |
| Human rhinovirus A protease | 88 | Tyrosine-protein kinase BRK | 14 |
| Huntingtin | 26 | Tyrosine-protein kinase BTK | 52 |
| Hyaluronidase-1 | 4 | Tyrosine-protein kinase CSK | 69 |
| Hydroxycarboxylic acid receptor 2 | 94 | Tyrosine-protein kinase FYN | 7 |
| Hypoxia-inducible factor 1 alpha | 42 | Tyrosine-protein kinase ITK/TSK | 244 |
| Hypoxia-inducible factor 1-alpha inhibitor | 7 | Tyrosine-protein kinase JAK2 | 49 |
| Hypoxia-inducible factor prolyl 4-hydroxylase | 28 | Tyrosine-protein kinase JAK3 | 43 |
| IAA-amino acid hydrolase ILR1-like 4 | 4 | Tyrosine-protein kinase LCK | 513 |
| IAG-nucleoside hydrolase | 7 | Tyrosine-protein kinase receptor FLT3 | 56 |
| IgE Fc receptor, alpha-subunit | 11 | Tyrosine-protein kinase receptor RET | 93 |
| Ileal bile acid transporter | 162 | Tyrosine-protein kinase receptor TYRO3 | 41 |
| Indoleamine 2,3-dioxygenase | 194 | Tyrosine-protein kinase receptor UFO | 15 |
| Indolethylamine N-methyltransferase | 5 | Tyrosine-protein kinase SRC | 623 |
| Induced myeloid leukemia cell differentiation protein Mcl-1 | 91 | Tyrosine-protein kinase SYK | 104 |
| Influenza A virus Hemagglutinin | 13 | Tyrosine-protein kinase TEC | 9 |
| Inhibitor of apoptosis protein 3 | 108 | Tyrosine-protein kinase TIE-2 | 20 |
| Inhibitor of nuclear factor kappa B kinase beta subunit | 97 | Tyrosine-protein kinase V-ABL | 27 |
| Inhibitor of nuclear factor kappa B kinase epsilon subunit | 10 | Tyrosine-protein kinase ZAP-70 | 53 |
| Inosine-5'-monophosphate dehydrogenase | 56 | Tyrosine-protein phosphatase non-receptor type 5 | 42 |
| Inosine-5'-monophosphate dehydrogenase 2 | 192 | Tyrosyl-DNA phosphodiesterase 1 | 73 |
| Inositol monophosphatase 1 | 21 | Tyrosyl-DNA phosphodiesterase 2 | 9 |
| Inositol phosphorylceramide synthase | 3 | Tyrosyl-tRNA synthetase | 29 |
| Inositol-1(or 4)-monophosphatase 1 | 22 | Ubiquitin carboxyl-terminal hydrolase 2 | 7 |
| Insulin receptor | 25 | Ubiquitin carboxyl-terminal hydrolase 7 | 3 |
| Insulin-degrading enzyme | 17 | Ubiquitin carboxyl-terminal hydrolase isozyme L3 | 3 |
| Insulin-like growth factor binding protein 3 | 38 | Ubiquitin-like modifier-activating enzyme 1 | 10 |
| Insulin-like growth factor binding protein 5 | 9 | UDP-3-O-[3-hydroxymyristoyl] N-acetylglucosamine deacetylase | 8 |
| Insulin-like growth factor I receptor | 191 | UDP-3-O-acyl-GlcNAc deacetylase | 58 |
| Integrase | 39 | UDP-galactofuranosyl transferase GlfT2 | 6 |
| Integrin alpha2/beta1 | 41 | UDP-glucuronosyltransferase 2B7 | 3 |
| Integrin alpha-4 | 126 | UDP-N-acetylmuramate dehydrogenase | 15 |
| Integrin alpha-4/beta-1 | 325 | UDP-N-acetylmuramoylalanine--D-glutamate ligase | 20 |
| Integrin alpha-4/beta-7 | 42 | UDP-N-acetylmuramoylalanine-D-glutamyl-lysine-D-alanyl-D-alanine ligase | 19 |
| Integrin alpha-IIb | 8 | Uracil nucleotide/cysteinyl leukotriene receptor | 8 |
| Integrin alpha-IIb/beta-3 | 210 | Uracil-DNA glycosylase | 19 |
| Integrin alpha-L | 6 | Urease | 51 |
| Integrin alpha-M/beta-2 | 17 | Uridine phosphorylase 1 | 48 |
| Integrin alpha-V/beta-3 | 425 | Urokinase plasminogen activator surface receptor | 10 |
| Intercellular adhesion molecule (ICAM-1), Integrin alpha-L/beta-2 | 351 | Urokinase-type plasminogen activator | 183 |
| Interferon-induced, double-stranded RNA-activated protein kinase | 49 | Urotensin II receptor | 149 |
| Interleukin-1 receptor-associated kinase 4 | 62 | Vacuolar-type proton translocating pyrophosphatase 1 | 11 |
| Interleukin-2 receptor alpha chain | 34 | Vanilloid receptor | 781 |
| Interleukin-8 receptor A | 20 | Vascular cell adhesion protein 1 | 47 |
| Interleukin-8 receptor B | 160 | Vascular endothelial growth factor receptor | 29 |
| Interleukin-8 receptors, CXCR1/CXCR2 | 32 | Vascular endothelial growth factor receptor 2 | 564 |
| Intermediate conductance calcium-activated potassium channel protein 4 | 12 | Vasopressin V1a receptor | 110 |
| Intracellular chorismate mutase | 11 | Vasopressin V1b receptor | 157 |
| Isocitrate dehydrogenase [NADP] cytoplasmic | 182 | Vasopressin V2 receptor | 52 |
| Isoleucyl-tRNA synthetase | 16 | Vesicular acetylcholine transporter | 121 |
| Isoprenylcysteine carboxyl methyltransferase | 210 | Vesicular glutamate transporter 3 | 20 |
| Kallikrein 1 | 16 | Vitamin D receptor | 100 |
| Kallikrein 6 | 3 | Vitamin K-dependent gamma-carboxylase | 18 |
| Kappa opioid receptor | 98 | Vitronectin receptor alpha | 32 |
| Kelch-like ECH-associated protein 1 | 5 | Voltage-dependent calcium channel alpha2delta subunit | 3 |
| Ketohexokinase | 89 | Voltage-dependent L-type calcium channel subunit alpha-1C | 57 |
| KinA/Spo0F (sporulation kinase A/sporulation initiation phosphotransferase F) | 31 | Voltage-gated calcium channel alpha2/delta subunit 1 | 130 |
| Kinesin-like protein 1 | 204 | Voltage-gated L-type calcium channel | 100 |
| Kynureninase | 9 | Voltage-gated L-type calcium channel alpha-1C subunit | 16 |
| Kynurenine 3-monooxygenase | 72 | Voltage-gated L-type calcium channel alpha-1D subunit | 16 |
| Kynurenine--oxoglutarate transaminase I | 12 | Voltage-gated N-type calcium channel alpha-1B subunit | 136 |
| Lanosterol 14-alpha demethylase | 6 | Voltage-gated potassium channel subunit Kv1.3 | 238 |
| Lanosterol synthase | 41 | Voltage-gated potassium channel subunit Kv1.5 | 406 |
| Large conductance calcium-activated potassium channel | 3 | Voltage-gated potassium channel subunit Kv4.3 | 7 |
| L-cysteine:1D-myo-inositol 2-amino-2-deoxy-alpha-D-glucopyranoside ligase | 28 | Voltage-gated potassium channel subunit Kv7.1 | 43 |
| LDL receptor | 13 | Voltage-gated potassium channel subunit Kv7.2 | 106 |
| LDL-associated phospholipase A2 | 89 | Voltage-gated potassium channel, IKs; KCNQ1(Kv7.1)/KCNE1(MinK) | 14 |
| Legumain | 6 | Voltage-gated potassium channel, KQT;KCNQ2(Kv7.2)/KCNQ3(Kv7.3) | 40 |
| Lethal(3)malignant brain tumor-like protein 1 | 25 | Voltage-gated T-type calcium channel alpha-1G subunit | 342 |
| Leucine aminopeptidase | 7 | Voltage-gated T-type calcium channel alpha-1H subunit | 191 |
| Leucyl-tRNA synthetase, cytoplasmic | 4 | Voltage-gated T-type calcium channel alpha-1I subunit | 22 |
| Leukocyte adhesion glycoprotein LFA-1 alpha | 81 | WD repeat-containing protein 5 | 36 |
| Leukocyte common antigen | 23 | Werner syndrome ATP-dependent helicase | 4 |
| Leukocyte elastase | 512 | Xanthine dehydrogenase | 233 |
| Leukotriene A4 hydrolase | 66 | Zinc aminopeptidase | 7 |
| Leukotriene B4 receptor | 35 | Zinc finger protein GLI1 | 13 |

The table lists all the indications remaining in the CHEMBL database after removing singletons and doublets.


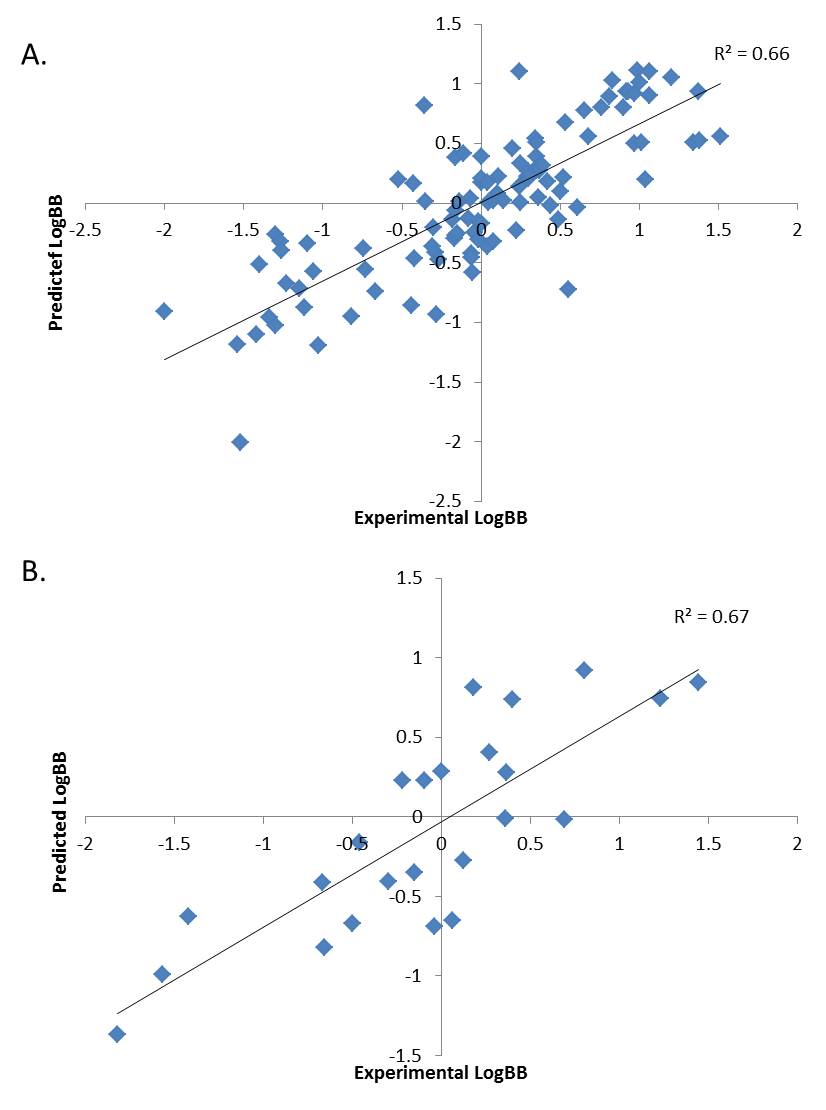


**Figure S1.** Correlation between experimental and predicted logBB values for the training and the test sets (A, B respectively).


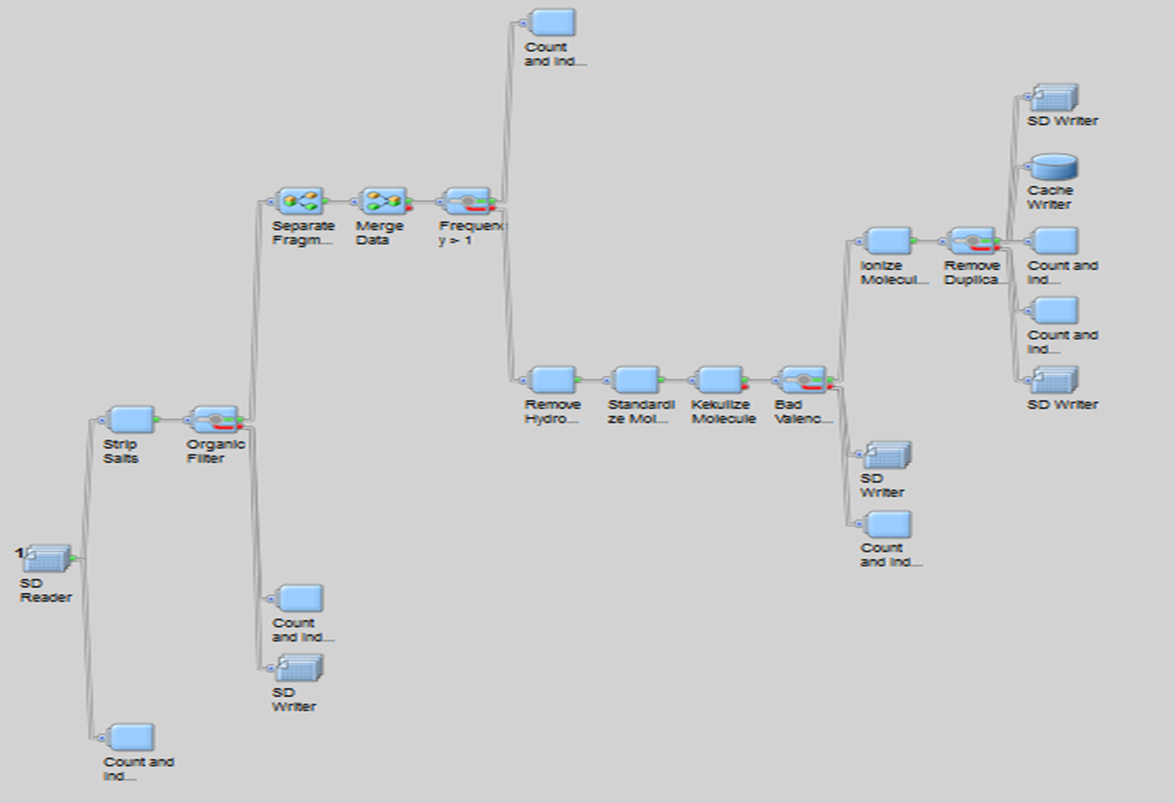


A.


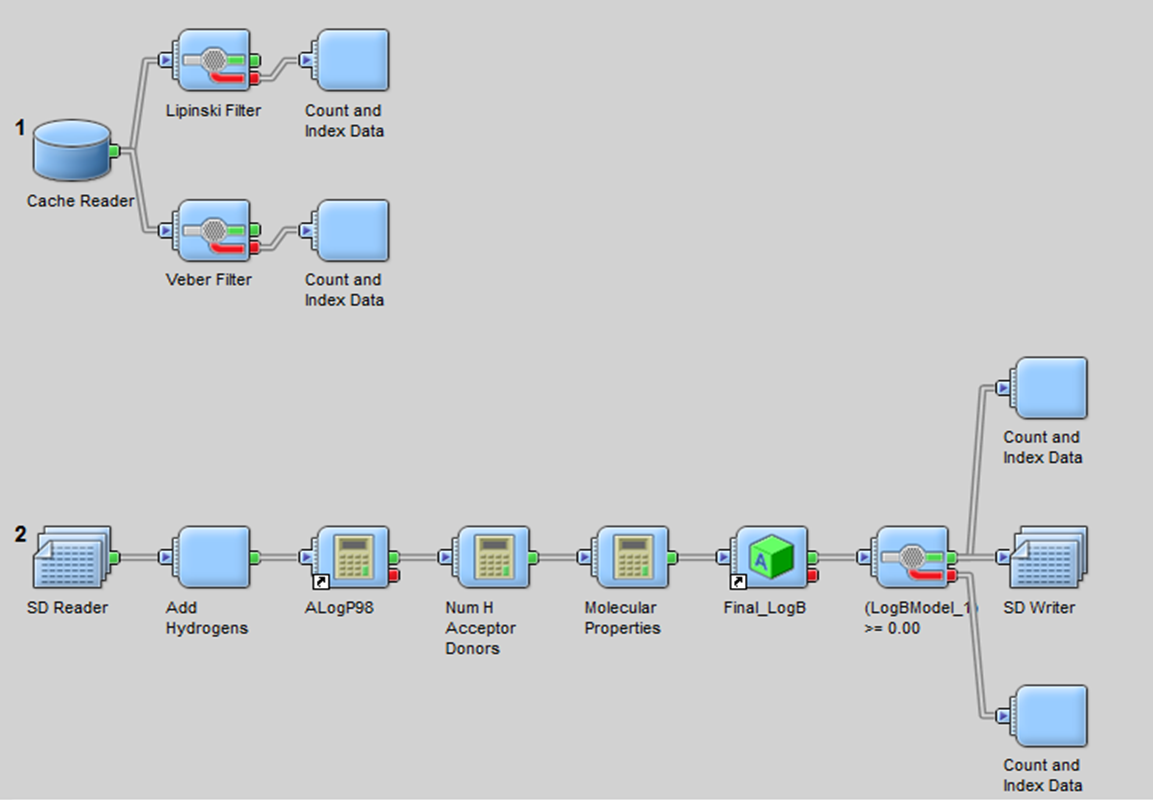


C.

B.


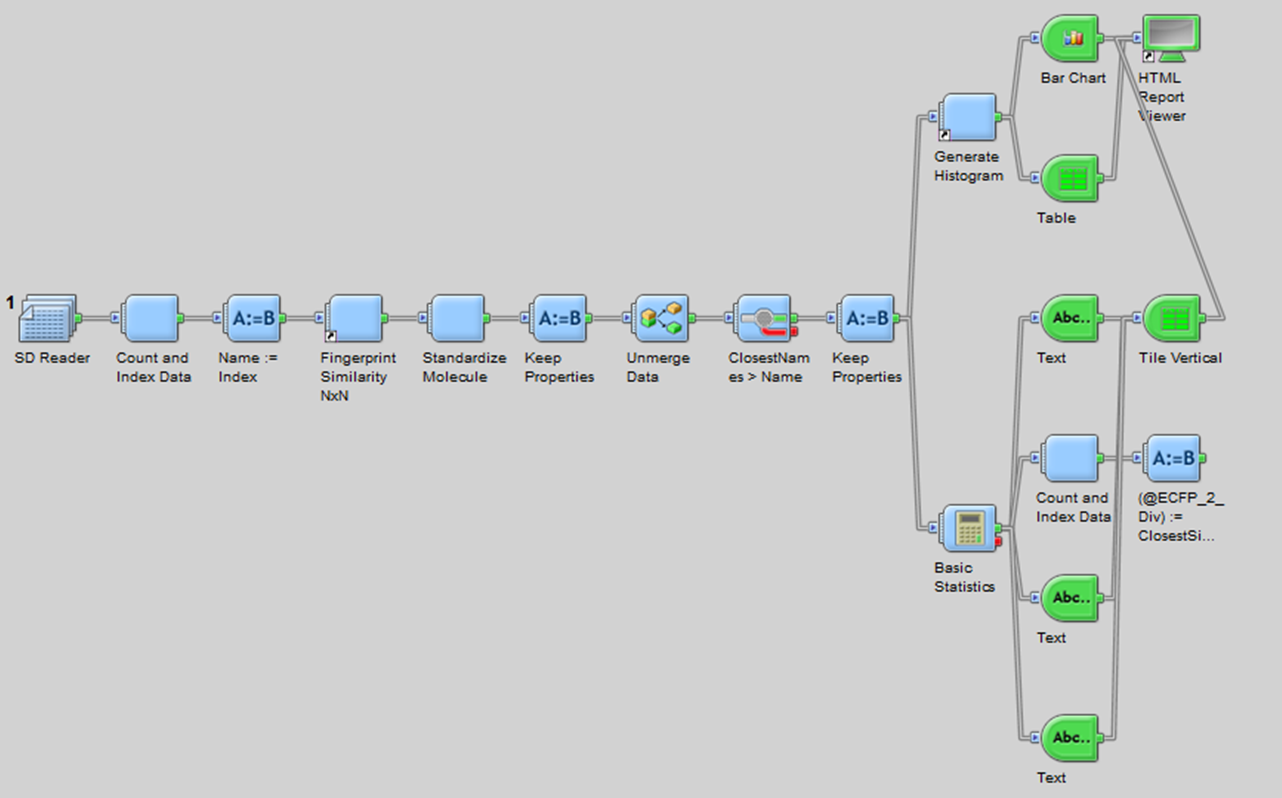


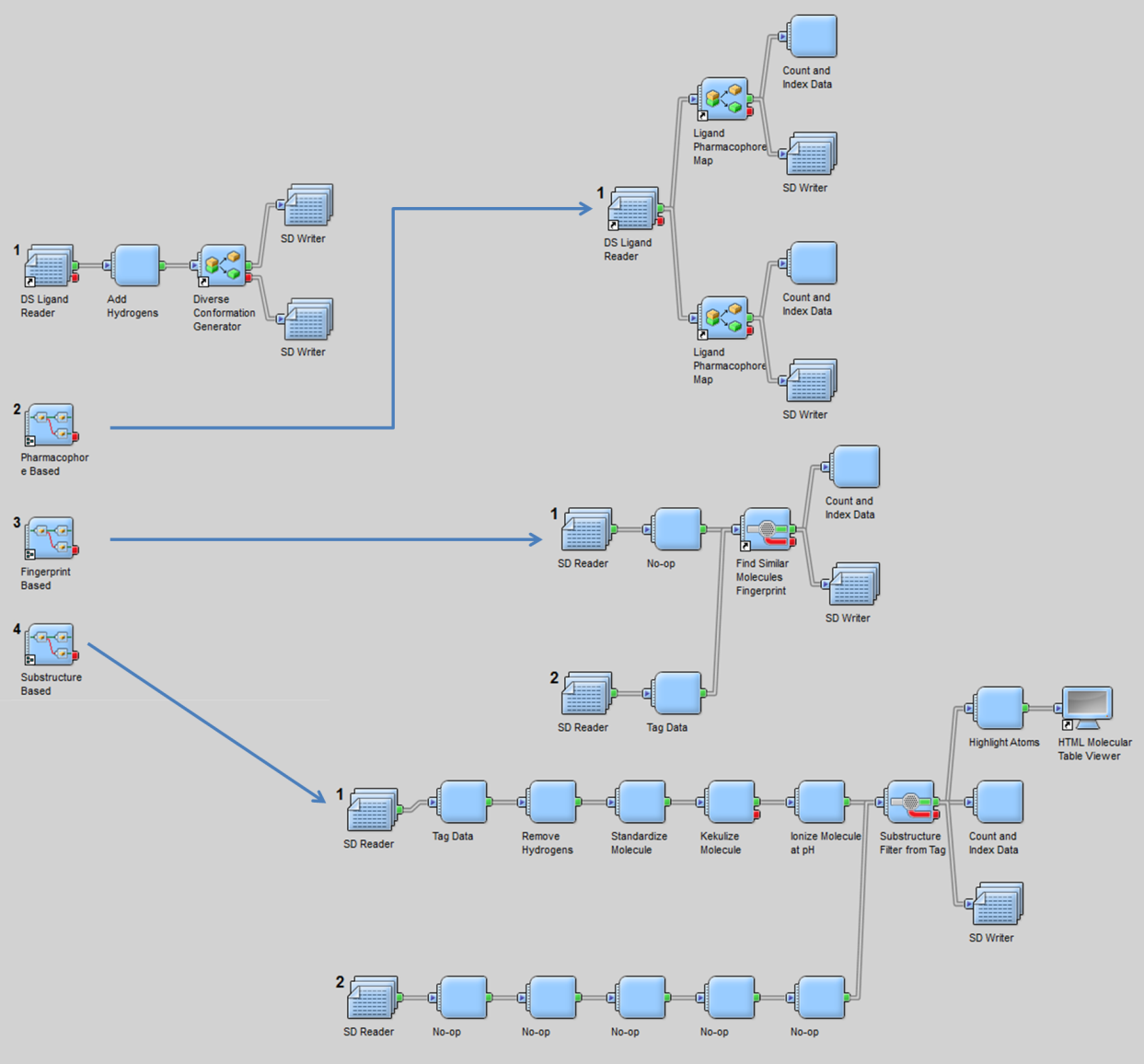


D.


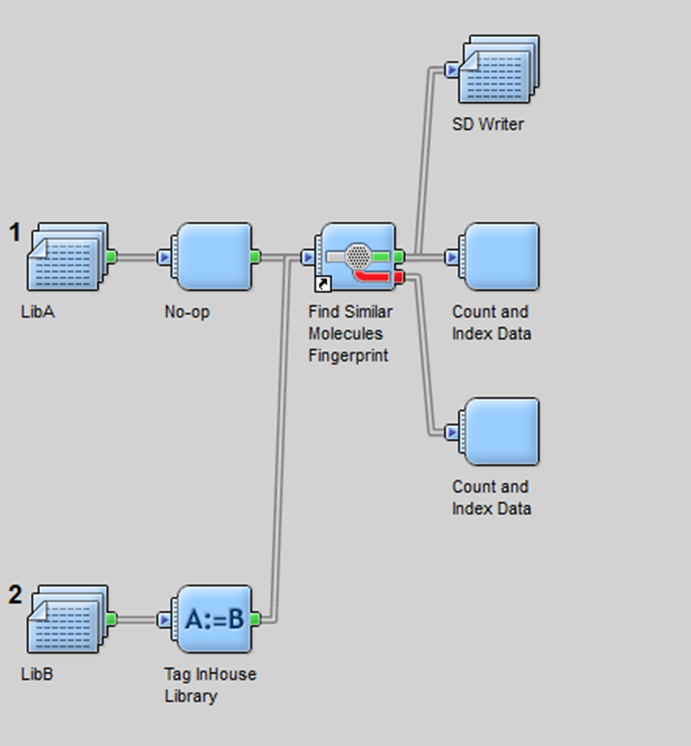


E.

**Figure S2.** Protocol steps as implementation in Pipeline Pilot. A. Data curation. B. ADME/T profiling. C. Diversity analysis. D. Similarity to known active compound analysis. E. Similarity to 'in-house' library.


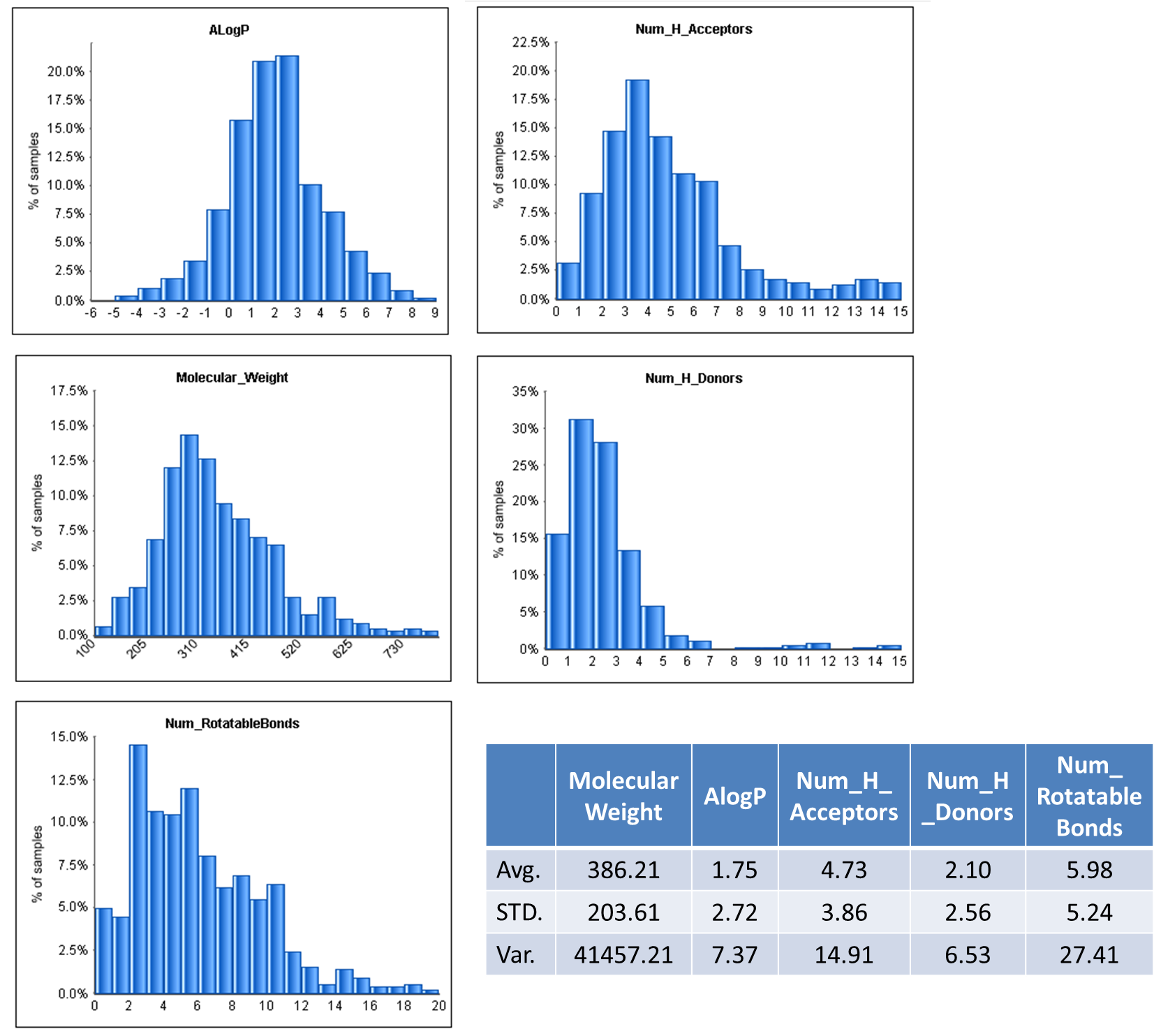


**Figure S3.** Distributions and statistics of key properties (molecular weight, AlogP, number of rotatable bonds, number of H-bond donors and acceptors) of the Drug Bank database.


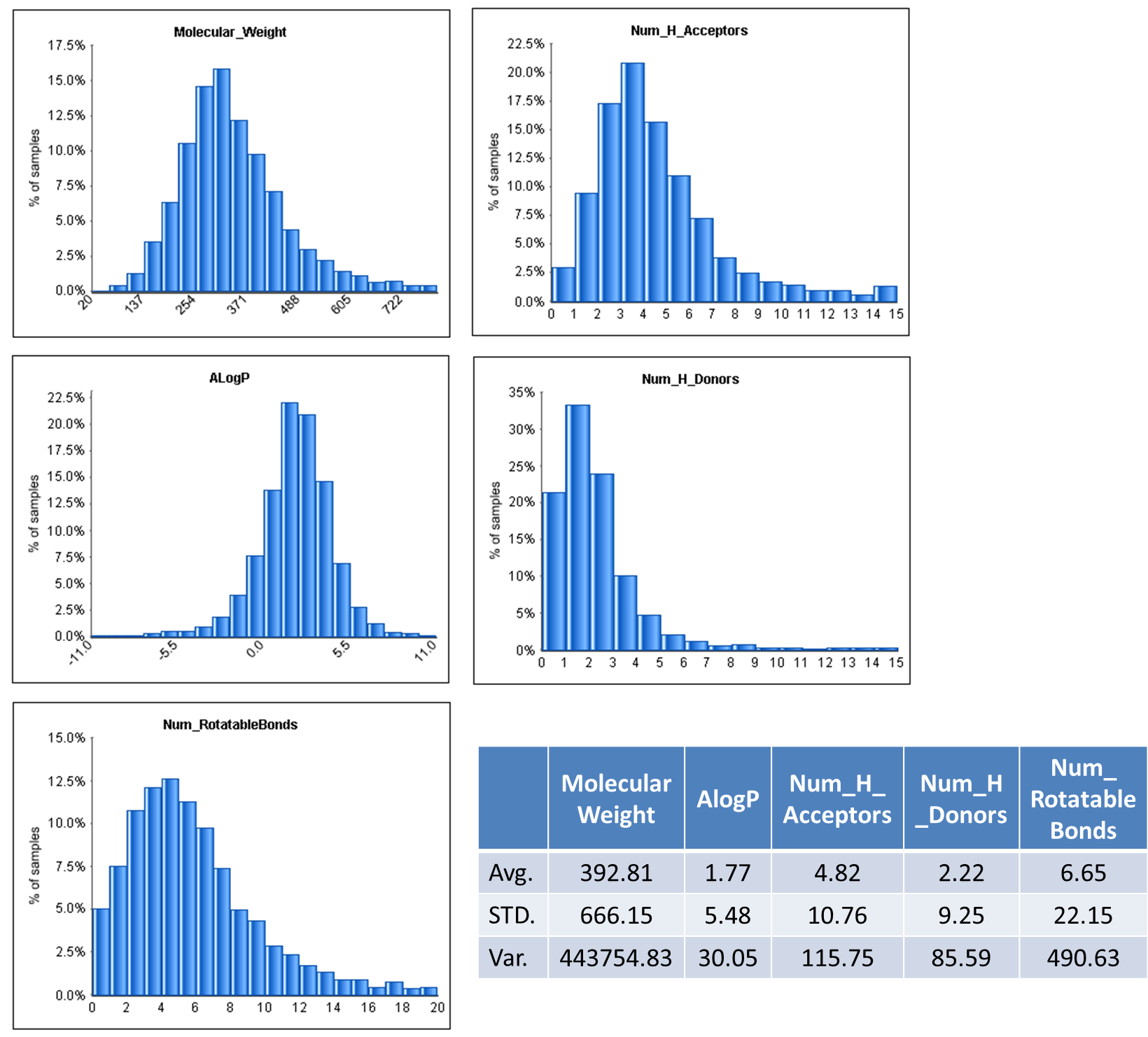


**Figure S4.** Distributions and statistics of key properties (molecular weight, AlogP, number of rotatable bonds, number of H-bond donors and acceptors) for the CMC database.


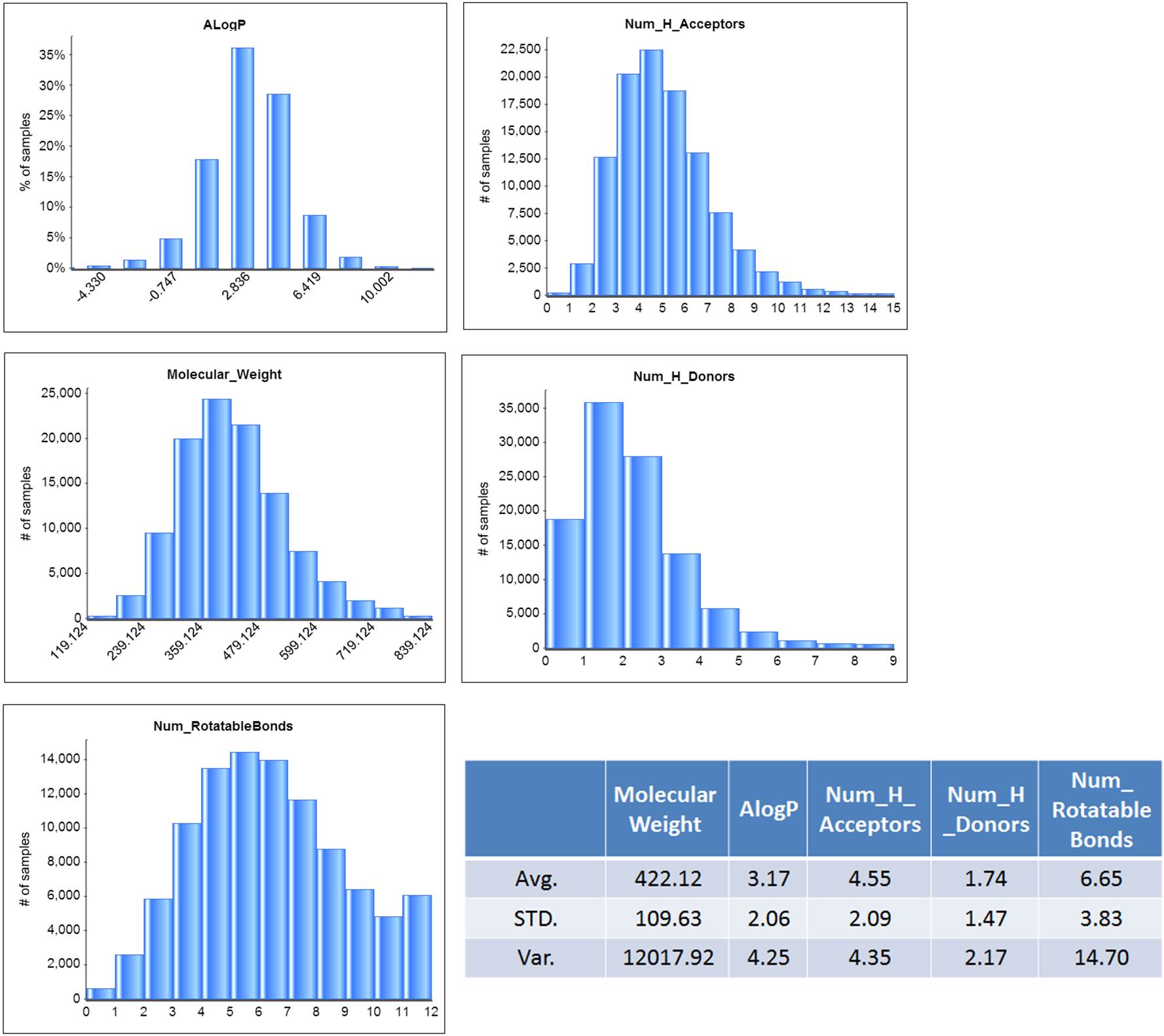


**Figure S5.** Distributions and statistics of key properties (molecular weight, AlogP, number of rotatable bonds, number of H-bond donors and acceptors) of the CHEMBL database.

**REFRENCES**

1. Rogers, D.; Brown, R. Modeling HTS Data for Activity Prediction and Iterative Screening. 2005; <http://media.accelrys.com/scitegic/protected/presentationArchive/labautomation2005.pdf>.

2. Rogers, D.; Brown, R. D.; Hahn, M., Using Extended-Connectivity Fingerprints with Laplacian-Modified Bayesian Analysis in High-Throughput Screening Follow-Up. *J. Biomol. Screen.* 2005, 10, 682-686.

3. Rogers, D.; Hahn, M., Extended-Connectivity Fingerprints. *J. Chem. Inf. Model.* 2010, 50, 742-754.
